# Peroxiredoxin 4 as a switch regulating PTEN/AKT axis in alveolar macrophages activation

**DOI:** 10.1038/s41392-025-02454-x

**Published:** 2025-10-24

**Authors:** Jia-Wei Zhou, Ying Bai, Jian-Qiang Guo, Yun-Yun Li, Ya-Feng Liu, Chao Liang, Ying-Ru Xing, Hai-Long Guo, Tian-Xiang Qi, Jing Wu, Dong Hu

**Affiliations:** 1 Department of immunology, School of Medicine, Anhui University of Science and Technology, Huainan, China; 2 Department of Laboratory Medicine, The First Affiliated Hospital of USTC, Division of Life Sciences and Medicine, University of Science and Technology of China, Hefei, China; 3 Anhui Occupational Health and Safety Engineering Laboratory, Huainan, China; 4 Key Laboratory of Industrial Dust Deep Reduction and Occupational Health and Safety of Anhui Higher Education Institutes, Huainan, China; 5 Cancer Hospital of Anhui University of Science and Technology, Huainan, China; 6 Occupational Control Hospital of Huaihe Energy Group, Huainan, China

**Keywords:** Inflammation, Respiratory tract diseases

## Abstract

Phosphatase and tensin homolog (PTEN) is a critical inhibitor of the PI3K/AKT signaling pathway, yet its direct upstream regulators remain poorly defined. In this study, we investigated the role of peroxiredoxin 4 (PRDX4) in alveolar macrophages (AMs) activation and pulmonary fibrosis. Analyses of lung tissues from silicosis patients by transcriptomic and histological analyses revealed that PRDX4 is selectively upregulated in AMs and positively correlated with profibrotic and inflammatory gene expression. Consistent results were observed in silicosis model mice, where PRDX4 expression co-localized with the macrophage marker F4/80 and correlated with fibrotic indicators. Functional studies demonstrated that macrophage-specific silencing of PRDX4 using adeno-associated virus improved lung function and reduced inflammatory infiltration and fibrosis. PRDX4 upregulation aberrantly activated AMs and promoted epithelial–mesenchymal transition and fibroblast–myofibroblast transition. Mechanistically, PRDX4 enhanced AKT/NF-κB signaling with minimal effects on PI3K. Biochemical interaction assays further demonstrated that oligomeric PRDX4 disrupted PTEN homodimer formation, with mutational analyses identifying Cys124 and Cys245 as essential residues. Notably, Conoidin A alleviated crystalline silica–induced fibrosis in mice, with its therapeutic effect likely mediated by disrupting PRDX4 oligomerization. These findings identify PRDX4 as a novel upstream regulator of PTEN, establish a mechanistic PRDX4–PTEN axis in macrophage activation, and highlight PRDX4 as a promising therapeutic target for idiopathic pulmonary fibrosis and silicosis-associated fibrosis.

## Introduction

Silicosis is a fatal disease that currently lacks specific or targeted therapeutic agents capable of reversing or halting its progression.^1–4^ Chronic pulmonary inflammation and progressive fibrosis are the key pathological features of silicosis, with fibrosis widely considered irreversible once established.^5,6^ Alveolar macrophages (AMs) serve as the primary sentinels against silica dust infiltration in pulmonary tissue, sequestering the dust particles to prevent dispersion and playing a pivotal role in silicosis pathogenesis. Exposure to crystalline silica (CS) triggers aberrant activation of AMs, leading to acute pulmonary inflammation.^7,8^ In turn, these dysregulated AMs secrete pro-inflammatory and fibrotic mediators, including IL-1α, IL-1β, TNF-α, and TGF-β, which drive the epithelial-mesenchymal transition (EMT) of alveolar type II epithelial cells (AECII) and the fibroblast-myofibroblast transition (FMT), thereby promoting pulmonary fibrogenesis.^9–11^ These highlight the critical role of aberrantly activated AMs in advancing pulmonary fibrosis.

Previous studies have demonstrated that the PI3K/AKT signaling pathway is crucial for the activation of AMs; however, the underlying mechanism is not well understood. In vitro studies indicate that the PI3K/AKT pathway is essential for the activation and proliferation of murine AMs. Moreover, in vivo inhibition of the PI3K/AKT pathway can mitigate pulmonary collagen deposition.^12,13^ Conversely, another in vivo study has shown that activation of the PI3K/AKT pathway is essential for preventing apoptosis in rat AMs and alleviating lung injury.^14^ Therefore, further investigation into the molecular mechanisms underlying PI3K/AKT-mediated activation of AMs is particularly warranted.

Phosphatase and tensin homolog (PTEN) acts as a dual-specificity phosphatase, modulating both lipids and proteins, and plays a pivotal role in regulating the equilibrium of PI3K/AKT signaling within cells.^15,16^ PTEN primarily exists in two forms within the cells: monomers, which are more susceptible to degradation and localized in the nucleus; homodimers, stabilized by disulfide bonds (S–S bonds), which serve as the active form capable of anchoring to the plasma membrane. The homodimers inhibit AKT protein phosphorylation at Thr308 by converting phosphatidylinositol-3,4,5-trisphosphate (PIP3) to phosphatidylinositol-4,5-bisphosphate (PIP2).^17,18^ However, the upstream regulatory factors modulating PTEN dimerization remain unidentified to date.^19^

Peroxiredoxin 4 (PRDX4) is an antioxidant protein expressed in pulmonary macrophages and epithelial cells that contains four cysteine residues (Cys51, Cys124, Cys148, and Cys245).^20^ It plays a key role in oxidative protein folding within the endoplasmic reticulum and in scavenging intracellular reactive oxygen species (ROS). PRDX4 is also recognized as a regulatory protein in several inflammatory diseases, including obesity, atherosclerosis, and rheumatoid arthritis.^21^ In cells, PRDX4 predominantly exists in the form of monomers and homodimers; upon oxidation by hydrogen peroxide (H₂O₂), the cysteine residues form disulfide bonds that promote the assembly of high-molecular-weight (HMW) PRDX4 oligomers.^22^ As these oligomers dissociate into dimers or monomers, they can induce redox reactions on cysteine thiol (–SH) groups of target proteins, potentially facilitating the formation of intramolecular disulfide bonds within target proteins.^23^ However, the relationship between PRDX4 and the PTEN/AKT pathway remains unclear.

Here, we observed that PRDX4 expression was elevated in AMs from both silicosis patients and mice, positively correlating with the expression of pro-inflammatory and pro-fibrotic factors. Mechanistically, we identified that HMW PRDX4 inhibited the formation of PTEN homodimers by oxidizing PTEN monomers, revealing a novel regulatory mechanism in the PTEN/AKT pathway. Point mutation of PRDX4 confirmed that Cys124 and Cys245, the two catalytic cysteine residues, were essential for PRDX4 oligomerization. Functionally, targeted knockdown of PRDX4 expression in AMs and systemic inhibition of PRDX4 activity in silicosis mice resulted in increased body weight, improved pulmonary function, and reduced fibrosis markers. In summary, PRDX4 may emerge as a potential therapeutic target for silicosis.

## Results

### PRDX4 in AMs promotes fibrosis in silicosis

**Fig. 1:**
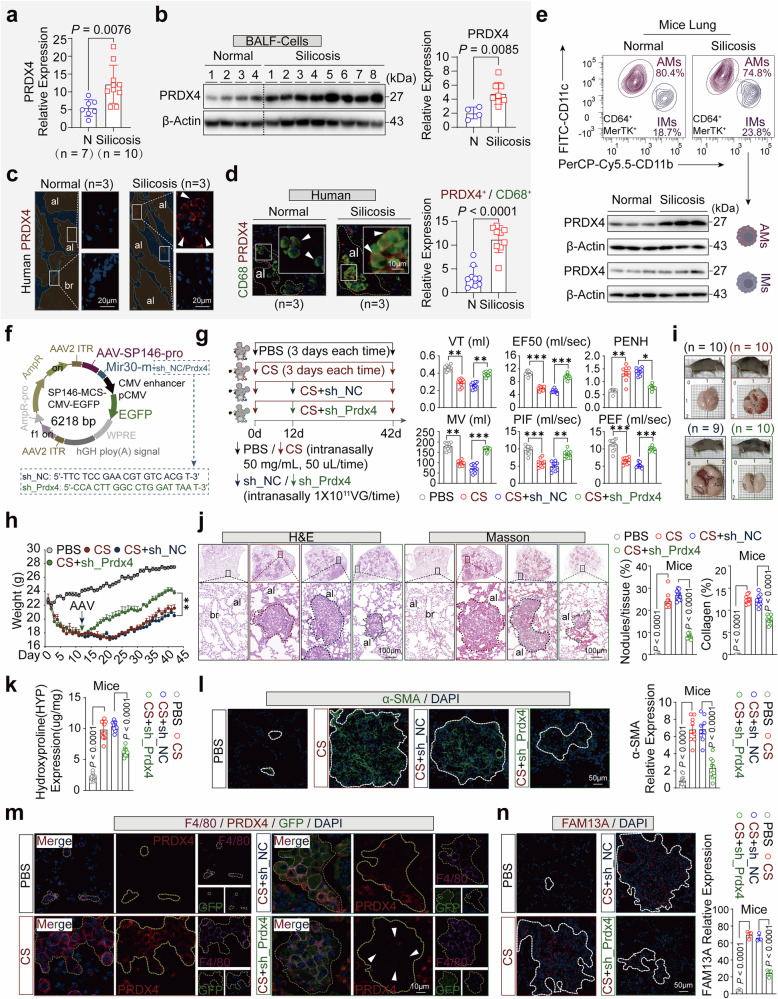
PRDX4 in AMs promotes fibrosis in silicosis. **a** The expression of PRDX4 in silicosis patients (Silicosis, n = 10) and normal controls (N, n = 7) was examined using RNA-sequencing data from the Genome Sequence Archive (GSA accession number HRA000560). **b** Protein expression of PRDX4 was quantified in the alveolar lavage fluid of healthy miners (n = 4) and silicosis patients (n = 8), with the right column displaying the analysis results. **c** Immunofluorescence (IF) analysis of PRDX4 was conducted on lung tissues from normal individuals (n = 3) and silicosis patients (n = 3). The high-power magnification of selected areas is shown on the right, with white arrows indicating PRDX4 protein expression. Yellow and blue shadings denote the lung parenchyma (including bronchi and alveoli) and interstitium (connective tissue and blood vessels), respectively. **d** Immunofluorescence (IF) staining of PRDX4 in alveolar macrophages (AMs) from both normal and silicosis patients (n = 3) was conducted. High-magnification insets of the delineated AMs are presented. White arrows denote the AMs, and the yellow dashed line delineates the pulmonary parenchyma. The histogram displays the relative PRDX4 protein to macrophage (CD68) expression ratio, with data from three fields of view per sample, each representing a single field. **e** Flow cytometry was utilized to analyze lung tissue from normal and silicosis mice, sorting CD64^+^MerTK^+^CD11c^high^CD11b^low^ AMs (pink) and CD64^+^MerTK^+^CD11c^low^CD11b^high^ IMs (purple), and assessing PRDX4 protein expression. The bar chart illustrates PRDX4 protein levels in normal (n = 3) and silicosis mice (n = 3). **f** The provided AAV plasmid map details the targeted knockdown of PRDX4 expression in lung AMs, including the sh_NC/PRDX4 sequence. **g** The flowchart outlines the construction of the mouse model and presents lung function data. Mice were nasally administered PBS (50 μL/time) and CS (50 mg, 50 μL/time) every three days for 42 days, post-anesthesia. On day 12, a single intratracheal injection of 50 μL containing 1 × 10^11^ VG of sh_NC/Prdx4 virus suspension was administered. CS, a non-toxic, odorless, white powder with particles of 5–10 μm, was dissolved in PBS to form a suspension. Lung function data for four groups of mice on day 42 are shown, quantified by mouse tidal volume (TV), expiratory flow rate at 50% of tidal volume (EF50), enhanced pause (PENH), minute ventilation (MV), peak inspiratory flow (PIF), and peak expiratory flow (PEF), with each data point representing an individual mouse. **h** A line chart illustrates the body weight changes in four groups of mice. **i** The appearance of lungs from four groups of mice is depicted: PBS (n = 10), CS (n = 10), CS+sh_NC (n = 9), and CS+sh_Prdx4 (n = 10). Damaged areas of lung tissue are delineated by red, blue, and green dashed lines. **j** Histological analysis using H&E and Masson staining of lung tissue from four groups of mice is presented. The inset shows a high-power magnification view of the delineated area, with the dashed line indicating lung nodules and collagen deposition. The right column presents the statistical analysis of nodule count and collagen scores in lung tissue, with each data point representing an individual mouse. **k** The hydroxyproline (HYP) content in lung tissue across four groups of mice is shown, with each data point representing an individual mouse. **l** IF analysis of α-SMA in lung tissue from four groups of mice is conducted. The right column displays the statistical analysis of α-SMA expression, with three mice selected for IF staining and three fields of view analyzed per mouse, each data point representing a single field of view. **m** IF of F4/80, PRDX4, and GFP in lung tissue from four groups of mice is shown. The yellow dashed line delineates the expression area of AMs, and the white arrow points to AMs with downregulated PRDX4 expression. **n** IF of FAM13A in lung tissue from four groups of mice is presented. The right column shows the statistical analysis results for FAM13A, with each data point representing an individual mouse. Scale bars: 10 µm (**d**, **m**), 20 µm (**c**), 50 µm (**l**, **n**), 100 µm (**j**), 1000 µm (**i**). al alveoli, br bronchi. Data are presented as mean ± standard error of the mean (Mean ± SEM). The boxplot (**e**) displays the median and the 25th–75th percentiles, with whiskers indicating the minimum and maximum values. Statistical significance was determined using two-tailed unpaired t-tests (**a**, **b**, **d**, **h**) and One-Way ANOVA (**g**, **j**, **k**, **l**, **n**). NS not significant; *P ≤ 0.05; **P ≤ 0.01; ***P ≤ 0.001

To identify key molecules influencing silicosis progression, we analyzed whole-genome transcriptomic sequencing data from lung tissues of silicosis patients,^24^ and discovered that the PRDX4 gene was significantly overexpressed compared to normal controls (Fig. 1a). To validate this finding, we measured PRDX4 protein levels in bronchoalveolar lavage fluid (BALF) from silicosis patients and observed remarkable upregulation (Fig. 1b). Additionally, we collected lung tissue samples from three silicosis patients and three controls during lung transplantation surgeries (Supplementary Fig. 1a). Immunofluorescence (IF) staining indicated that PRDX4 (white arrows) was highly expressed in the pulmonary parenchymal region of silicosis patients (Fig. 1c, yellow shading), whereas it was absent in the pulmonary interstitial region (Fig. 1c, blue shading). For clarity, the pulmonary parenchyma encompassed the areas surrounding and within the bronchi (br) and alveoli (al), while the pulmonary interstitium included the connective tissue and vascular regions.^25^

Under normal physiological conditions, lung immune cells predominantly reside in the pulmonary parenchyma, with alveolar macrophages (AMs) being more abundant than interstitial macrophages (IMs).^26,27^ The ratio of AMs to IMs in mice is approximately eightfold.^28^ AMs are the primary immune cells that encounter silica dust, pollutants, and pathogens entering the lung from the external environment. Upon activation, AMs proliferate extensively and recruit additional inflammatory cells into the alveolar space.^8^ This response aligns with our analysis of lung gene expression profiles from silicosis patients and mice, which revealed significant AM infiltration. (Supplementary Fig. 1c, g). Furthermore, histological analysis demonstrated marked macrophage infiltration in the pulmonary parenchyma of both silicosis patients and mice (Fig. 1d and Supplementary Fig. 1d, yellow dashed lines), alongside pronounced overexpression of PRDX4 (Fig. 1d and Supplementary Fig. 1d, white arrows). Alternatively, other immune cells, such as T cells and neutrophils, did not display elevated expression levels (Supplementary Fig. 1b, e). To confirm this finding, we isolated lung macrophages, neutrophils, T cells, and other CD45^+^ cell subsets from mice. Subsequent immunoblotting experiments revealed that PRDX4 expression was significantly elevated exclusively in macrophages (Supplementary Fig. 1f). To determine whether PRDX4 expression was exclusive to AMs, we isolated AMs and IMs from the lung tissues of mice with silicosis. AMs were approximately four times more abundant than IMs. (Fig. 1e, upper panel). Notably, PRDX4 expression was markedly higher in AMs compared to IMs in the silicosis mice (Fig. 1e, lower panel).

To determine the function of PRDX4, selectively overexpressed in AMs, we induced targeted knockdown of PRDX4 protein in murine lung AMs using an adeno-associated virus (AAV) serotype 9 vector containing SP146, a macrophage-specific promoter^29^ (sh_Prdx4) (Fig. 1f). To ensure effective viral infection and functionality in AMs, we administered CS intranasally once every 3 days until the 12th day, when AMs had achieved a sufficient density. Then we gave a single intratracheal injection of either sh_NC or sh_PRDX4 virus.^30^ Mice were sacrificed on the 42nd day (Fig. 1g). GFP tracing confirmed successful lung infection of the recombinant AAV (Supplementary Fig. 1h, i). Lung function assessments showed that mice in the CS and CS+sh_NC groups had reduced tidal volume (TV), expiratory flow at 50% of TV (EF50), minute ventilation (MV), peak inspiratory flow (PIF), and peak expiratory flow (PEF), along with heightened airway reactivity (PENH), compared to the PBS group. In contrast, the CS+sh_Prdx4 group displayed opposing trends (Fig. 1g), indicating that PRDX4 knockdown in AMs significantly improved lung function in silicosis mice. Body weight data showed that mice began to lose weight from day 2 post-CS stimulation. While the CS and CS+sh_NC groups gradually regained weight by day 21, the CS+sh_PRDX4 group began to show weight recovery as early as day 14 (Fig. 1h). Furthermore, the CS+sh_Prdx4 group displayed markedly reduced lung injury (green dashed lines), fewer nodules formed, and less collagen deposition (Fig. 1i, j). This suggests that suppressing PRDX4 expression in AMs could substantially alleviate the detrimental effects of CS on the lung and expedite the recovery of lung function. Fibrosis, a key pathological feature of silicosis, directly affects patient prognosis and survival.^31^ The improvement in lung function due to PRDX4 knockdown in AMs correlated with significantly lower levels of fibrosis biomarkers, including hydroxyproline (HYP) and α-SMA (Fig. 1k, l). FAM13A, a protein inversely correlated with lung function, is predominantly expressed in alveolar type II epithelial cells and macrophages.^32^ We also observed that the effective knockdown of PRDX4 (white arrows) in lung AMs (Fig. 1m, yellow dashed lines) significantly reduced FAM13A expression (Fig. 1n, white dashed lines), which may enhance lung function.

### PRDX4-mediated AMs activation promotes epithelial and fibroblast transformation

**Fig. 2:**
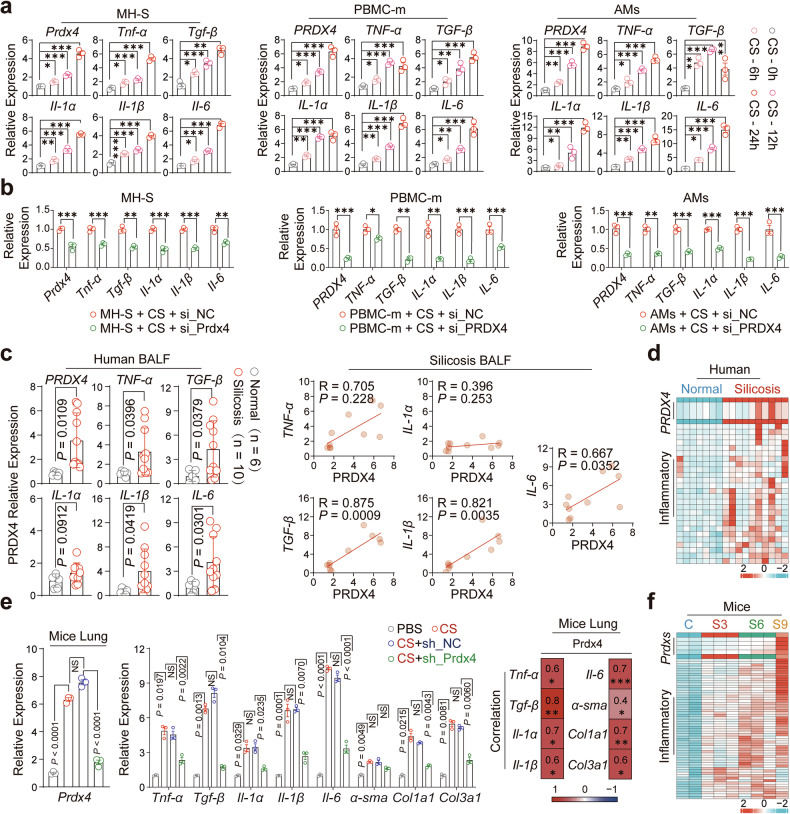
PRDX4-mediated activation of AMs promotes epithelial and fibroblast transformation. **a** The expression of PRDX4 and associated inflammatory genes in MH-S, PBMC-m, and AMs cells was assessed following exposure to CS (50 μg/cm²) at 0, 6, 12, and 24 h. This experiment was conducted three times. **b** The expression of PRDX4 and associated inflammatory genes was evaluated in MH-S, PBMC-m, and AMs cells after transfection with si_NC and si_PRDX4 and subsequent stimulation with CS (50 μg/cm²) for 24 h. The experiment was repeated three times. **c** The expression levels of *PRDX4* and related inflammatory (*TNF-α, IL-1α, IL-1β, IL-6*) and profibrotic genes (*TGF-β*) were compared in alveolar lavage fluid from healthy miners (Normal, n = 6) and silicosis patients (Silicosis, n = 10). A dot plot on the right illustrates the correlation analysis between PRDX4 and these inflammatory genes, with each dot representing an individual patient. **d** A heatmap illustrates the expression of PRDX4 and inflammation-related genes in RNA-sequencing data from lung tissues of normal individuals (n = 7) and silicosis patients (n = 10). The original data were log-transformed [log2 (Gene expression +1)] and presented in heatmap format, with green indicating low expression and red indicating high expression. **e** The expression of *Prdx4*, related inflammatory genes (*Tnf-α, Il-1α, Il-1β, IL-6*), and fibrotic genes (*Tgf-β*, α-sma, col1a1, col3a1) in lung tissues of four groups of mice is shown, with each data point representing an individual mouse. The heatmap on the right displays the correlation between *Prdx4* and the expression of related inflammatory genes, with red indicating a positive correlation, and includes data from all four groups of mice. **f** A heatmap presents the expression of peroxiredoxins (*Prdx1, Prdx3, Prdx6*) and inflammation-related genes in RNA-sequencing data from normal and silicosis mouse lung tissues. Data are presented as mean ± standard error of the mean (Mean ± SEM). Statistical significance was determined using two-tailed unpaired t-tests (**c**), One-Way ANOVA (**a**, **e**), Multiple t-tests (**b**), and Two-Way ANOVA (**e**). Correlation analysis was performed using Pearson’s test (**c**, **e**). The gene expression heatmaps (**d**, **e**) were generated using the “pheatmap” package in R (version 4.0.2). NS not significant; *P ≤ 0.05; **P ≤ 0.01; ***P ≤ 0.001

Activated AMs secrete a diverse array of inflammatory cytokines, driving pulmonary inflammation and advancing the progression of fibrosis.^8^ However, the direct link between elevated PRDX4 expression and AMs activation remains unclear. To investigate this relationship, we first isolated immune cell subsets from the lungs of silicosis mice and found a positive correlation between Prdx4 expression in AMs and the expression of pro-inflammatory (*Tnf-α*, *Il-1α*, *Il-1β*, *IL-6*) and profibrotic (*Tgf-β*) genes (Supplementary Fig. 1j). Given that pro-inflammatory and pro-fibrotic factors are mainly secreted by M1- or M2-type macrophages, respectively, we stimulated murine alveolar macrophages (MH-S) with CS for 24 h and found that CS primarily induced the differentiation of MH-S into M1-type macrophages (Supplementary Fig. 2a). Meanwhile, high expression of PRDX4 was observed in both M1- and M2-type macrophages (Supplementary Fig. 2b). We then derived macrophages from peripheral blood mononuclear cells (PBMCs) of healthy volunteers (PBMC-m) and isolated human alveolar macrophages (AMs) from the bronchoalveolar lavage fluid (BALF) of miners without respiratory disease (Supplementary Fig. 2c, d). In vitro stimulation of MH-S, peripheral blood mononuclear cell-derived macrophages (PBMC-m), and AMs with crystalline silica (CS, 50 μg/cm²) for 6, 12, and 24 h, respectively, led to cell shrinkage, progressive cell death, and a time-dependent increase in *Prdx4* gene expression, as well as the upregulation of associated pro-inflammatory (*TNF-α*, *IL-1α*, *IL-1β*, *IL-6*) and profibrotic (*TGF-β*) genes (Fig. 2a and Supplementary Fig. 2e). Knockdown of *Prdx4* significantly downregulated the mRNA level of these factors (Fig. 2b and Supplementary Fig. 2f, g).

Consistently, *Prdx4* gene expression was significantly upregulated in the BALF of silicosis patients compared to healthy miners, showing a positive correlation with pro-inflammatory and pro-fibrotic gene expression (Fig. 2c). This pattern aligns with the gene expression profile observed in lung tissue from silicosis patients (Fig. 2d and Supplementary Tables S1, 2). In addition, pulmonary function data revealed that *PRDX4* gene expression in BALF was negatively correlated with FVC and FEV1/FVC in patients with silicosis (Supplementary Fig. 3a). Furthermore, we found that PRDX4 gene expression was negatively associated with prognosis in patients with idiopathic pulmonary fibrosis (IPF) and could predict 3-year outcomes (Supplementary Fig. 3b). These findings suggest that PRDX4 may serve as a potential prognostic biomarker for patients with fibrotic lung diseases. Similarly, in the silicosis mouse model, *Prdx4* knockdown (CS+sh_PRDX4 group) significantly reduced pulmonary *Prdx4* levels and downregulated inflammation (*Tnf-α, Il-1α, Il-1β, IL-6*) and fibrosis (*Tgf-β, Col1a1, Col3a1*) markers, which correlated with improved outcomes (Fig. 2e). Furthermore, RNA sequencing of silicosis mouse lung tissues revealed a similar expression pattern in *Prdx* genes and inflammation-related pathways (Fig. 2f and Supplementary Table S3). These findings imply that PRDX4 likely acted as a promoter in the activation of AMs and the progression of silicosis.

Another key factor in the development of pulmonary fibrosis is the injury to alveolar type II epithelial cells (AECII), mediated by inflammatory factors.^9^ This damage triggers aberrant epithelial-mesenchymal transition (EMT) and fibroblast-to-myofibroblast transition (FMT), leading to excessive proliferation of fibroblasts and myofibroblasts. Consequently, these cells overproduce extracellular matrix (ECM), ultimately contributing to extensive pulmonary fibrosis.^10^ Abnormally activated AMs, as major sources of inflammatory mediators, may modulate the behavior of cells associated with fibrosis. To investigate this hypothesis, we established co-culture models of macrophages with epithelial cells (MH-S/MLE12, AMs/A549, PBMC-m/A549) and with fibroblasts (AMs/WI-38, PBMC-m/WI-38). Our findings indicated that macrophages activated by CS significantly enhanced the migration and proliferation of both murine (MLE12) and human (A549) epithelial cells while promoting EMT and upregulating COL1A1 and COL3A1 expression (Supplementary Fig. 3c, d). Similarly, activated macrophages promoted the migration and proliferation of human fibroblasts (WI-38) and increased the expression of α-SMA, a myofibroblast marker, as well as COL1A1 and COL3A1 (Supplementary Fig. 3d). These findings suggest that abnormally activated AMs can facilitate both EMT and FMT, thereby accelerating pulmonary fibrosis progression (Supplementary Fig. 3e).

### PRDX4 activates AMs through the AKT/NF-κB pathway

**Fig. 3:**
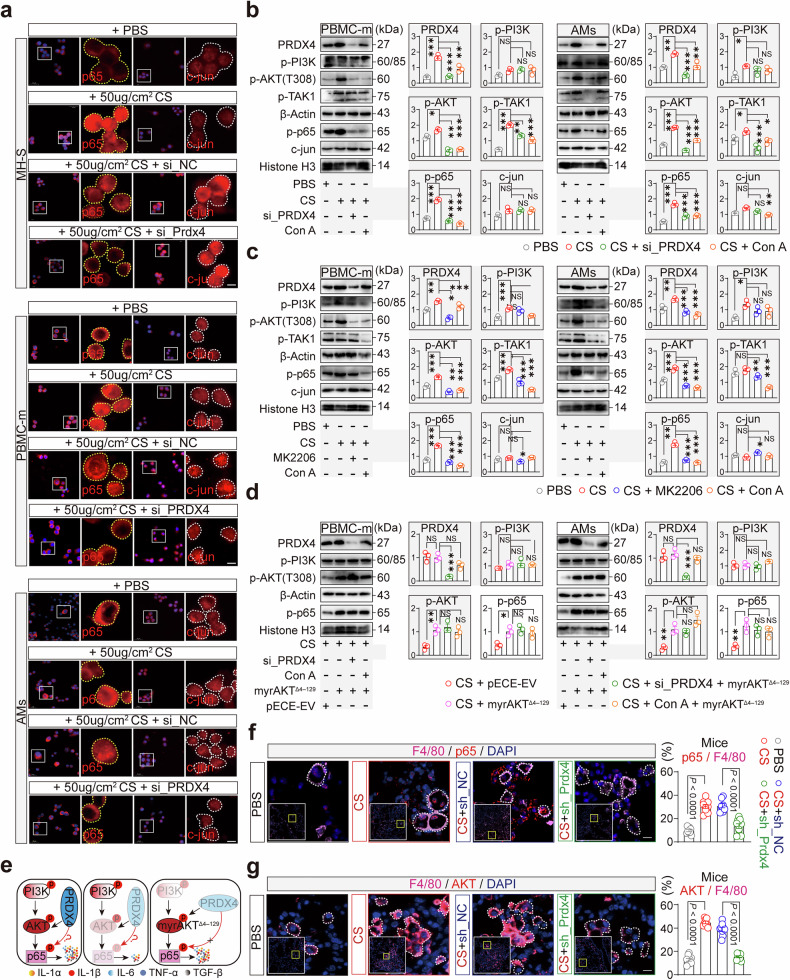
PRDX4 Positively Regulates the AKT/NF-κB Pathway in AMs. **a** Immunofluorescence (IF) staining of p65 and c-jun in the nuclei of MH-S, PBMC-m, and AMs cells was performed following transfection with si_NC and si_PRDX4 and subsequent stimulation with CS (50 μg/cm²) for 36 h. High-power magnification images of selected areas are shown on the right. Blue and white dashed lines indicate the expression areas of p65 and c-jun, respectively. The experiment was conducted three times. **b** Protein expression of PRDX4 and its regulated signaling pathway proteins, including cytoplasmic (p-PI3K, p-AKT, p-TAK1) and nuclear (p-p65, c-jun), was detected in PBMC-m and AMs cells treated with si_PRDX4 and Con A (10 μM) after 48 h of CS (50 μg/cm²) stimulation. Quantitative analysis results are presented in the right column, with the experiment repeated three times. **c** PRDX4 and its regulated signaling pathway proteins, including cytoplasmic (p-PI3K, p-AKT, p-TAK1) and nuclear (p-p65, c-jun), were detected in PBMC-m and AMs cells treated with si_PRDX4 and MK-2206 (5 μM) after 48 h of CS (50 μg/cm²) stimulation. Quantitative analysis results are presented in the right column, with the experiment repeated three times. **d** After transfection of pECE-EV and myrAKT^Δ4^^−^^129^ plasmids into PBMC-m and AMs cells, cells were treated with si_PRDX4 and MK-2206 (5 μM) for 48 h post-CS (50 μg/cm²) stimulation. Protein expression of PRDX4 and its regulatory signaling pathway proteins, including cytoplasmic (p-AKT) and nuclear (p-p65), was detected. A bar graph on the right presents the quantitative analysis of PRDX4 and related proteins, with experiments conducted three times. **e** The left and middle panels provide a schematic illustration of PRDX4 positively regulating the activation of the AKT/NF-κB pathway. The right panel shows the loss of PRDX4 regulatory function on the AKT/NF-κB pathway under conditions of excessive activation. Immunofluorescence (IF) staining of p65 (**f**) and AKT (**g**) in AMs of four groups of mice is shown, with insets displaying high-power magnification of AMs outlined by a yellow solid line. White dashed lines outline the macrophages. The right column presents the relative expression ratio of p65 and AKT proteins in mice (F4/80) macrophages, with three mice selected for IF staining and three fields of view analyzed per mouse, each point representing a field of view. Scale bars: 5 µm (**a**), 10 µm (**f**, **g**). Data are presented as mean ± standard error of the mean (Mean ± SEM). Statistical significance was determined using two-tailed unpaired t-tests (**f**, **g**) and One-Way ANOVA (**b**, **c**, **d**). NS not significant; *P ≤ 0.05; **P ≤ 0.01; ***P ≤ 0.001

To clarify the molecular mechanisms by which PRDX4 regulates inflammatory cytokine secretion in activated AMs, we utilized the TRRUST database to identify transcription factors (TFs) involved in inflammation-related genes in humans and mice. Our analysis revealed a set of common TFs, with a primary focus on NF-κB and JUN (Supplementary Fig. 4a and Supplementary Table S4). NF-κB and JUN are well-established regulators of inflammation;^33,34^ however, the role of PRDX4 on their activation remains unexplored. Upon CS (50 μg/cm²) stimulation, MH-S, PBMC-m, and AMs exhibited a marked enhancement in p65 (NF-κB) nuclear translocation, whereas c-jun (JUN) nuclear translocation was not significantly altered (Fig. 3a). *Prdx4* knockdown significantly impeded p65 nuclear translocation (Fig. 3a, yellow dashed line), while c-jun nuclear translocation remained unaffected (Fig. 3a, white dashed line), suggesting that PRDX4 plays an essential role in regulating p65 nuclear translocation. Since PRDX4 cannot directly activate p65, we hypothesize that it may facilitate p65 nuclear translocation by modulating kinase activity. Supporting this hypothesis, pathway enrichment analysis identified the PI3K-AKT pathway as a key regulator of inflammation-related genes. (Supplementary Fig. 4b and Supplementary Table S5). This aligns with previous studies showing that PI3K inhibition in vivo significantly reduces collagen accumulation in murine lungs.^12,13^ Further, PRDX4 knockdown using si_PRDX4 or inhibition of its protein activity with Conoidin A (Con A),^35^ we observed significant downregulation of p-AKT (in the cytoplasm) and p-p65 (in the nucleus) in both PBMC-m and AMs. In contrast, p-PI3K and c-jun levels remained unchanged. (Fig. 3b). These findings suggest that PRDX4 can trigger NF-κB phosphorylation and nuclear translocation via p-AKT (Thr308), but the process is not entirely dependent on PI3K.

Furthermore, inhibiting AKT activity with MK-2206 led to significant downregulation of p-p65 and a concomitant reduction in PRDX4 expression (Fig. 3c). To determine whether AKT was the primary downstream kinase regulated by PRDX4, we utilized myrAKT^Δ4^^−^^129^ to induce constitutive activation of AKT^36^ in AMs and PBMC-m cells (Supplementary Fig. 4c). Our findings indicate that constitutive AKT activation does not affect the regulation of p65 or the inflammatory cytokine secretion by AMs, regardless of PRDX4 knockdown or inhibition (Fig. 3d and Supplementary Fig. 4d). These findings imply that the AKT/NF-κB signaling pathway is the predominant pathway involved in PRDX4-mediated modulation of AM activation and inflammatory cytokine secretion. (Fig. 3e). Similarly, *Prdx4* knockdown in AMs from murine lung tissue also suppressed p65 nuclear translocation and AKT protein expression (Fig. 3f, g). In summary, the AKT/NF-κB pathway is the central signaling pathway mediating the functions of PRDX4.

### PRDX4 oligomer inhibits the formation of PTEN homodimers

In both the CS-stimulated AM system (Fig. 3b, c) and the constitutively active AKT system (Fig. 3d), PRDX4 inhibition had no effect on p-PI3K levels. A lingering question is how PRDX4, which lacks kinase activity, mediates AKT phosphorylation? Beyond PI3K, the phosphatase and tensin homolog (PTEN) also regulates the phosphorylation of the AKT protein at the Thr308 residue. PTEN Homodimers (formed via disulfide bonds between monomers) can localize to the plasma membrane and suppress AKT (Thr308) activity by converting phosphatidylinositol-3,4,5-trisphosphate (PIP3) to phosphatidylinositol-3,4-bisphosphate (PIP2).^18^

**Fig. 4:**
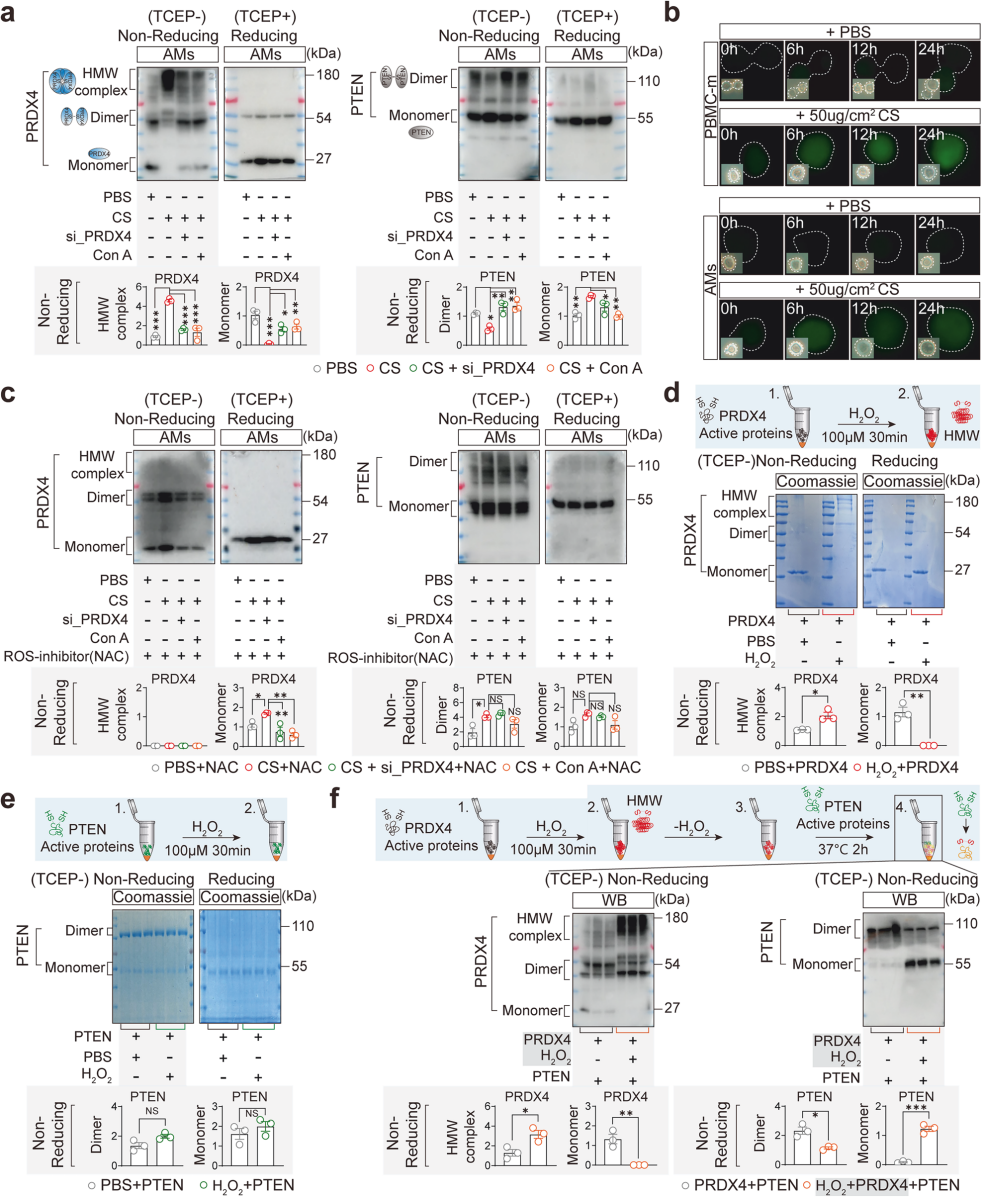
PRDX4 Inhibits the Dimerization of PTEN to Activate the AKT/NF-κB Pathway. **a** Protein expression of PRDX4 (monomer, dimer, oligomers) and PTEN (monomer, dimer) in AMs was assessed following si_PRDX4 treatment and Con A (10 μM) stimulation post-CS (50 μg/cm²) exposure. Non-Reducing and Reducing SDS-PAGE analyses were conducted to detect protein expression. 20 mM NEM was used for alkylation to block the free sulfhydryl group in all cell lysates. Then non-Reducing SDS-PAGE was performed without thiol-reducing agents, with protein denaturation at 70 °C for 10 min. For Reducing SDS-PAGE, TCEP (5 mM) was included and incubated for 30 min prior to protein denaturation at 100 °C for 10 min. Further experimental details are provided in the “Materials and methods” section. **b** After a 24-h CS (50 μg/cm²) exposure, fluorescence probe DCFH-DA was used to test ROS (green) levels in PBMC-m and AMs. A high-magnification view of the selected area is depicted in the right panel. **c** In the presence of NAC (2 μM), PBMC-m and AMs were stimulated with CS (50 μg/cm²) and treated with si_PRDX4 and Con A (10 μM) for 48 h. Non-Reducing and Reducing SDS-PAGE were utilized to assess the expression of PRDX4 (monomer, dimer, HMW oligomer) and PTEN (monomer, dimer) proteins. A bar graph presents the quantitative analysis of PRDX4 and PTEN protein expression, with each experiment conducted three times. Recombinant active PRDX4 (**d**) and PTEN (**e**) proteins (1 μg each) were treated with H_2_O_2_ (100 μM) for 30 min and then analyzed under Reducing and Non-Reducing conditions using Coomassie Blue staining. A bar graph displays the quantitative analysis of PRDX4 and PTEN protein expression, with each experiment repeated three times. **f** Following a 30-min H_2_O_2_ treatment (100 μM), 1 μg of recombinant active PRDX4 protein was incubated without H_2_O_2_, then 1 μg of recombinant active PTEN protein was added and incubated at 37 °C for 2 h. Non-Reducing SDS-PAGE was used to detect the expression of PRDX4 (monomer, dimer, HMW oligomer) and PTEN (monomer, dimer) proteins. A bar graph presents the quantitative analysis of PRDX4 and PTEN proteins, with each experiment repeated three times. Scale bars: 5 µm (**b**). Data are presented as mean ± standard error of the mean (Mean ± SEM). Statistical significance was determined using two-tailed unpaired t-tests (**d**, **e**, **f**) and One-Way ANOVA (**a**, **c**) for all experiments. *P ≤ 0.05; **P ≤ 0.01; ***P ≤ 0.001

To avoid interference of free sulfhydryl-disulfide bond exchange or sulfhydryl oxidation in proteins in the experiments, we first blocked the free sulfhydryl groups by treating the cell extracts with N-Ethylmaleimide (NEM).^23^ Upon non-reducing polyacrylamide gel electrophoresis (PAGE), we observed an increase in PRDX4 high-molecular-weight (HMW) oligomers and a marked decrease in PTEN dimers in CS-stimulated AMs. Conversely, PRDX4 knockdown or inhibition led to an elevation in PTEN dimers and a reduction in monomers (Fig. 4a). Nevertheless, upon treatment with the thiol-reducing agent TCEP, which is specific for cleaving disulfide bonds, PRDX4 and PTEN were exclusively detected as monomers in reducing PAGE (Fig. 4a). This indicates that disulfide bonds are crucial for the assembly of PRDX4 HMW oligomers and PTEN dimers, while PRDX4 oligomerization potentially serves as a key factor in the disruption of PTEN dimerization. Previous studies have reported that PRDX4 is primarily localized in the endoplasmic reticulum (ER),^23^ while PTEN homodimers must anchor to the plasma membrane to inhibit AKT activity.^18^ To investigate the subcellular site of PRDX4-PTEN interaction, we examined the distribution of PRDX4 and PTEN in the ER of lung macrophages from CS-exposed (silicosis) mice. The results showed that PRDX4 and PTEN were present not only in the endoplasmic reticulum of pulmonary macrophages in silicosis mice (yellow dot) but also in the cytoplasm (Supplementary Fig. 4e, f). Further, we fractionated ER and ER-depleted cytosolic components from CS-stimulated alveolar macrophages (AMs). Under CS exposure, PRDX4 oligomer levels increased in the ER, while PTEN dimers decreased and monomers increased (Supplementary Fig. 5a). In the cytosol (ER-depleted), PRDX4 oligomers were also elevated, and PTEN dimers were reduced; however, PTEN monomers were also decreased (Supplementary Fig. 5b). These findings suggest that PRDX4 oligomers and PTEN co-localize in both the ER and cytosol, where they may interact and subsequently influence PTEN dimer formation. The mechanism driving PRDX4 oligomer formation at room temperature in CS-stimulated AMs remains unclear. CS is a water- and acid-insoluble solid that lacks protein-oxidizing capabilities. However, upon CS-induced macrophage phagocytosis, aberrant mitochondrial metabolism can result in excessive accumulation of reactive oxygen species (ROS).^37^ Likewise, after 24-h in vitro exposure to CS at a concentration of 50 μg/cm², both PBMC-m and AMs demonstrated a significant increase in ROS (Fig. 4b). ROS, particularly hydrogen peroxide (H_2_O_2_), exhibit strong oxidizing properties and can reversibly oxidize the oxidation-sensitive cysteine (Cys) residues (–SH) on target proteins, thereby modulating intracellular signaling and function.^38^ Supporting this, treatment with the ROS clearance agent N-acetylcysteine (NAC) effectively suppressed PRDX4 oligomer formation in CS-stimulated AMs, without affecting the formation of PTEN dimers (Fig. 4c). This suggests that ROS-mediated oxidation plays a crucial role in PRDX4 oligomerization. Considering that in bleomycin (BLM)-induced idiopathic pulmonary fibrosis (IPF), BLM can also induce oxidative stress in activated AMs,^39^ we stimulated AMs with BLM (1 µg/ml) for 12 h and 24 h, and observed an increase in cellular ROS levels (Supplementary Fig. 5c). Meanwhile, non-reducing Western blot analysis revealed elevated PRDX4 oligomers and reduced PTEN dimers in AMs (Supplementary Fig. 5d). These findings indicate that the changes in PRDX4 and PTEN induced by BLM are similar to those observed under CS stimulation. In contrast, when MLE12 and A549 cells were stimulated with CS (50 µg/cm²) for 48 h, no PRDX4 oligomer formation was detected; only monomers and dimers were observed (Supplementary Fig. 5e). This suggests that PRDX4 oligomer formation under CS stimulation may be a macrophage-specific phenomenon.

To determine whether PRDX4 oligomers can directly inhibit PTEN dimer formation, we established a purified protein reaction system under NEM-free conditions, ensuring the preservation of sulfhydryl groups necessary for PRDX4 oligomerization and PTEN dimerization. Upon H₂O₂ treatment, recombinant active PRDX4 oligomer formation exhibited a marked increase under non-reducing conditions, with a corresponding reduction in monomeric forms (Fig. 4d and Supplementary Fig. 5f, g). In contrast, the levels of PTEN dimers and monomers remained unaffected upon H₂O₂ treatment (Fig. 4e). This suggests that H_2_O_2_ can induce the oxidation of PRDX4, leading to oligomer formation, but does not significantly affect PTEN dimerization (Fig. 4e). Furthermore, introducing recombinant active PTEN protein to the reaction mixture after PRDX4 oxidation demonstrated that PRDX4 oligomers effectively inhibit PTEN dimer formation (Fig. 4f). This finding elucidates the direct inhibitory effect of PRDX4 oligomers on PTEN homodimer formation, an essential mechanism in AKT activation. Additionally, since Conoidin A was a PRDX4 protease inhibitor that can covalently bind to the catalytically active cysteine (Cys) residues on PRDXs, thereby irreversibly inhibiting its activity.^35^ Our results in the current reaction system indicated that after oxidation of the active PRDX4 protein by H_2_O_2_, Conoidin A did not influence the formation of PRDX4 oligomers. (Supplementary Fig. 5h).

### PRDX4 oligomers induce PTEN dimer formation via oxidation at Cys124 and Cys245

**Fig. 5:**
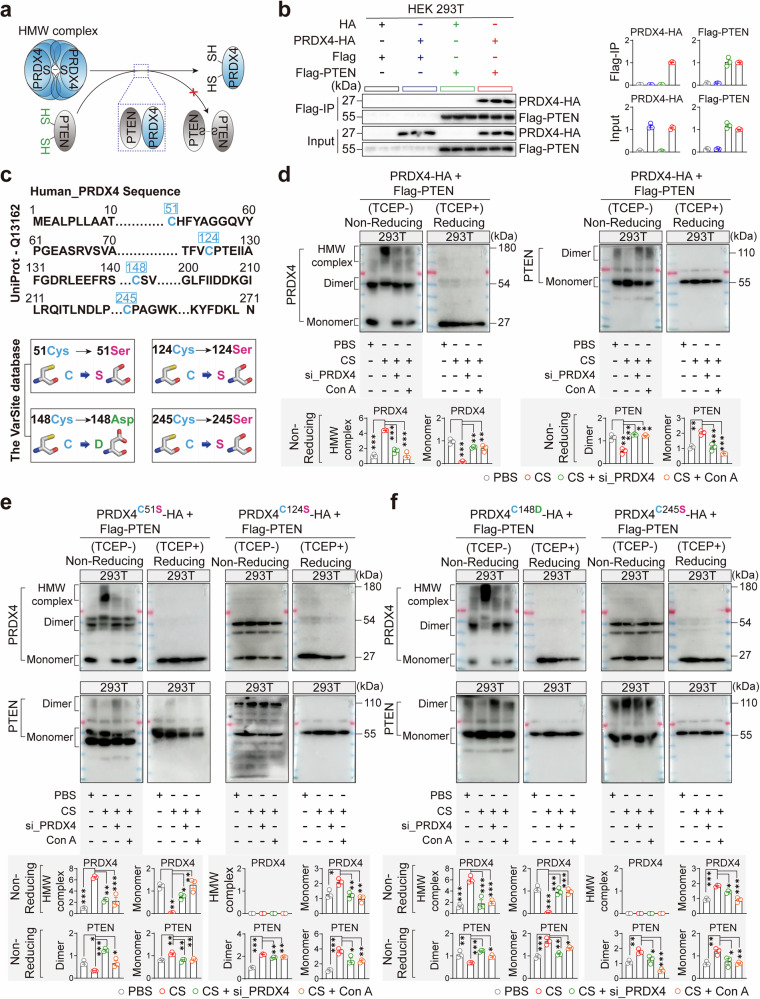
Cys124 and Cys245 within PRDX4 oligomers influence the formation of PTEN dimers. **a** A schematic illustration depicts the interaction between PRDX4 and oxidized PTEN, leading to the formation of endogenous disulfide bonds. **b** Co-Immunoprecipitation (Co-IP) analysis was conducted to examine the protein complexes of PRDX4-HA and PTEN-Flag in HEK293T cells transfected with overexpression plasmids pcDNA3.1-H_PRDX4-3×HA-EF1-ZsGreen1 (PRDX4-HA) and pcDNA3.1-H_PTEN-3×Flag-EF1-ZsGreen1 (PTEN-Flag). The right column presents the quantitative analysis of PRDX4 and PTEN protein expression, with each experiment repeated three times. **c** The PRDX4 protein sequence and optimal mutation sites for the four cysteine residues (Cys51, Cys124, Cys148, and Cys245) are detailed. The full-length amino acid sequence (271aa) of PRDX4 is sourced from the UniProt database (accession number: Q13162), with the blue box highlighting the cysteine positions. The bottom section shows optimal mutation sites predicted by the Varsite database after simulating mutations to minimize structural and functional impact. **d** Non-Reducing and Reducing SDS-PAGE analyses were performed to assess the expression of PRDX4 (monomer, dimer, oligomers) and PTEN (monomer, dimer) proteins in HEK293T cells treated with si_PRDX4 and Con A (10 μM) following CS (50 μg/cm²) stimulation. The bottom column graph displays the quantitative analysis results of PRDX4 and PTEN proteins, with each experiment repeated three times. **e** Non-Reducing and Reducing SDS-PAGE analyses were conducted on PRDX4 (monomer, dimer, oligomers) and PTEN (monomer, dimer) protein expression in HEK293T cells with Cys51 mutated to Ser51 (C51S) and Cys124 mutated to Ser124 (C124S). The bottom column graph presents the quantitative analysis results of PRDX4 and PTEN proteins, with each experiment repeated three times. **f** Non-Reducing and Reducing SDS-PAGE analyses were conducted on PRDX4 (monomer, dimer, oligomers) and PTEN (monomer, dimer) protein expression in HEK293T cells with Cys148 mutated to Asp148 (C148D) and Cys245 mutated to Ser245 (C245S). The bottom column graph presents the quantitative analysis results of PRDX4 and PTEN proteins, with each experiment repeated three times. Data are presented as mean ± standard error of the mean (Mean ± SEM). Significance was determined by One-Way ANOVA for all experiments (**d**, **e**, **f**). Non-Reducing SDS-PAGE was performed without thiol-reducing reagents, with protein denaturation at 70 °C for 10 min (**d**, **e**, **f**). For Reducing SDS-PAGE, TCEP (5 mM) was added, incubated for 30 min, and then heated at 100 °C for 10 min (**d**, **e**, **f**). NS not significant; *P ≤ 0.05; **P ≤ 0.01; ***P ≤ 0.001

The assembly and disassembly of HMW oligomers exist in a dynamic equilibrium. Once the disulfide bonds within PRDX4 oligomers are formed, the thiolate (–SH) groups of PTEN monomers could attack the disulfide bond of PRDX4, thus inhibiting the formation of PTEN dimers (Fig. 5a).

In addition to PTEN, many cytosolic phosphatases and signaling proteins contain active cysteine residues; we need to determine whether PRDX4 oligomers modulate AM activation by specifically regulating PTEN or by nonspecifically oxidizing other thiol-dependent regulatory factors. We overexpressed PTEN in murine macrophage cell lines (Raw264.7 and MH-S) (Supplementary Fig. 6a). The results showed that under CS stimulation, PTEN overexpression markedly reduced the phosphorylation levels of p-AKT (Thr308) and p-P65 (Supplementary Fig. 6b–d), indicating that PTEN is likely the primary effector protein through which PRDX4 regulates the AKT/p65 pathway.

To further investigate the mechanism of PRDX4-mediated PTEN deactivation, we transfected HEK293T cells with plasmids encoding PRDX4-HA and PTEN-Flag and conducted co-immunoprecipitation (Co-IP) assays on the lysates, suggesting that PRDX4 directly modulates PTEN activity through physical interaction (Fig. 5b). Disulfide bonds (S–S) primarily arise from the oxidation of side-chain thiol groups (–SH) of cysteine (Cys) residues.^40^ Based on the principles of amino acid site mutations, we introduced site-specific mutations at the four cysteine residues (51, 124, 148, 245) in the monomeric form of PRDX4.^41^ Our findings indicated that the most effective mutations were Cys51, Cys124, and Cys245 to serine (Ser, S), while Cys148 was optimally mutated to aspartic acid (Asp, D) (Fig. 5c). HEK293T cells were transfected with wild-type (WT) PRDX4-HA and PTEN-Flag overexpression plasmids and analyzed using non-reducing SDS-PAGE, we observed an increase in HMW PRDX4 oligomers and a significant reduction in PTEN dimers upon CS induction. In contrast, PRDX4 knockdown or inhibition led to elevated levels of PTEN dimers (Fig. 5d). When treated with TCEP, both PRDX4 and PTEN appeared exclusively in their monomeric forms. These results align with the observed alteration on the different forms of endogenous PRDX4 and PTEN proteins in 293T cells (Supplementary Fig. 6e, f). Additionally, mutations in the non-catalytic cysteine residues Cys51 and Cys148 of PRDX4 did not significantly affect the formation of PRDX4 oligomers or PTEN dimers (Fig. 5e, f, left). In contrast, mutations of the catalytic cysteine residues Cys124 and Cys245 in PRDX4 led to a marked decrease in PRDX4 oligomers and a corresponding increase in PTEN dimers, while PTEN monomer levels decreased (Fig. 5e, f, right). This suggests that the two catalytic cysteine residues, Cys124 and Cys245, are essential for maintaining the oligomeric state of PRDX4 and for preventing PTEN dimer formation (Supplementary Fig. 6g).

### Inhibition of PRDX4 activity in vivo significantly ameliorates silicosis-induced fibrosis

**Fig. 6:**
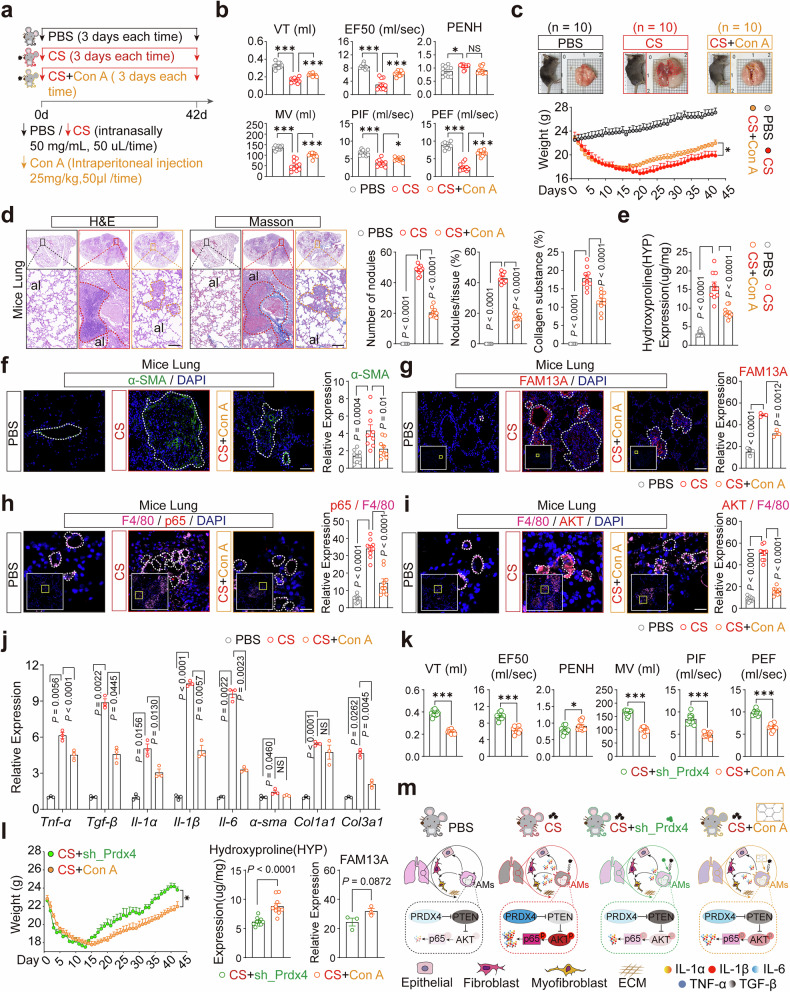
Inhibition of PRDX4 activity in vivo significantly ameliorates silicosis-induced fibrosis. **a** A schematic diagram illustrates the construction process of the mouse model, wherein phosphate-buffered saline PBS and CS were administered via nasal instillation post-anesthesia. PBS (50 μL/time) and CS (50 mg, 50 μL/time) were delivered every three days for a period of 42 days. Conoidin A (Con A, 5 mg/kg) was injected intraperitoneally every three days at a volume of 50 μL. CS, a non-toxic and odorless white powder with particle sizes of 5–10 μm, was suspended in PBS to create a milky solution. Con A was prepared as a clear solution containing 10% DMSO, 40% PEG300, 5% Tween-80, and 45% saline. **b** Lung function data were collected for three groups of mice: PBS (n = 10), CS (n = 10), and CS+Con A (n = 10). Measurements included tidal volume (TV), expiratory flow rate at 50% of tidal volume (EF50), enhanced pause (PENH), minute ventilation (MV), peak inspiratory flow (PIF), and peak expiratory flow (PEF). Each data point corresponds to an individual mouse. **c** The lung appearance and body weight change line graph for the three groups of mice are presented. Red and yellow dashed boxes highlight the regions of lung tissue damage. **d** Histological analysis of lung tissue from three groups of mice was conducted using H&E and Masson staining. High-power magnification images of selected areas are presented in the inset, with dashed lines highlighting regions of lung nodules and collagen deposition. The right column displays the statistical analysis of nodule count and collagen content in lung tissue, where each data point corresponds to an individual mouse. **e** The hydroxyproline (HYP) content in lung tissue was measured across the three groups of mice, with each data point representing an individual mouse. **f** IF analysis of α-SMA in lung tissue from the three groups of mice is shown. The right column presents the statistical analysis of α-SMA expression. For this analysis, three mice were selected for IF staining, and three fields of view were examined per mouse, with each data point representing a single field of view. **g** IF analysis of FAM13A in lung tissue from the three groups of mice is depicted. The right column presents the statistical analysis of FAM13A expression, with each data point representing an individual mouse. IF analysis of p65 (**h**) and AKT (**i**) in AMs from the lung tissue of three groups of mice is depicted. High-power magnification images of AMs, delineated by yellow solid lines, are shown in the inset. White dashed lines outline the macrophages, and red arrows indicate p65 expression. The right column presents the relative expression ratio of p65 and AKT proteins in F4/80-positive macrophages. For this analysis, three mice were selected for IF staining, with three fields of view analyzed per mouse, each data point representing a single field of view. **j** Expression levels of inflammatory genes (*Tnf-α, Il-1α, Il-1β, Il-6*) and fibrotic genes (*Tgf-β, α-sma, col1a1, col3a1*) in lung tissue were measured across the three groups of mice, with each data point representing an individual mouse. The experiment was conducted three times. **k, l** Changes in body weight, HYP content, FAM13A protein expression, and lung function were assessed in mice following AAV-mediated knockdown of PRDX4 (CS+sh_Prdx4) and Con A blockade (CS+Con A). **m** A schematic diagram illustrates how PRDX4 regulates the PTEN/AKT/NF-κB pathway to activate AMs and advance the progression of silicosis-associated fibrosis. Scale bars: 20 µm (**h,** **i**), 50 µm (**f,** **g**), 100 µm (**d**), 1000 µm (**c**). Abbreviations: al, alveoli; br, bronchi. Data are presented as mean ± standard error of the mean (Mean ± SEM). Statistical significance was determined using two-tailed unpaired t-tests (**c,** **k,** **l**), One-Way ANOVA (**b,** **d,** **e,** **f,** **g,** **h,** **i**), and Two-Way ANOVA (**j**). NS, not significant; *P ≤ 0.05; **P ≤ 0.01; ***P ≤ 0.001

Previous studies have reported that in the CS-induced silicosis mouse model, the disease can be staged into the inflammatory phase, progressive phase, and fibrotic phase at 21, 42, and 63 days, respectively.^5^ To evaluate the therapeutic efficacy of targeting PRDX4 at different stages of silicosis, we administered Conoidin A (Con A) to CS-exposed mice for 21, 42, and 63 days (Fig. 6a and Supplementary Fig. 7a, f). Lung function data revealed that, compared to the CS-only group, mice treated with CS and Conoidin A (CS+Con A) exhibited improvements in tidal volume (TV), expiratory flow rate at 50% volume (EF50), minute ventilation (MV), peak inspiratory flow (PIF), and peak expiratory flow (PEF) (Fig. 6b and Supplementary Fig. 7b, g). These indicated that inhibiting PRDX4 protein activity in vivo could enhance lung function. Similarly, Lung morphology and weight data indicated that the extent of lung damage in the CS+Con A group was less than that in the CS group, also the mouse weight displayed an upward trend (Fig. 6c and Supplementary Fig. 7c, h). Pathological examination showed a marked reduction in lung nodule formation and collagen deposition in the CS+Con A group relative to the CS group (Fig. 6d and Supplementary Fig. 7d, i). Similarly, hydroxyproline (HYP) levels were markedly lower in the CS+Con A group than in the CS group (Fig. 6e and Supplementary Fig. 7e, j). This result shows that targeted inhibition of PRDX4 was effective against all stages of silicosis in mice. These results indicate that targeted inhibition of PRDX4 exerts therapeutic effects in silicosis mice across different disease stages.

Given the progressive and irreversible nature of pulmonary fibrosis, to assess the role of PRDX4 after the establishment of fibrosis in silicosis, we initiated Conoidin A treatment at day 42 post-CS exposure (fibrosis formation stage) and evaluated the therapeutic effects on day 63 (Supplementary Fig. 7k). The results showed that Conoidin A treatment still partially improved lung function and suppressed the progression of pulmonary inflammation and fibrosis (Supplementary Fig. 7l–o). We speculate that this may be related to the persistent functional importance of AMs in the late fibrotic stage of silicosis. Consistently, single-cell sequencing analysis revealed that at the fibrotic stage of silicosis (day 56), AMs were highly infiltrated and exhibited pro-fibrotic functions.^42^

Similarly, α-SMA expression (green arrows), and FAM13A protein levels were markedly lower in the CS+Con A group than in the CS-only group (Fig. 6f, g). Furthermore, in vivo blockade of PRDX4 could prevent the nuclear translocation of p65 and suppress the expression of AKT protein in alveolar macrophages (F4/80) (Fig. 6h, i). This inhibition also downregulated inflammatory markers (*Tnf-α, l-1α, Il-1β, Il-6*) and fibrosis-related genes (*Tgf-β, Col1a1, Col3a1*) in the lung tissues (Fig. 6j). Notably, these effects were consistent with the changes observed in the CS+sh_Prdx4 group of mice (Fig. 2e and Fig. 3f, g). These results suggest that both knockdown and blockade of PRDX4 can inhibit the AKT/NF-κB pathway in AMs in vivo, thereby reducing lung inflammation and fibrosis.

When comparing weight recovery, hydroxyproline (HYP) levels, FAM13A expression, and lung function between the AAV-mediated PRDX4 knockdown (CS+sh_PRDX4) and Con A-inhibited (CS+Con A) groups, distinct differences emerged. Although the onset of weight recovery was delayed in the CS+sh_PRDX4 group compared to the CS+Con A group, the overall rate of weight recovery in the CS+sh_PRDX4 group was significantly higher (Fig. 6k). Furthermore, assessments of HYP content, FAM13A expression, and lung function demonstrated that AAV-mediated PRDX4 knockdown was more effective than Con A inhibition in mitigating silicosis in mice (Fig. 6k, l). In summary, these findings establish PRDX4 as a critical target for modulating inflammation and fibrosis in silicosis. Both genetic knockdown and pharmacological inhibition of PRDX4 effectively suppress PTEN/AKT/NF-κB pathway activation in alveolar macrophages, thereby alleviating disease progression (Fig. 6m).

## Discussion

Our study provides novel insights into the dual role of PRDX4 in oxidative stress and inflammation, particularly in the context of silicosis. While PRDX4 is known to function as an antioxidant by detoxifying H₂O₂, our findings suggest that in AMs exposed to crystalline silica (CS), PRDX4 plays a pathological role by facilitating PTEN oxidation, thereby activating the AKT/NF-κB signaling pathway. This activation promotes AM-driven inflammation and fibrosis, exacerbating disease progression.

AMs are pivotal immune cells that safeguard the pulmonary systems of both humans and mice. They ingest inhaled bacteria within the alveolar cavities and interstitial spaces, thus preserving lung homeostasis.^43^ However, upon encountering free crystalline silica (CS) particles in the alveolar cavity, AMs engulf these particles, initiating a cascade that enhances metabolic activity and ROS production, triggering oxidative stress and localized inflammation.^44,45^ In turn, activated macrophages can intensify lung inflammation and fibrosis by attracting inflammatory cells and promoting epithelial cell differentiation.^6^

PRDX4 is the only peroxiredoxin family member localized to the endoplasmic reticulum (ER), where it detoxifies hydrogen peroxide (H₂O₂) and regulates protein disulfide isomerase (PDI) activity.^40^ While PRDX family proteins are generally considered protective against oxidative stress-induced inflammation, emerging evidence suggests they can also act as pro-inflammatory mediators. Research indicates that oxidative stress triggers inflammatory responses, which PRDXs can mitigate by reducing oxidative stress.^46^ However, some studies have demonstrated that PRDXs can also serve as danger signals, instigating robust inflammatory reactions in brain injury models.^47^ Similarly, PRDX4 has been reported to oxidize Caspase-1 in macrophages, inhibiting inflammasome activation and IL-1β production.^48^ Conversely, other studies have found that elevated PRDX4 expression in the lungs of mice with idiopathic pulmonary fibrosis (IPF) intensifies bleomycin-induced lung inflammation and fibrosis, although the underlying mechanism is not well understood; additionally, a positive correlation exists between elevated PRDX4 serum levels in IPF patients and disease progression.^20^ These discrepancies underscore the complexity of PRDX4’s role in inflammation, suggesting a context-dependent function that needs further investigation.

Our research indicates that PRDX4 is significantly upregulated in the AMs of both silicosis patients and mice. In vitro suppression of PRDX4 markedly reduced the inflammatory response of primary human AMs, while in vivo PRDX4 knockdown in AMs mitigated lung inflammation and fibrosis, improving lung function in a silicosis mouse model. We hypothesize that elevated PRDX4 levels in the lungs may trigger inflammatory responses in AMs, potentially outweighing any anti-inflammatory benefits.

The PI3K/AKT pathway plays a central role in macrophage activation and inflammatory cytokine secretion. PI3K, a heterodimer consisting of regulatory (p85) and catalytic (p110) subunits, is activated upon recruitment to receptor complexes, leading to the phosphorylation of PIP2 to PIP3 on the plasma membrane. This process facilitates the activation of AKT through its phosphorylation at Thr308 by 3-phosphoinositide-dependent kinase 1 (PDK1). AKT, in turn, regulates multiple transcription factors involved in immune responses.^49^ For example, AKT phosphorylation at Thr308 and NF-κB activation are known to drive pulmonary hypertension under hypoxic conditions.^50^ Our findings reveal that PRDX4 enhances AKT phosphorylation at Thr308, thereby promoting NF-κB activation and nuclear translocation, ultimately amplifying inflammation and fibrosis. Interestingly, PRDX4 appears to upregulate its own expression, albeit independently of PI3K, suggesting a feedback loop that sustains AM activation.

PTEN, a critical negative regulator of AKT, dephosphorylates PIP3 to PIP2, thereby suppressing AKT activation.^51,52^ This function relies on PTEN homodimerization, which occurs through disulfide bond formation between cysteine residues in the C2 domain.^53,54^ In normal cells, homodimeric PRDX4 facilitates disulfide bond formation in protein disulfide isomerase (PDI) enzymes within the ER.^55^ However, our results show that under CS stimulation, PRDX4 oligomers might react with PTEN both in the ER and the cytosol. Moreover, PRDX4’s role in oxidative modifications of target proteins in CS-activated macrophages remains unclear. Recent studies have indicated that PRDX4 homodimers, induced by H_2_O_2_, can directly bind to and oxidize GDE2, leading to the formation of endogenous disulfide bonds. This process inhibits both the membrane trafficking and neuromodulatory functions of GDE2.^23^ Oxidized HMW super-oligomers of PRDX4 can facilitate protein folding and disulfide bond formation.^22^ Our study revealed that PRDX4 predominantly occurs in monomeric and dimeric forms within normal macrophages. In contrast, macrophages activated by CS undergo a transition from these forms to HMW super-oligomers. These super-oligomers of PRDX4 can bind and oxidize monomeric PTEN, thereby preventing the formation of PTEN homodimers. Notably, Cys124 and Cys245 are pivotal cysteine residues that facilitate the assembly of super-oligomeric PRDX4. Our findings suggest that the super-oligomeric form of PRDX4 may be a critical protein in the pathogenesis of silicosis and fibrosis.

Given the lack of effective treatments for silicosis, we explored PRDX4 inhibition as a therapeutic strategy. We selected Conoidin A (Con A), a PRDX4 inhibitor with demonstrated anti-inflammatory and antifibrotic effects, and compared its efficacy with AAV-mediated PRDX4 knockdown in a silicosis mouse model. While Con A significantly improved lung function and reduced fibrosis, AAV-mediated PRDX4 knockdown exhibited superior therapeutic effects, suggesting that genetic suppression of PRDX4 may provide a more robust approach. Compared to previously reported multi-target drug therapies for silicosis, our findings highlight the potential of a single-target inhibition strategy.^56^

In summary, our study identifies PRDX4 as a key regulator of AM-driven inflammation and fibrosis in silicosis. By preventing PTEN homodimerization, PRDX4 sustains AKT/NF-κB activation, thereby exacerbating lung injury. These findings not only provide new mechanistic insights into PRDX4-mediated redox signaling but also establish PRDX4 as a promising therapeutic target for fibrosis-related lung diseases. While our model was derived from AM studies, given PTEN’s critical role in tumor suppression, these findings may have broader implications for diseases characterized by PRDX4 and PTEN dysregulation.

## Materials and methods

### Collection and retrieval of lung tissue sequencing data

In this study, whole-genome sequencing data from lung tissues of seven healthy individuals and ten patients with silicosis (HRA000560)^24^ were obtained from the Genome Sequence Archive (GSA) database (https://ngdc.cncb.ac.cn/gsa-human/). The sequencing data sources for various stages of silicosis in mouse lung tissues have been previously reported.^57^ A search of the Gene Set Enrichment Analysis (GSEA) database (http://www.gsea-msigdb.org/) using the keywords “silicosis-associated inflammation and fibrosis genes” identified 201 inflammation-related and 307 fibrosis-related genes. The sequencing data for silicosis patients and mouse lung tissues, along with the key gene lists, are detailed in Supplementary Table S1. In addition, RNA-sequencing data of BALF samples from 176 patients with IPF,^58^ along with their corresponding prognostic information, were downloaded from the GEO database (http://www.gsea-msigdb.org/) (Supplementary Table S6).

### Acquisition of human lung tissue and BALF samples

Lung tissue samples from silicosis patients and controls were collected between January 2016 and April 2023 at the Thoracic Surgery Department of the Affiliated Tumor Hospital of Anhui University of Science and Technology (Huainan Oriental Cancer Hospital). The silicosis group comprised three males, while the chronic obstructive pulmonary disease (COPD) group included one female and two males. Bronchoalveolar lavage fluid (BALF) samples were obtained from six male normal miners and ten male silicosis patients between January 2019 and June 2023 at the Huainan Huaihe Energy Group Occupational Disease Prevention and Control Hospital. At the same time, lung function data were collected from 10 male patients with silicosis. During sample collection, each collection provided 100–200 ml of fluid. Lung tissues were rapidly frozen in liquid nitrogen and stored at −80 °C. BALF samples were filtered through sterile gauze, centrifuged, and stored at −80 °C. The use of patient samples in this study was approved by the Ethics Review Committee of Anhui University of Science and Technology (Approval No. HX-001). All participants provided written informed consent, and the study adhered to the principles of the Helsinki Declaration.

### Immunofluorescence staining of tissues and cells

Immunofluorescence staining was conducted as previously described.^59^ In brief, human and murine lung tissues were fixed with 10% formaldehyde, embedded in paraffin, and sectioned into 5 µm slices. After dehydration, antigen retrieval was performed by boiling the sections in EDTA antigen retrieval solution (Servicebio, Cat. No. G1207) at pH 8.0 for 8 min. Sections were outlined with immunohistochemistry pens (Servicebio, Cat. No. G6100) to prevent antibody loss. Following a 30-min block with BSA (Servicebio, Cat. No. GC305010), primary antibodies were applied and incubated overnight at 4 °C, followed by a 1-h incubation with corresponding secondary antibodies at room temperature. Cell nuclei were counterstained with DAPI (Servicebio, Cat. No. G1012) for 10 min in the dark at room temperature. Anti-fluorescence quenching reagent (Servicebio, Cat. No. G1221) was applied for 5 min at room temperature before observation and imaging with a fluorescence microscope (Leica, DMI3000B). Image analysis was performed using ImageJ software (V1.8.0). For cellular immunofluorescence, cells were fixed with 4% formaldehyde for 30 min at room temperature, permeabilized with 0.5% Triton X-100 (Beyotime, Cat. No. P0096), and then incubated with primary and secondary antibodies. The following antibodies were used: PRDX4 (1:200, ab59542, Abcam), PTEN (1:200, A11193, Abclonal), KDEI (1:200, ab176333, Abcam), CD68 (1:500, GB14043, Servicebio), CD80 (1:200, A16039, ABclonal), CD206 (1:500, GB113497, Servicebio), F4/80 (1:1000, GB113373, Servicebio), α-SMA (1:200, ab5694, Abcam), FAM13A (1:200, 55401-1-AP, Proteintech), CD3 (1:100, GB13014, Servicebio), CD16 (1:200, A23541, ABclonal), LY6G (1:400, GB11229, Servicebio), Phospho-NF-kB p65 (1:200, AP0123, ABclonal), c-Jun (1:200, GB11515, Servicebio), Phospho-Akt (Thr308) (1:200, 13038T, CST), and secondary antibodies including HRP-conjugated Goat Anti-Rabbit IgG (1:500, GB23303, Servicebio), FITC-labeled Goat Anti-Rabbit IgG (1:500, GB22303, Servicebio), Cy5-conjugated Goat anti-Rabbit IgG (1:500, GB27303, Servicebio), Cy5 Goat anti-Mouse IgG (1:500, GB27301, Servicebio), Cy3-labeled Goat Anti-Rabbit IgG (1:500, GB21303, Servicebio), Alexa Fluor® 488-conjugated Goat Anti-Mouse IgG (H+L) (1:500, GB25301, Servicebio).

### Mouse pulmonary function testing

Mouse pulmonary function was evaluated using a whole-body plethysmography system (Tow-in, WBP-4M). Mice were acclimated to the testing environment for 30 min before the experiments. Once respiration was stabilized, data collection proceeded for 15 min. The parameters measured included inspiratory time (Ti, seconds), expiratory time (Te, seconds), peak inspiratory flow (PIF, ml/s), peak expiratory flow (PEF, mL/s), respiratory frequency (F, breaths/min), tidal volume (TV, mL), minute ventilation (MV, mL), accumulated volume (AV, mL), expiratory flow at 50% of expired volume (EF50, mL/s), end-inspiratory pause (EIP), end-expiratory pause (EEP), relaxation time (TR), and enhanced pause (PENH). In this study, PIF, PEF, TV, MV, EF50, and PENH were selected as the primary indices of pulmonary function.^60,61^

### Flow cytometric sorting of lung tissues

For the detection and sorting of cells (Mø, T and NEU) in mouse lung tissues, fluorescent dye-coupled monoclonal antibody (Abs) staining was used for cell surface labeling, including Apc-Cy7 CD45 (BD Pharmingen™, Cat.No 557659, 30-F11, 1/200 dilution), PerCP-Cyanine5.5-CD3e (BD Pharmingen™, Cat.No 551163, 145-2C11, 1/200 dilution), PE-F4/80 (BD Pharmingen™, Cat.No 565410, T45-2342, 1/200 dilution), FITC-CD11b (BD Pharmingen™, Cat.No 557396, M1/70, 1/200 dilution), APC-Ly6G (BD Pharmingen™, Cat.No 560599, 1A8, 1/200 dilution). For the isolation of AMs (alveolar macrophages) and IMs (interstitial macrophages) from lung tissue,^62^ the following antibodies were used: APC-CD64(eBioscience™, Cat.No 17-0641-80, X54-5/7.1, 1/200 dilution), PE-MerTK(eBioscience™, Cat.No 12-5751-80, DS5MMER,1/200 dilution), PerCP-Cyanine5.5-CD11b(eBioscience™, Cat.No 45-0112-80, M1/70, 1/100 dilution), FITC-CD11c(eBioscience™, Cat.No 11-0114-81, N418, 1/100 dilution). All cells were collected and analyzed using BD FACSAria ™ II flow cytometry (BD FACSDiva™ version V8.0) and FlowJo (version V10).

### Cell acquisition and culture

Peripheral blood mononuclear cell-derived macrophages (PBMC-m) utilized in this study were differentiated from peripheral blood mononuclear cells (PBMCs) obtained from healthy volunteers. Approval from the Ethics Committee of Anhui University of Science and Technology (Approval No. HX-001) was secured before collecting 10 ml of venous blood from each participant, which was then diluted in PBS solution at a 1:1 ratio. The diluted blood was carefully layered onto 5 ml of Ficoll-Paque PLUS solution (Cytiva, Cat. No. 17144002) for mononuclear cell separation. After centrifugation at 4 °C (300 × *g*) for 20 min, the mononuclear cell layer was carefully aspirated. The cells were then washed and resuspended in complete RPMI 1640 medium (Thermo Fisher Scientific, Cat. No. 12633012), supplemented with 100 ng/ml macrophage colony-stimulating factor (M-CSF) (MCE, Cat. No. P7050A), and cultured for 14 days to facilitate PBMC differentiation into PBMC-m.^63^ Giemsa staining (Beyotime, Cat. No. P7050A) and CD68 immunofluorescence were employed to evaluate the purity of the PBMC-m cells.

Alveolar macrophages (AMs) were isolated from approximately 200 mL of fresh bronchoalveolar lavage fluid (BALF) derived from healthy miners. The BALF was first filtered through sterile gauze to eliminate large mucus clots, followed by centrifugation at 4 °C at 300 × *g* for 10 min. The cells were then passed through a sterile 70 µm cell strainer (BD Falcon, Cat. No. 352350), washed, and resuspended in high-glucose Dulbecco’s Modified Eagle Medium (DMEM) (Gibco, Cat. No. C11995500BT), supplemented with 100 ng/mL macrophage colony-stimulating factor (M-CSF) to support AMs growth and maintenance. After 7 days, CD68 immunofluorescence was conducted to evaluate the purity of the AMs.

Other cells including mouse alveolar macrophages MH-S (ATCC, CRL-2019), mouse monocyte/macrophage leukemia cell RAW264.7(Pricella, Cat. No. CL-0190), mouse lung epithelial cells MLE12 (ATCC, Cat. No. CRL-2110), human embryonic lung fibroblasts WI-38 (Pricella, Cat. No. CL-0243), and AAV Pro-293T cells (Clontech, Cat. No. 632273) were cultured in high-glucose DMEM medium containing 10% fetal bovine serum (FBS) (Gibco, Cat. No. C0235) and 1% penicillin/streptomycin (P/S) (Beyotime, Cat. No. C0222). Human non-small cell lung cancer cells A549 (Pricella, Cat. No. CL-0016) were cultured in RPMI 1640 complete medium under the same conditions, while human embryonic kidney cells HEK293T (Pricella, Cat. No. CL-0005) were cultured in MEM (Pricella, Cat. No. CM-0001) complete medium. All cells were cultured in a constant temperature incubator (Panasonic) at 37 °C with 5% CO_2_. Cell lines used in this study (except for AMs and PBMC-m cells) were tested for mycoplasma contamination and authenticated by STR profiling.

### Construction and packaging of the AAV vector for gene interference

The interference vector for gene expression, GPAAV-HU6-CMV-eGFP-WPRE, was used as the target gene expression vector, while PHBAAV-RC6 and pHelper served as the packaging plasmids for the AAV9 system. The vector was constructed with a macrophage-specific promoter, SP146 (5-CTAGCGAGGGCGGACCAGAAAAGGAGAAGTAGGAGCCAAGATTTCCAAACTCTGTGGTTGCCTTGCCAAGATTTCCAAACTCTGTGGTTGCCTTGCAGAAAAGGAGAAGTAGGAGAAGCGACTTCCTCTTTCCAGAAGCGACTTCCTCTTTCCAGAGGAAGAGGGCGGAGGCTCACAAGGCAACCACAGAGTTTGGAAATCTTGGAAGCGACTTCCTCTTTCCAGCAGAAAAGGAGAAGTAGGAGAAGCGACTTCCTCTTTCCAGGTCCGCCCTCG-3), and gene sequences targeting PRDX4 based on miR30,^64^ including sh_NC (5-TTCTCCGAACGTGTCACGT-3) and sh_PRDX4 (5- CCACTTGGCC-TGGATTAAT-3). After digestion with Bsu15I (Thermo Fisher Scientific, Cat. No. ER0141) and EcoRI (Thermo Fisher Scientific, Cat. No. ER0271) enzymes, the target gene fragments were inserted into the interference vector using the Hieff CloneTM recombination reaction system. The ligation products were transformed into DH5α (Sangon, Cat. No. B528413) competent cells, and clones were selected for sequencing at a commercial sequencing company (Sangon) to confirm the sequence identity of the inserted fragments with the designed oligo sequences. The resulting clones were confirmed to be the GPAAV-SP146-sh_NC-CMV-eGFP-WPR control and GPAAV-SP146-sh_Prdx4-CMV-eGFP-WPRE interference vectors. AAV Pro-293T cells were employed as the packaging cells for adeno-associated viruses, and the E. coli strain stbl3 was used for amplifying the viral vector and helper packaging plasmids (PHBAAV-RC6 and pHelper). Plasmid extraction kits (Qiagen, Cat. No. 12143) were used to extract the successfully constructed adeno-associated virus recombinant plasmids and packaging plasmids, and their concentration and purity were determined by the UV absorption method to ensure that the A260/A280 ratio of the purified plasmid DNA was between 1.8 and 2.0. The day before transfection, when the confluence of the cells in a 10 cm culture dish reached 60% to 80%, the transfection preparation was initiated. One to two hours before transfection, the cells were switched to fresh serum-free DMEM. The HG transgene reagent (Genomeditech, Cat. No. TG-10012) served as the transfection reagent, and the transfection system consisted of DMEM (850 μL) + interference plasmid (10 μg) + PHBAAV-RC6 (10 μg) + pHelper (10 μg) + HG transgene reagent (120 μg). After transfection for 6 to 8 h, the cells were refed with complete DMEM medium, and the Enhancing buffer (100×, 100 μL) was added to facilitate transfection. Cells and cell supernatants were harvested 72 h post-transfection, subjected to three freeze-thaw cycles, and centrifuged to collect the viral supernatant. The viral supernatant was concentrated by adding Benzonase (50 U/mL) and MgCl_2_ (0.002 M) and incubating at room temperature for 30 min, followed by filtration through a 0.22 μm filter. The virus was purified using iodixanol (Millipore, Cat. No. 1343517) gradients (60%, 40%, 25%, 15%), and collected using Amicon® Ultra 50KD (Millipore, Cat. No. UFC5050) ultrafiltration tubes. The virus titer was determined, and the concentration was above 1.16e + 12 VG/mL. The prepared virus was stored at −80 °C for future use.

### Macrophage-stimulated epithelial-fibroblast differentiation model

The model was divided into two types: macrophage/epithelial (MH-S/MLE12, AMs/A549, and PBMC-m/A549) and macrophage/fibroblast (AMs/WI-38, PBMC-m/WI-38) conversion models. Supernatants from macrophages stimulated with CS were collected by centrifugation at 4 °C, followed by filtration through a 0.45 μm filter. The supernatant was mixed with the culture medium at a 1:1 ratio to culture the epithelial/fibroblast cells. CS (Sigma, No. 637238) was a non-toxic, odorless, white powder of 10–20 nm size, which appeared as a white suspension when dissolved in PBS. It needed to be thoroughly mixed before use and could be directly prepared in the culture medium.

### Animal model construction

Our study exclusively examined male mice. It is unknown whether the findings are relevant for female mice. A total of 130 SPF-grade C57BL/6 male mice aged 8–10 weeks old (22–24 g) were purchased from Henan Scrofula Biotechnology Co., Ltd. All experimental procedures were approved by the Ethics Committee of Anhui University of Science and Technology (University Lun No. GZ-2023-041). The mice were housed in the Experimental Animal Center of the School of Medicine, Anhui University of Science and Technology, under the following conditions: temperature of 20 ± 2 °C, humidity of 55 ± 5%, and a 12-h light-dark cycle. They had ad libitum access to drinking water and were fed three times a week with regular food. Daily weight measurements were recorded. After a 3-day adaptation period, the mice were randomly divided into different groups to construct the animal models.

In the silica-induced lung fibrosis mouse model with PRDX4 knockdown in lung AMs, four experimental groups were established: PBS (n = 10), CS (n = 10), CS+sh_NC (n = 9), and CS+sh_Prdx4 (n = 10). Mice in the PBS and CS groups were administered intranasal PBS (50 μL) or 50 μL CS (50 mg/ml) every three days for 42 days. The CS+sh_NC and CS+sh_Prdx4 groups received a single intratracheal instillation of 50 μl sh_NC/Prdx4 viral suspension (1 × 1011 VG) on day 12. CS (Sigma, Cat. No. S5631), a non-toxic and odorless white powder with particle sizes between 0.5 and 10 μm, formed a milky white suspension when mixed with PBS. Daily monitoring of mouse body weight and survival was conducted, with the exception of one mouse in the CS+sh_NC group that died accidentally during anesthesia. At the conclusion of the 42-day study period, the mice were euthanized, and lung function was evaluated at both the start and finish.

In the Conoidin A (Con A) treatment study of CS-induced silicosis mouse models at the inflammatory phase (day 21), transitional phase (day 42), and fibrotic phase (day 63), the animals were divided into seven groups: PBS group (n = 10), CS-21 group (n = 10), CS-21 + Con A group (n = 10), CS-42 group (n = 10), CS-42 + Con A group (n = 10), CS-63 group (n = 10), and CS-63 + Con A group (n = 10). Mice in the PBS and CS groups received intranasal instillation of 50 μL PBS or 50 μL CS suspension (50 mg/ml) every three days, for a total duration of 21, 42, or 63 days, respectively. Concurrently, Con A (5 mg/kg) was administered via intraperitoneal injection at a volume of 50 μL every three days. Con A was prepared as a clear solution with 10% DMSO, 40% PEG300, 5% Tween-80, and 45% Saline.

In the CS-induced silicosis model in which Con A treatment was initiated after fibrosis formation (day 42), mice were divided into two groups: CS-42 + DMSO-21 group (n = 10) and CS-42 + Con A-21 group (n = 10). All mice received intranasal instillation of 50 μL CS suspension (50 mg/ml) every three days for 42 days, after which CS administration was discontinued. Subsequently, mice in the CS-42 + Con A-21 group were administered Con A (50 μL, 5 mg/kg) via intraperitoneal injection every three days, while the CS-42 + DMSO-21 group received DMSO as a control. Con A administration was stopped after 21 days of treatment (day 63). Daily monitoring of mouse body weight and survival was conducted, and lung function was assessed at the study’s commencement and conclusion. The study adhered to stringent animal welfare principles, including twice-daily evaluations of mouse appetite, activity, behavior, body temperature, and body weight changes. Euthanasia was performed at the study’s end using 1% isoflurane (RWD, Cat. No. S5631) followed by cervical dislocation.

### H&E, Masson staining, Sirius red stain, and HYP testing

As described previously,^59^ tissues were fixed with formalin, dehydrated, and embedded in paraffin for subsequent staining with hematoxylin and eosin (H&E), Masson’s trichrome, and Sirius red stain. For H&E staining, lung tissue sections were deparaffinized and rehydrated using xylene and a graded series of ethanol (100%, 90%, 80%, and 70%). The sections were stained with hematoxylin solution (Beyotime, Cat. No. C0105S) for 3–5 min at room temperature, followed by rinsing in running water for 5 min to remove excess staining. Then, the sections were stained with eosin staining solution for 1 min, followed by two washes with 70% ethanol. After mounting with neutral gum (Solarbio, Cat. No. G8590), the sections were observed and photographed using an optical microscope (OLYMPUS, BX53 + DP74). Cell nuclei appeared blue, while the cytoplasm appeared pink or red. For Masson’s trichrome staining, lung sections were deparaffinized following the same procedure as described above. The sections were stained with Weigert’s iron hematoxylin solution (Beyotime, Cat. No. C0189S) for 10 min at room temperature, rinsed in running water for 5 min to remove excess staining, stained with Ponceau acid fuchsin staining solution for 10 min at room temperature, rinsed in running water for 10 s, differentiated in phosphomolybdic acid solution for 2 min, and then rinsed. Finally, the sections were stained with aniline blue staining solution for 1 min, rinsed in running water for 10 s, mounted with neutral gum (Solarbio), and observed and photographed using an optical microscope (OLYMPUS). In the trichrome stain, cell nuclei appeared purple, muscle fibers and cytoplasm appeared red, and collagen fibers appeared blue. ImageJ software (V1.8.0) was used to analyze the number of lung lobes and the level of fibrosis. For Sirius Red staining, lung tissue sections were deparaffinized following the standard procedure described above. Sections were then stained with Weigert’s iron hematoxylin solution for 5–10 min, followed by a brief rinse in distilled water for 10–20 s to remove excess stain. Sections were washed under running tap water for 5 min. Subsequently, sections were stained with Sirius Red solution (Servicebio, #G1078) for 15–30 min, then rinsed thoroughly under running water to remove surface dye. Dehydration was performed sequentially in 75% ethanol for 1 min, 95% ethanol for 1 min, and absolute ethanol for 1 min, followed by three changes of xylene, each for 1–2 min. Finally, sections were mounted with neutral resin (Solarbio) and examined under an optical microscope (OLYMPUS) for imaging. Collagen fibers appeared red, nuclei ranged from brownish to black, and muscle fibers stained yellow. ImageJ software (V1.8.0) was used to analyze the level of fibrosis. For measurement of the hydroxyproline (HYP) content in mouse lung tissues using the assay kit (BC0250, Solarbio), approximately 0.2 g of lung tissue was homogenized with 2 mL extraction solution. The mixture was boiled and digested at 16,000 rpm for 2–4 h until no obvious clumps were present. After cooling, the pH was adjusted to 6–8 with 10 mol/L NaOH. The volume was brought to 4 mL with distilled water, and the supernatant was collected after centrifugation at 16,000 rpm for 20 min. A standard solution with a concentration of 0.5 mg/mL was prepared by diluting a series of concentrations. Blank, sample, and standard tubes were prepared. After heating in a water bath at 60 °C for 20 min and standing at room temperature for 15 min, the optical density (OD) was measured at 560 nm. The ∆A (A standard tube - A blank tube) was calculated, and then a standard curve was plotted using the equation y = kX + b. The ∆A value for the test tube was substituted into the equation to calculate X (µg/mL). Finally, the tissue hydroxyproline content (µg/g) was calculated as X × sample volume (V1) / (sample mass (W) × added sample volume (V1) / volume of the tissue extract (V2)).

### Real-time quantitative polymerase chain reaction (RT-qPCR)

Total RNA was extracted from cells and tissues using Trizol reagent (Thermo Fisher Scientific, Cat. No. 15596026) according to the manufacturer’s instructions. cDNA was synthesized using the RevertAid First Strand cDNA Synthesis Kit (Thermo Scientific, Cat. No. K1622). The resulting cDNA was used for real-time quantitative PCR (RT-qPCR) with the Genious 2× SYBR Green Fast qPCR Mix (ABclonal, Cat. No. RK21204) system for gene amplification. Quantitative analysis was performed using the 2^−∆∆Ct^ method, with GAPDH used as an endogenous reference. Primer design and synthesis were provided by Sangon Biotech.

m_*Prdx4*: F: TCCTGTTGCGGACCGAATC, R: CCACCAGCGTAGAAGTGGC;

m_*Il-1α*: F: CGAAGACTACAGTTCTGCCATT, R: GACGTTTCAGAGGTTCTCAGAG;

m_*Il-1β*: F: GCAACTGTTCCTGAACTCAACT, R: ATCTTTTGGGGTCCGTCAACT;

m_*Il-6*: F: TAGTCCTTCCTACCCCAATTTCC, R: TTGGTCCTTAGCCACTCCTTC;

m_*Tnf-α*: F: CCCTCACACTCAGATCATCTTCT, R: GCTACGACGTGGGCTACAG;

m_*Tgf-β*: F: CTCCCGTGGCTTCTAGTGC, R: GCCTTAGTTTGGACAGGATCTG;

m_*α-sma*: F: GTCCCAGACATCAGGGAGTAA, R: TCGGATACTTCAGCGTCAGGA;

m_*Col1a1*: F: GCTCCTCTTAGGGGCCACT, R: CCACGTCTCACCATTGGGG;

m_*Col3a1*: F: CTGTAACATGGAAACTGGGGAAA, R: CCATAGCTGAACTGAAAACCACC;

m_*Gapdh*: F: AGGTCGGTGTGAACGGATTTG, R: TGTAGACCATGTAGTTGAGGTCA;

h_*PRDX4*: F: AGAGGAGTGCCACTTCTACG, R: GGAAATCTTCGCTTTGCTTAGGT;

h_*IL-1α*: F: AGATGCCTGAGATACCCAAAACC, R: CCAAGCACACCCAGTAGTCT;

h_*IL-1β*: F: ATGATGGCTTATTACAGTGGCAA, R: GTCGGAGATTCGTAGCTGGA;

h_*IL-6*: F: ACTCACCTCTTCAGAACGAATTG, R: CCATCTTTGGAAGGTTCAGGTTG;

h_*TNF-α*: F: GAGGCCAAGCCCTGGTATG, R: CGGGCCGATTGATCTCAGC;

h_*TGF-β*: F: CAATTCCTGGCGATACCTCAG, R: GCACAACTCCGGTGACATCAA;

h_*a-SMA*: F: CTATGAGGGCTATGCCTTGCC, R: GCTCAGCAGTAGTAACGAAGGA;

h_*COL1A1*: F: GAGGGCCAAGACGAAGACATC, R: CAGATCACGTCATCGCACAAC;

h_*COL3A1*: F: TTGAAGGAGGATGTTCCCATCT, R: ACAGACACATATTTGGCATGGTT;

h_*GAPDH*: F: ACAACTTTGGTATCGTGGAAGG, R: GCCATCACGCCACAGTTTC.

### Design of mutant site and construction of overexpressed plasmid

Protein site-directed mutagenesis and plasmid construction were performed as described previously.^59^ In summary, based on the principles of amino acid site mutation, the mutated amino acids needed to best preserve the structure and function of the original protein, considering factors such as atomic composition, polarity, charge, and size. Using the disease mutation sites and protein structure visualization tool VarSite (https://www.ebi.ac.uk/thornton-srv/databases/VarSite),^41^ simulations were performed to mutate four cysteine residues (51, 124, 148, and 245) of the PRDX4 protein (Uniprot ID: Q13162). The impact of the mutated amino acids on the structure and function of the PRDX4 protein was simulated and analyzed, and mutation sites that had minimal impact on the protein structure and function were selected. The results showed that the optimal mutations for Cys (51, 124, and 245) were to serine (S), and the optimal mutation for Cys148 was to aspartic acid (D). Based on these optimal mutation sites, the wild-type plasmid H_PRDX4 WT, mutant plasmids H_PRDX4 (p.C51S) Mut1, H_PRDX4 (p.C124S) Mut2, H_PRDX4 (p.C148D) Mut3, and H_PRDX4 (p.C245S) Mut4 were constructed. The pcDNA3.1-MCS-EF1-ZsGreen plasmid (Genomeditech, Cat. No. GM-8630P1) was used as the destination gene expression vector, and the E. coli strain stbl3 (Thermo Fisher, Cat. No. G1036) was prepared for transformation. PRDX4 primers were designed and synthesized with the homologous recombination sequence added at the 5′ end. The designed primer sequences were sent to a primer synthesis company (Suzhou Jinweizhi Biotechnology Co., Ltd.) for synthesis. After plasmid digestion, Sanger sequencing validation, and extraction (TIANGEN, Cat. No. DP107), the pcDNA3.1-H_PRDX4-3×HA-EF1-ZsGreen1WT, pcDNA3.1-H_PRDX4(p.C51S)-3×HA-EF1-ZsGreen1 Mut1, pcDNA3.1-H_PRDX4(p.C124S)-3×HA-EF1-ZsGreen1 Mut2, pcDNA3.1-H_PRDX4(p.C148D)-3×HA-EF1-ZsGreen1 Mut3, and pcDNA3.1-H_PRDX4(p.C245S)-3×HA-EF1-ZsGreen1 Mut4 overexpression plasmids were obtained. Using pcDNA3.1-MCS-EF1-ZsGreen as the destination gene expression vector plasmid, the steps were the same as mentioned earlier, and the pcDNA3.1-H_PTEN-3×Flag-EF1-ZsGreen1WT wild-type overexpression plasmid was constructed.

### Plasmid and siRNA transfection

In plasmid transfection, HEK293T cells (Pricella) were seeded the day before transfection in a 60 mm dish (Pricella), with the cell density reaching 60% to 80% for transfection. Six hours prior to transfection, the cells were switched to non-complete MEM (Pricella) medium. The plasmid (2 μg) was diluted in 400 μl of Opti-MEM™ I (Thermo Fisher Scientific, Cat. No. 31985070), and Lipofectamine™ 2000 (Thermo Fisher Scientific, Cat. No. 11668500) was diluted in another 40 μl of Opti-MEM™ I (8 μl). After mixing the solutions gently, the Lipofectamine™ 2000 mixture was slowly added to the plasmid mixture, incubated for 10 min at room temperature. The prepared transfection solution was then added to the cell culture dish, gently mixed, and the cells were incubated at 37 °C with 5% CO_2_ in a culture incubator (Panasonic) for 6 h. The medium was then replaced with complete MEM, and the expression of green fluorescent protein (ZsGreen) in HEK293T cells was observed under a fluorescence microscope (Leica) 24 h post-transfection to assess plasmid transfection efficiency. In siRNA transfection, the cells were seeded in a 35 mm dish the day before transfection, with the cell density reaching 60–80% for transfection. Six hours prior to transfection, the cells were switched to non-complete RPMI 1640/DMEM medium (without FBS and without P/S). The siRNA (20 pmol) was diluted in 200 μl of serum-free medium, and Lipofectamine™ 2000 (4 μl) was diluted in another 200 μl of serum-free medium. The siRNA and Lipofectamine™ 2000 solutions were mixed and added to the cells in the same manner as for plasmid transfection. After incubation for 6 h at 37 °C with 5% CO_2_ in a culture incubator, the medium was replaced with complete RPMI 1640/DMEM. The expression levels of the target gene and protein were detected by RT-QPCR and WB at 24 and 48 h post-transfection, respectively. The siRNAs and their corresponding negative controls used in this study were purchased from Genepharma (Shanghai, China). The siRNA sequences for mouse cell lines were as follows: si_NC: ACGUGACACGUUCGGAGAATT, si_*Prdx4*_573: AUUAAUCCAGGCCAAGUGGTT. For human cell lines, the siRNA sequences were: si_NC: UUCUCCGAACGUGUCACGUTT, si_*PRDX4*_531: CUGGCAGUGACACGAUUAATT.

### Reducing and non-reducing SDS-PAGE

Reducing and Non-Reducing SDS-PAGE were performed as previously described.^59,65^ Briefly, treated cells were lysed in RIPA buffer containing 1×PMSF (100 mM) (Beyotime, Cat. No. ST506) and 20 mM N-Ethylmaleimide (NEM) (Sigma, Cat. No. E376) on ice for 30 min. After centrifugation at 4 °C for 30 min, the supernatant was collected and the protein concentration was determined using a BCA protein quantification kit (Thermo Scientific Pierce, Cat. No. A55864). For the separation of cytoplasmic and nuclear proteins, the NE-PER™ nuclear and cytoplasmic extraction kit (Thermo Scientific Pierce, Cat. No. 78833) was utilized. In Reducing SDS-PAGE, an equal amount of protein was mixed with 5× SDS-PAGE loading buffer (Beyotime, Cat. No. P0015L) and boiled at 100 °C for 10 min. Thiol-reducing agent TCEP (5 mM) (Beyotime, Cat. No. ST045) was added and incubated for 30 min prior to separation on 10% NuPAGE™ Tris-Glycine gels (constant voltage of 60 V for 45 min for the stacking gel and 110 V for 1 h for the separating gel). In Non-Reducing SDS-PAGE, an equal amount of protein was mixed with 4× NuPAGE™ LDS sample buffer (Thermo Fisher Scientific, Cat. No. NP0007) and heated at 70 °C for 10 min prior to separation on the same gels. Following separation, all proteins were transferred to 0.2 μm PVDF membranes (Immobilon, Cat. No. ISEQ00010) at room temperature. The membranes were blocked with 5% BSA for 1 h at room temperature. After TBST washing, primary antibodies were added and incubated overnight at 4 °C, followed by secondary antibodies at room temperature for 1 h (Millipore, Cat. No. WBKLS0100, incubated for 30 s). Protein bands were visualized using an imaging system (Amersham ImageQuant™ 800, 29399481), and band intensities were analyzed using ImageJ software (V1.8.0). The following antibodies were used: PRDX4 (1:1000, ab59542, Abcam), PTEN (1:1000, 9552, CST), β-Actin (1:10,000, AC026, Abclonal), α-SMA (1:1000, 14968, CST), COL1A1 (1:1000, ab138492, Abcam), COL3A1 (1:1000, 30565, CST), E-Cadherin (1:1000, A20798, Abclonal), N-Cadherin (1:500, A3045, Abclonal), Vimentin (1:500, A2584, Abclonal), Phospho-PI3K p85 (1:1000, 4228T, CST), Phospho-Akt (Thr308) (1:1000, 13038T, CST), Phospho-TAK1(Thr184/187) (1:1000, 4508s, CST), Phospho-NF-kB p65 (1:1000, AP0123, ABclonal), Phospho-c-Jun-S73 (1:500, AP0119, ABclonal), HistoneH3 (1:2000, A2348, Abclonal). The corresponding secondary antibodies were HRP-conjugated Goat anti-Rabbit IgG (1:5000, AS014, Abclonal).

### ROS detection

After PBMC-m and AMs cells were stimulated with CS (50 μg/cm²) for 48 h, DCFH-DA (S0033S, Beyotime) was diluted 1:1000 in serum-free culture medium to a final concentration of 10 μM. Subsequently, 1 mL of the diluted DCFH-DA was added to the 6-well plates and incubated in a cell culture incubator at 37 °C for 20–30 min. After incubation, the cells were washed three times with serum-free culture medium to thoroughly remove DCFH-DA that had not entered the cells. Finally, the cells were observed and imaged using a fluorescence microscope (Leica, DMI3000B).

### Recombinant active protein reaction and detection

To investigate the effects of H_2_O_2_ treatment on protein expression, 1 μg of recombinant active PRDX4 (ab93947, Abcam) and PTEN (ab157087, Abcam) proteins were incubated at 37 °C with 100 μM H_2_O_2_ for 30 min. The expression of PRDX4 (monomer, dimer, HWM oligomer) and PTEN (monomer, dimer) was detected under both Reducing and Non-Reducing conditions using Coomassie Blue staining. For the detection of PTEN addition post-H_2_O_2_ treatment, 1 μg of recombinant active PRDX4 protein was treated with H_2_O_2_ for 30 min, followed by the removal of H_2_O_2_ and the addition of 1 μg of recombinant PTEN protein, which was then incubated at 37 °C for 2 h. Non-Reducing SDS-PAGE was used to detect the expression of PRDX4 and PTEN proteins under Non-Reducing conditions. To assess the impact of Con A on PRDX4 oligomer formation, 1 μg of recombinant PRDX4 protein was first treated with 100 μM H_2_O_2_ for 30 min, followed by the addition of 20 mM Con A (PRDX4 protease inhibitor) and incubation at 37 °C for 30 min. The expression pattern of the PRDX4 protein was detected using Coomassie Blue staining. To evaluate the effect of the sequence of Con A and H_2_O_2_ treatment on PRDX4 expression, 1 or 2 μg of recombinant PRDX4 protein was first incubated with 20 mM Con A at 37 °C for 30 min, followed by the addition of 100 μM H_2_O_2_ for 30 min, and PRDX4 protein expression was detected using Coomassie Blue staining.

### Co-immunoprecipitation assays

Immunoprecipitation experiments were conducted as previously described.^23^ In brief, 1 × 10^6^ cells were collected by washing with cold PBS (Contains 10 mM NEM) and lysed on ice for 30 min with 1 ml of lysis buffer (Tris-HCl 50 mM, pH 7.4, NaCl 150 mM, Sodium Deoxycholate 0.25%, NP-40 1%, EDTA 1 mM, PMSF 1 mM, 20 mM NEM, 200 U catalase).The cells were then centrifuged at 4 °C at 12600 rpm for 15 min. The supernatant was collected and incubated overnight at 4 °C with the corresponding primary antibody to form the protein-antibody complex. Subsequently, 10 μL of protein A/G agarose beads (Santa Cruz Biotech, Cat. No. sc-2003) were added and incubated for 3 h to deplete endogenous immunoglobulin G and enrich the primary antibody/protein complex. The complex was washed three times with lysis buffer and then incubated with 5× SDS-PAGE sample buffer at 100 °C for 10 min. SDS-PAGE was performed to detect the corresponding protein expression. The protein bands were visualized using an imaging system, and the band intensities were analyzed using ImageJ software (V1.8.0). The following antibodies were used: FLAG (1:100, #2368, CST) and HA (1:1000, #3724, CST).

### Endoplasmic reticulum isolation

The endoplasmic reticulum (ER) and cytoplasmic (endoplasmic reticulum-free) fractions of AM cells were isolated using an ER isolation kit (ER0100, Sigma-Aldrich). Briefly, for AMs cells, cells were collected and incubated for 20 min at 4 °C using 1× hypotonic extraction buffer (10 mM HEPES (pH 7.8), 25 mM KCl, and 1 mM EGTA) for 20 min to allow for sufficient expansion and rupture of the cells. centrifugation was performed for 5 min at 600 × *g*, and then the supernatant was aspirated off, the precipitate was retained, and based on the volume of precipitated PCV cells, 1× isotonic Extraction buffer (10 mM HEPES (pH 7.8), 250 mM sucrose, 25 mM KCl, and 1 mM EGTA) was added, and the cells were broken using a Dounce homogenizer and subjected to differential centrifugation. The homogenate was centrifuged at 1000 × *g* for 10 min at 4 °C, and the supernatant was collected and the supernatant was centrifuged at 12,000 × *g* for 15 min at 4 °C, and the supernatant was collected, and this supernatant fraction was the postmitochondrial fraction (PMF). Subsequently, the PMF supernatant was precipitated using CaCl₂ (8 mM), followed by medium-speed centrifugation (8000 × *g*), and the precipitate was collected to obtain enriched rough endoplasmic reticulum (RER) microsomes.

### CIBERSORTx and TRRUST database analysis

RNA-seq data from silicosis patients and mouse lung tissues were imported into CIBERSORTx (https://cibersortx.stanford.edu). Subsequently, the “LM22” signature matrix was selected from the “Signature Matrix” dropdown menu to analyze the expression levels of the 22 human immune cell types identified (P < 0.05). The transcription factors (TFs) that regulate human and mouse inflammatory factors *TNF-α, TGF-β, IL-1* (*IL-1α, IL-1β*), and *IL-6* were searched using the TRRUST database (http://www.grnpedia.org). Detailed information is provided in Supplementary Table S4.

### Statistical analysis

Statistical analyses were performed using Rv4.0.2 or GraphPad Prism 9 (GraphPad Software Inc., San Diego, USA). All results were expressed as the mean ± standard deviation (SD). Two-group comparisons were performed using an unpaired, two-tailed Mann–Whitney U-test, and multi-group comparisons were performed using one-way ANOVA with Tukey´s or Bonferroni´s multiple comparison test. Differences were considered statistically significant at P < 0.05. *P ≤ 0.05, **P ≤ 0.01, ***P ≤ 0.001, and NS, not significant.

## Supplementary information

Supplementary information

Supplementary information

## Data Availability

All data needed to evaluate the conclusions in the paper are present in the paper and/or the Supplementary Materials. The 201 inflammatory genes and 307 fibrosis genes used in this research were obtained from the GSEA database (http://www.gsea-msigdb.org/). The sequencing data of mouse and human lung tissues was obtained from (https://www.thno.org/v11p2381) and the GSA database (https://ngdc.cncb.ac.cn/gsa-human/HRA000560). Screening data for transcription factors that regulate inflammatory factors came from the TRRUST database (https://www.grnpedia.org/).
