# Supplementary material for: Peroxiredoxin 4 as a switch regulating PTEN/AKT axis in alveolar macrophages activation
Source: Signal Transduct Target Ther. 2025 Oct 24;10:352. doi: 10.1038/s41392-025-02454-x (PMC12550102; doi:10.1038/s41392-025-02454-x)
Supplement: Supplementary file 1 — Supplementary information [file 41392_2025_2454_MOESM1_ESM.docx]

Supplementary Materials for

**Peroxiredoxin 4 as a switch regulating PTEN/AKT axis in alveolar macrophages activation**

Jia-Wei Zhou^1,2,3,7^, Ying Bai^1,2,3,7^, Jian-Qiang Guo^1,3,7^, Yun-Yun Li^1,3^, Ya-Feng Liu^1,3^, Chao Liang ^1,3^, Ying-Ru Xing^3,4,5^, Hai-Long Guo^6^, Tian-Xiang Qi^1,3*^, Jing Wu^1,3,4*^, Dong Hu^1,2,3,4,8*^

Correspondence to: dhu@aust.edu.cn

**This PDF file includes:**

Figures. S1 to S7

Tables. S1 to S6

**Other Supplementary Materials for this manuscript include the following:**

Original images of Western blots

***Supplementary Figures S1-S7***


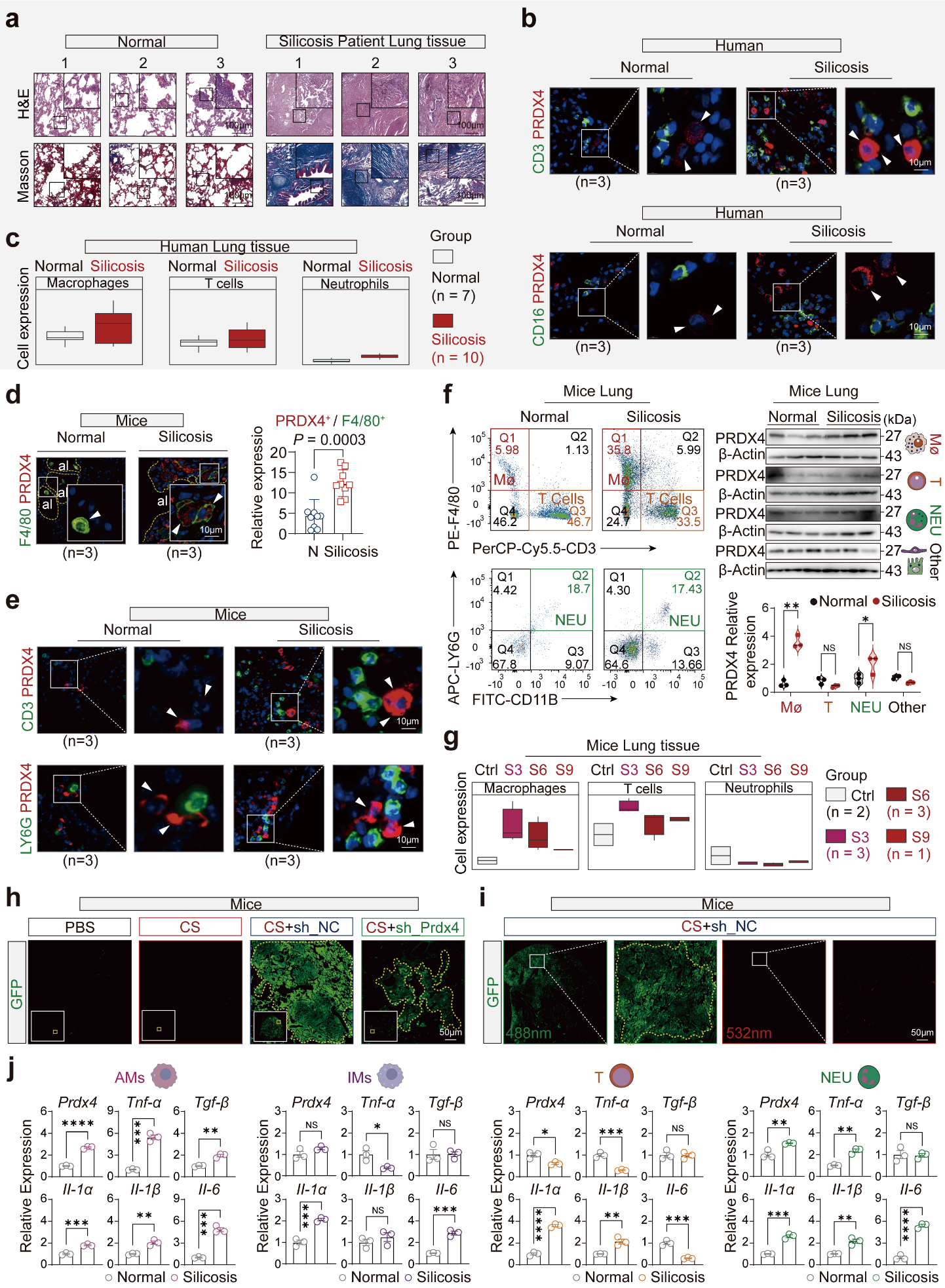


***Figure. S1. PRDX4 in AMs promotes silicosis fibrosis***

**a** H&E and Masson staining of lung tissues from normal (n = 3) and silicosis patients (n = 3), with the inset showing the regions of silicotic nodules and collagen deposition. Normal and silicosis lung tissues were obtained from lung transplantation surgeries. **b** Immunofluorescence (IF) analysis of PRDX4 in lung tissue T and neutrophil cells from normal (n = 3) and silicosis patients (n = 3). The right panel shows a high-magnification view of the selected area.

**c** The abundance of T cells, macrophages, and neutrophils in the lungs of normal individuals (n = 7) and those with silicosis (n = 10) was determined using gene expression profiling data from lung tissues and the CIBESORTx online tool. **d** Immunofluorescence (IF) analysis of PRDX4 in AMs cells from the lung tissue of normal and silicosis mice (n = 3). The inset shows a high-magnification view of the selected area. White arrows indicate AMs cells, and the yellow dashed line indicates the lung parenchyma. The right bar graph shows the relative expression ratio of PRDX4 protein to mouse macrophages (F4/80), with three fields of view analyzed per sample, and each point representing one field of view. **e** Immunofluorescence (IF) analysis of PRDX4 in lung tissue T and neutrophil cells from normal (n=3) and silicosis mice (n=3). The right panel shows a high-magnification view of the selected area. **f** Flow cytometric sorting of CD45^+^F4/80^+^Mø (red area), CD45^+^CD3^+^ T cells (yellow area), and CD45^+^CD11B^+^LY6G^+^ neutrophils (green area) in normal and silicosis mouse lung tissues to detect PRDX4 protein expression. The right table shows the expression and quantitative analysis results of PRDX4 protein in various cells of normal (n = 3) and silicosis mouse (n = 3) lung tissues. **g** The abundance of T cells, macrophages, and neutrophils in the lungs of mice with normal conditions and various stages of silicosis was calculated using gene expression profiling data and the CIBESORTx tool. S3, S6, and S9 denote 3, 6, and 9 weeks of CS exposure in mice, respectively. **h**, **i** Green fluorescent protein (GFP) expression in lung tissues from four groups of mice was assessed using a 488 nm laser (GFP excitation wavelength) to measure GFP fluorescence intensity, indicative of lung infection efficiency. **j** After flow cytometric sorting of AMs, IMs, T cells, and neutrophils from lung tissues of normal and silicosis mice, the expression of *Prdx4* and related inflammatory cytokines (*Tnf-α, Tgf-β, Il-1α, Il-1β, IL-6*) was measured. Each data point represents an individual mouse, and the experiment was repeated three times. Scale bars: 10 µm (**b**, **d**, **e**), 50 µm (**h**, **i**). The box plot (**c**, **g**) illustrates the median and 25th to 75th percentiles, with whiskers representing the minimum and maximum values. Statistical significance and P values were determined using a two-tailed unpaired t-test (**d**, **j**) and Multiple t-tests (**f**). NS indicates not significant; *P ≤ 0.05; **P ≤ 0.01; ***P ≤ 0.001.


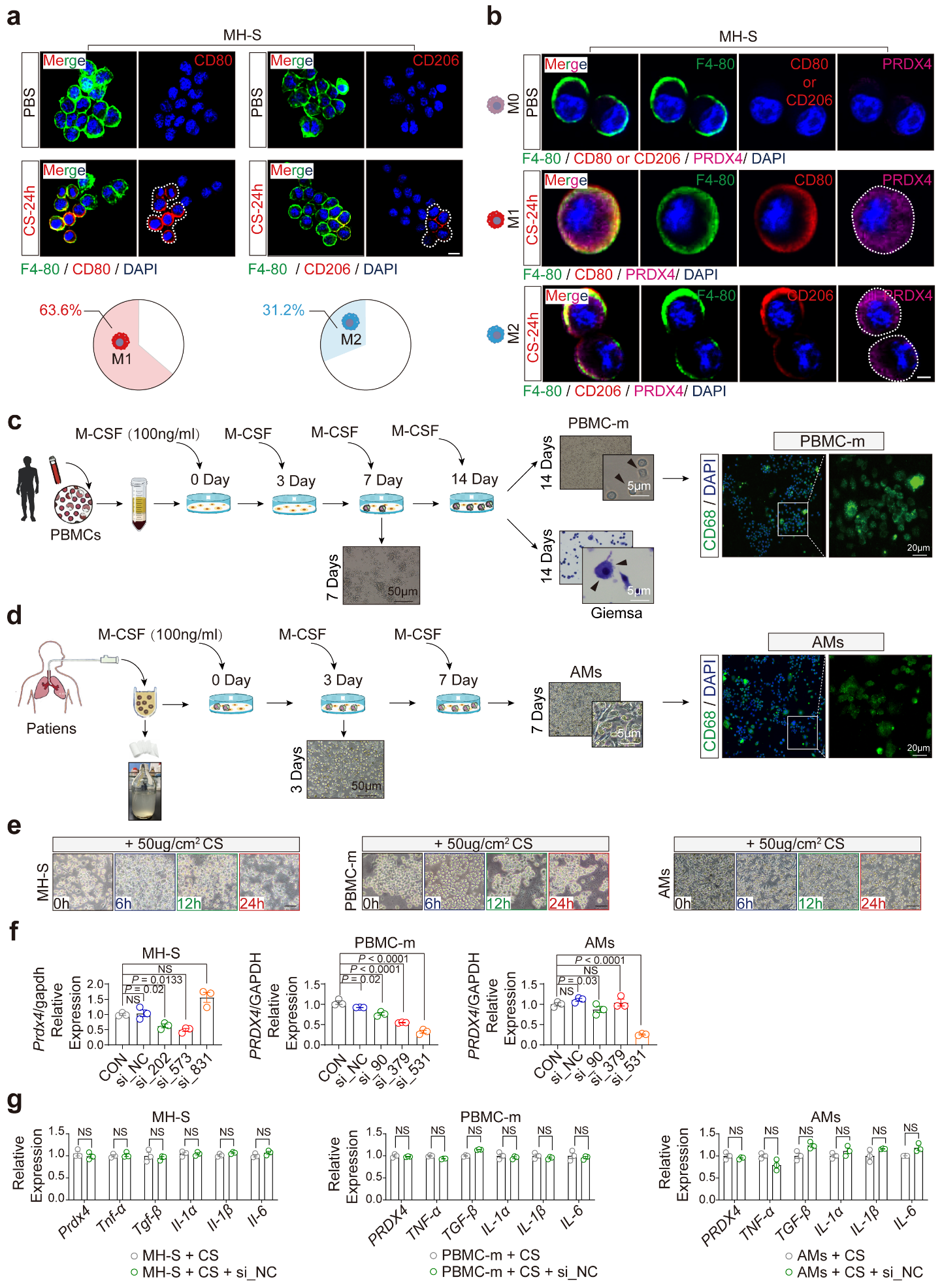


***Figure. S2. Culture and Identification of Macrophages***

**a** Immunofluorescence analysis of MH-S cells stimulated with CS (50 µg/cm²) for 24 hours, showing changes in M1 (F4-80⁺CD80⁺) and M2 (F4-80⁺CD206⁺) macrophage populations. The inset pie chart displays the proportions of M1 and M2 cells within the MH-S population. **b** Immunofluorescence analysis of PRDX4 expression levels in M1 and M2 macrophages following 24-hour CS (50 µg/cm²) stimulation of MH-S cells. **c** Schematic depiction of the induction and identification of peripheral blood mononuclear cell-derived macrophages (PBMC-m). PBMCs were sourced from healthy volunteers and induced into PBMC-m cells by continuous cultivation with macrophage colony-stimulating factor (M-CSF) at 100 ng/ml for 14 days (indicated by black arrows). Cell morphology was assessed using Giemsa staining, and cell purity was identified by detecting CD68 expression via immunofluorescence (IF). **d** Schematic overview of the culture and identification of AMs. Bronchoalveolar lavage fluid (BALF) from normal miners was processed by filtering through sterile gauze to eliminate mucus, followed by centrifugation. Cultivation with M-CSF (100 ng/mL) for 7 days allowed for the observation of cell morphology and assessment of cell purity by detecting CD68 expression through IF. **e** Morphological changes of MH-S, PBMC-m, and AMs cells after stimulation with CS (50 μg/cm^2^) for 0 h, 6 h, 12 h, and 24 h. **f** Assessment of the knockdown efficiency of three si_PRDX4 sequences in mouse (MH-S) and human (PBMC-m, AMs) macrophages. The experiment was conducted three times. **g** PRDX4 and associated inflammatory gene expression in MH-S, PBMC-m, and AMs cells was analyzed following transfection with PBS and si_NC, subsequent to 24-hour CS (50 µg/cm²) stimulation. The experiment was conducted three times.

Scale bars: 5 µm (**b**, **c**, **d**), 20 µm (**a**, **c**, **d**), 50 µm (**c**, **d, e**). Data are presented as mean ± standard error of the mean (SEM). Statistical significance was determined using One-Way ANOVA (**f**) and Multiple t tests (**g**). NS, not significant.


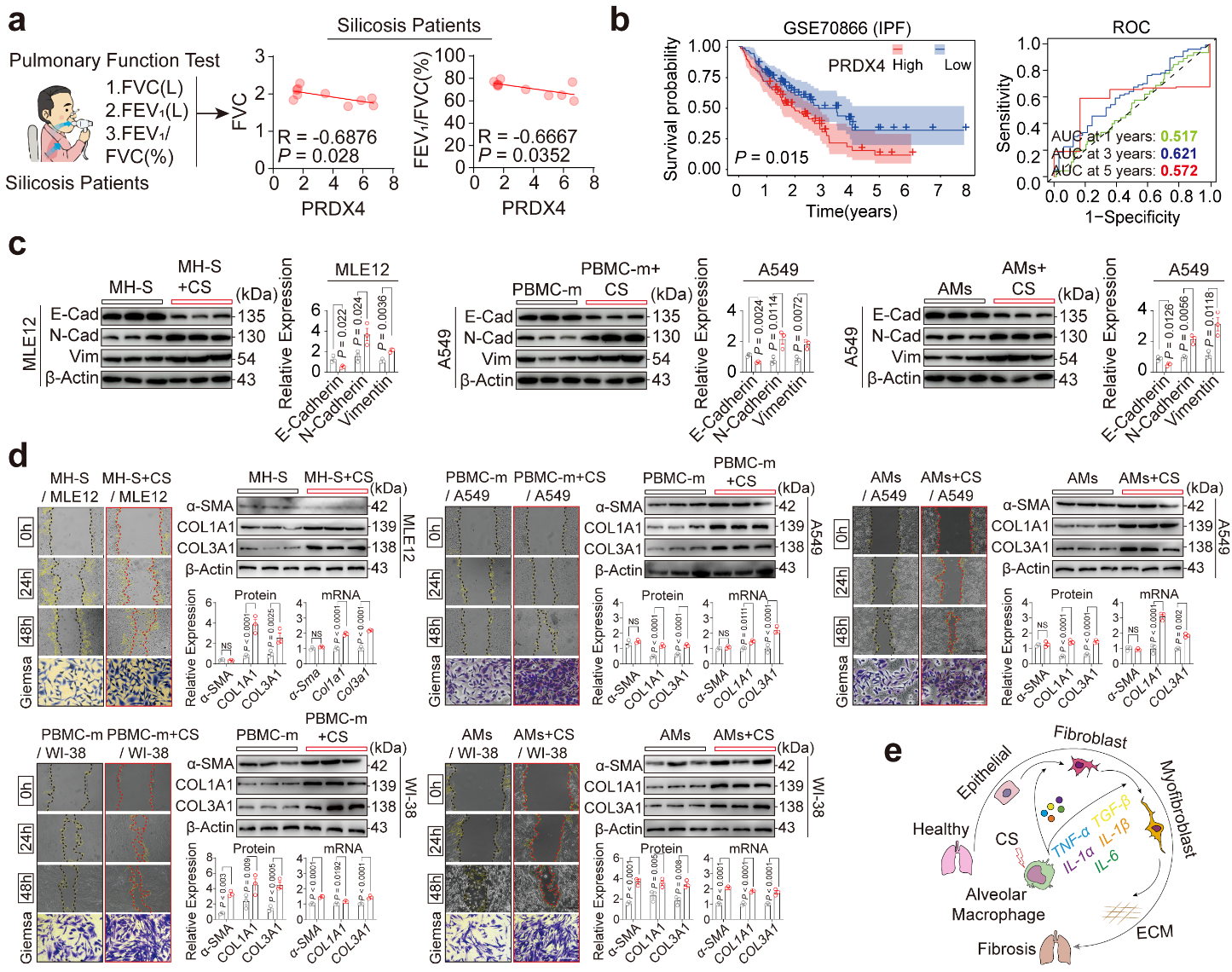


***Figure S3. CS-activated macrophages promote epithelial-to-mesenchymal transition (EMT) in epithelial cells and fibroblast-to-myofibroblast transition (FMT).***

**a** Correlation analysis between PRDX4 gene expression and pulmonary function parameters (FVC and FEV1/FVC) in silicosis patients (n = 10). **b** Analysis of PRDX4 gene expression and patient prognosis in BALF samples from 176 IPF patients in the GSE70866 dataset; right panel shows ROC curves for PRDX4 predicting 1-, 3-, and 5-year prognosis in IPF patients, with area under the curve (AUC) indicating predictive performance. **c** Effects of CS-stimulated macrophages on epithelial cell protein expression of E-Cadherin, N-Cadherin, and Vimentin in macrophage/epithelial co-culture models (MH-S/MLE12, AMs/A549, PBMC-m/A549). **d** Development and evaluation of macrophage/epithelial (MH-S/MLE12, AMs/A549, PBMC-m/A549) and macrophage/fibroblast (AMs/WI-38, PBMC-m/WI-38) co-culture models. Left: scratch assay and Giemsa staining to assess epithelial and fibroblast migration and proliferation. Right: analysis of α-SMA, COL1A1, and COL3A1 gene and protein expression in cells after 48 hours of culture. The experiment was conducted three times. MLE12: mouse alveolar type II epithelial cells; A549: human alveolar type II epithelial cells; WI-38: human lung fibroblasts. **e** Schematic representation illustrating that aberrantly activated AMs can secrete inflammatory factors, thereby promoting EMT and FMT processes and perpetuating lung fibrosis.

Scale bars: 50 µm (**d**). Correlation analysis was performed using Pearson's test (**a**). Data are presented as mean ± standard error of the mean (SEM). Statistical significance was determined using Multiple t tests (**c**, **d**). NS, not significant.


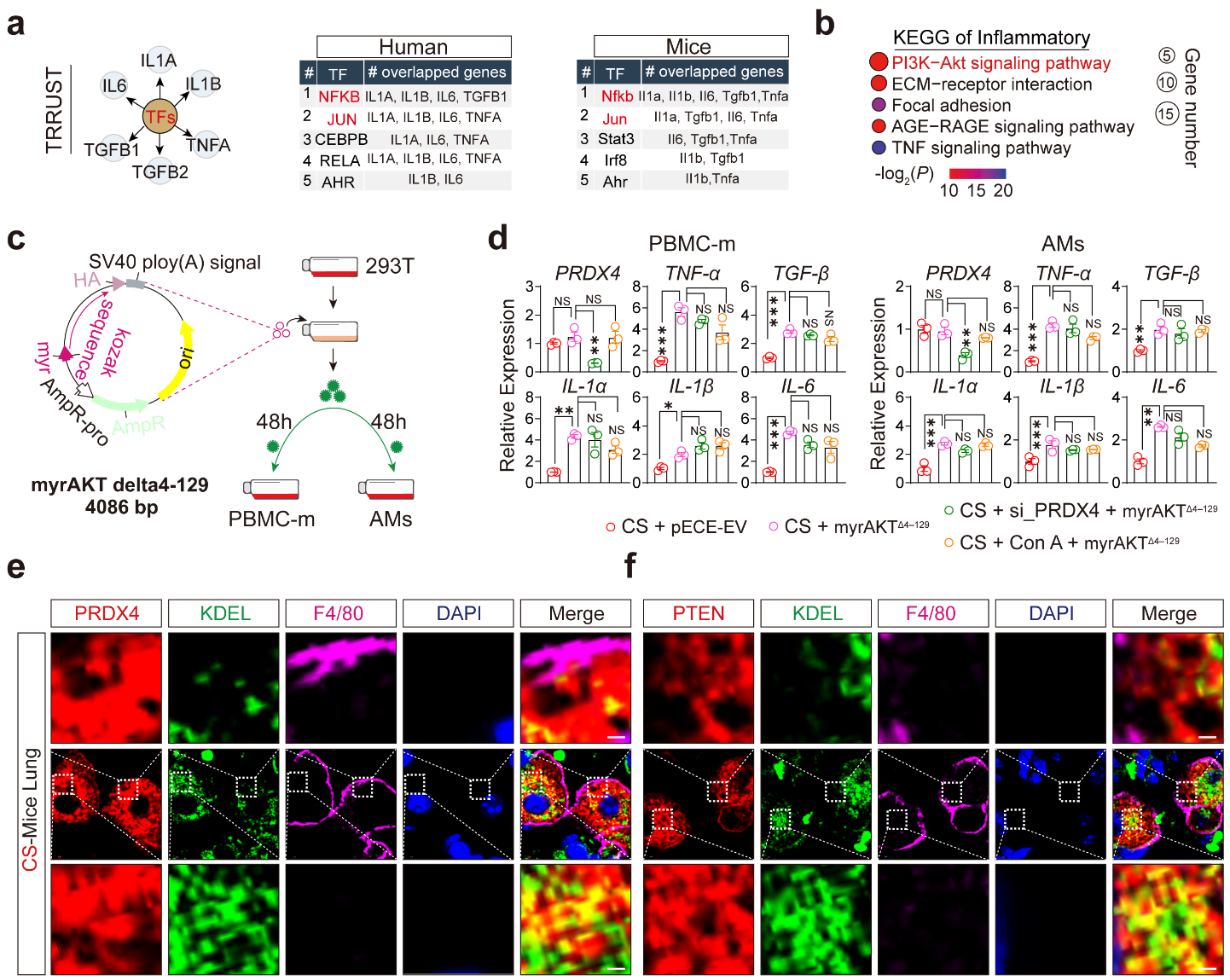


***Figure S4. PRDX4 Regulates AMs Activation Through the AKT/NF-κB Pathway***

**a** Enriched transcription factors (TFs) that regulate the expression of human and mouse-related inflammatory and fibrotic factors. The left side illustrates the schematic diagram of searching for TFs that regulate the expression of inflammatory and fibrotic factors using the transcription factor regulation network TRRUST database, while the right table lists the key TFs in humans and mice and their regulated genes. **b** KEGG enrichment analysis of key pathways related to inflammation, based on the number and significance of genes enriched in each pathway. **c** The flowchart depicting the transfection of pECE-EV and myrAKT^Δ4-129^ plasmids into PBMC-m and AMs cells, respectively. **d** On the basis of transfecting PBMC-m and AMs cells with pECE-EV and myrAKT^Δ4-129^ plasmids, the cells were treated with si_PRDX4 and MK-2206 (5 μM) following stimulation with CS (50 μg/cm^2^) for 48 hours. Subsequently, the expression levels of *Prdx4* and related inflammatory cytokines (*Tnf-α*, *Tgf-β*, *Il-1α*, *Il-1β*, *IL-6*) were assessed. The experiment was repeated three times. **e**, **f** Immunofluorescence (IF) analysis of PRDX4 (**e**) and PTEN (**f**) distribution in the endoplasmic reticulum (KDEL, green) in macrophages (F4/80, pink) of lung tissue from silicosis mice, top and low side panels show high magnification views of selected areas of the white dashed line.

Scale bars: 1 µm (**e**, **f**). Data are presented as mean ± standard error of the mean (SEM), with statistical significance determined by One-Way ANOVA(**d**). The KEGG pathway enrichment analysis bubble diagram (**b**) was generated using the “clusterProfiler” package in R software version 4.0.2. NS, not significant; *P ≤ 0.05; **P ≤ 0.01; ***P ≤ 0.001.


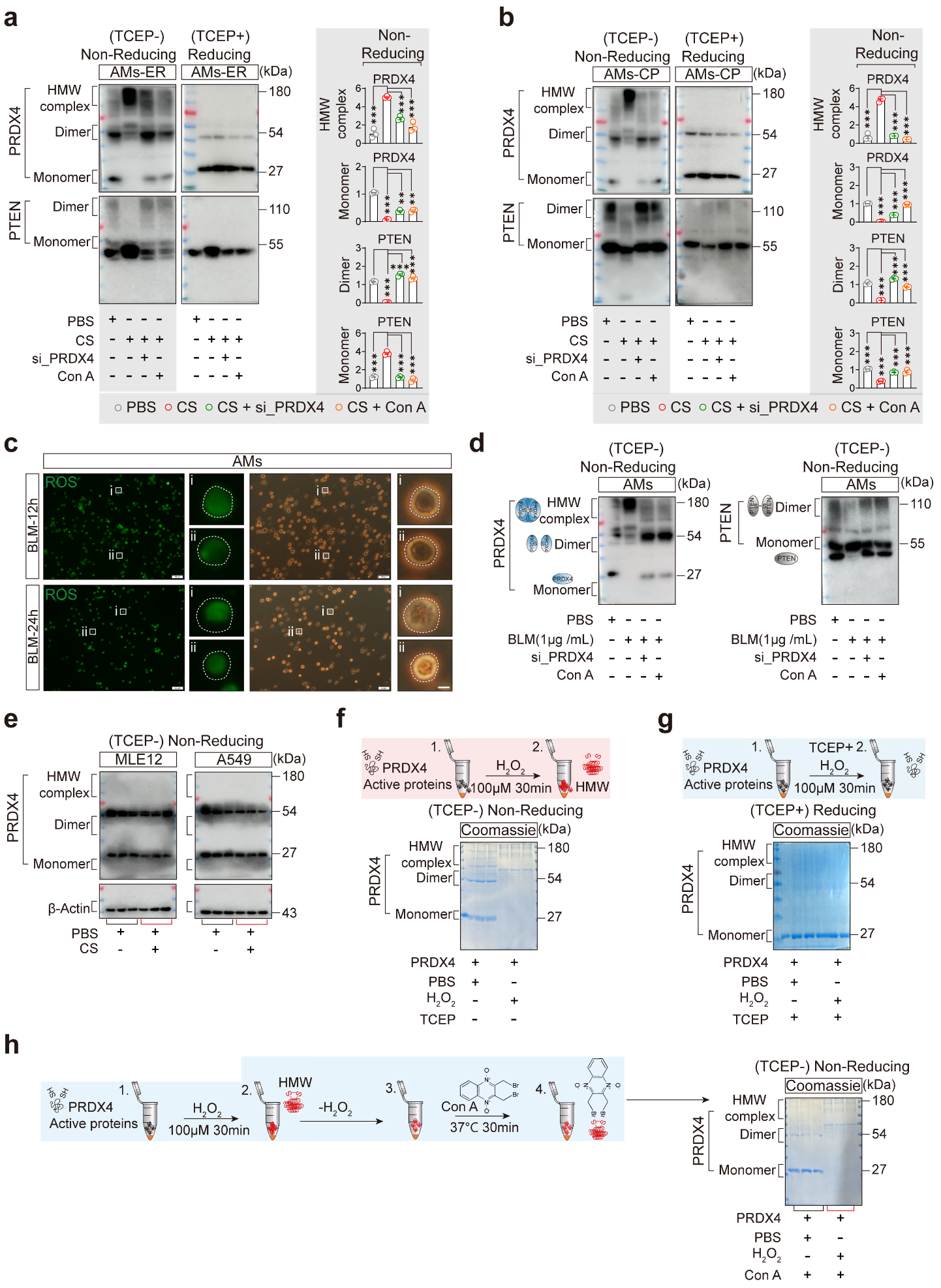


***Figure S5. Con A Inhibits the Formation of PRDX4 HWM Oligomers from monomer***

**a**, **b** Non-Reducing and Reducing SDS-PAGE analysis of protein expression of PRDX4 (monomer, dimer, obligomer) and PTEN (monomer, dimer) in endoplasmic reticulum (**a**) and cytoplasm without endoplasmic reticulum components (**b**) of AMs cells. AMs cells were treated with si_PRDX4 and Con A (10 μM) after stimulation with CS (50 μg/cm²), and the bar graphs on the right side show the results of quantitative analysis of PRDX4 and PTEN proteins; the experiment was repeated three times. **c.** After stimulation of AMs with BLM (1 μg/ml) for 12 and 24 hours, intracellular reactive oxygen species (ROS) levels were detected using the fluorescent probe DCFH-DA (green fluorescence). The boxed area in the right panel indicates a magnified view under high magnification. **d.** Under BLM (1 μg/ml) stimulation, AMs were treated with si_PRDX4 or Conoidin A (Con A) for 48 hours. Non-reducing SDS-PAGE was used to assess the protein expression of PRDX4 (monomer, dimer, oligomer) and PTEN (monomer, dimer). **e.** MLE12 and A549 cells were stimulated with CS (50 μg/cm²) for 48 hours, followed by analysis of PRDX4 protein expression (monomer, dimer, oligomer) using non-reducing SDS-PAGE. **f**, **g** After treatment with H_2_O_2_ (100 μM) for 30 minutes, 1 μg recombinant active PRDX4 protein was detected under non-reducing (**f**) and reducing (**g**) conditions using Coomassie Blue staining for the expression of PRDX4 (monomer, dimer, HWM oligomer). **h** Following a 30-minute treatment with H_2_O_2_ (100 μM), 1 μg recombinant active PRDX4 protein had H_2_O_2_ removed and was then incubated with 20 mM Con A (a PRDX4 protease inhibitor) at 37 °C for 30 minutes, after which PRDX4 expression (monomer, dimer, HWM oligomer) was detected using Coomassie Blue staining.

Data are presented as mean ± standard error of the mean (Mean ± SEM). Statistical significance was determined using

One-Way ANOVA (**a**, **b**) for all experiments. *P ≤ 0.05; **P ≤ 0.01; ***P ≤ 0.001.


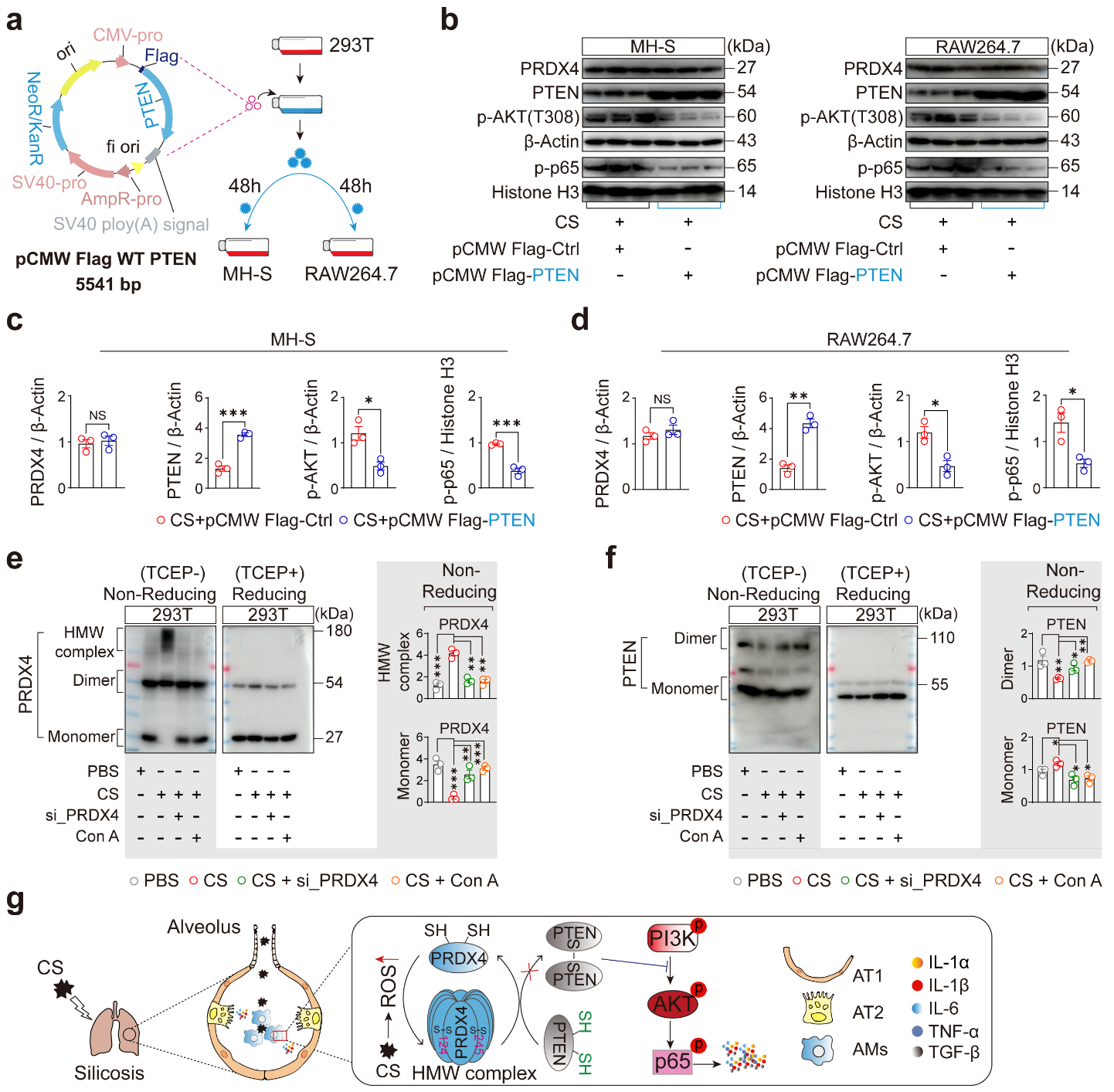


***Figure S6. Impact of PRDX4 oligomers on PTEN dimer formation in 293T Cells***

**a** Workflow diagram of transfection of pCMV-Flag WT PTEN plasmid into 293T cells and infection of MH-S and RAW264.7 cells. **b** After overexpression of PTEN in MH-S and RAW264.7 cells, cells were stimulated with CS (50 μg/cm²) for 48 h; expression levels of cytoplasmic PRDX4, PTEN, p-AKT, and nuclear p-p65 proteins were then assessed. **c, d** Quantitative analysis of protein expression in MH-S (c) and RAW264.7 (d) cells. **e**, **f** Non-Reducing and Reducing SDS-PAGE analyses of PRDX4 (monomer, dimer, multimer) (**e**) and PTEN (**f**) (monomer, dimer) protein expression in 293T cells treated with si_PRDX4 and Con A (10 μM) after CS (50 μg/cm^2^) stimulation. The right column shows the quantitative analysis results of PRDX4 and PTEN proteins, with the experiment repeated three times. **g** Schematic illustration of PRDX4 activating the AKT/NF-κB signaling pathway by inhibiting the formation of PTEN homodimers.

Data are presented as mean ± standard error of the mean (Mean ± SEM). Statistical significance and P values were determined using a two-tailed unpaired t-test (**c**, **d**) and One-Way ANOVA (**e**, **f**). Non-Reducing SDS-PAGE was performed without any thiol-reducing reagents (including TCEP, DTT, β-mercaptoethanol), with the protein heating condition being 70 °C for 10 minutes (**e**, **f**). In Reducing SDS-PAGE, TCEP (5 mM) was added and incubated for 30 minutes before heating at 100 °C for 10 minutes (**e**, **f**). NS, not significant; *P ≤ 0.05; **P ≤ 0.01; ***P ≤ 0.001.


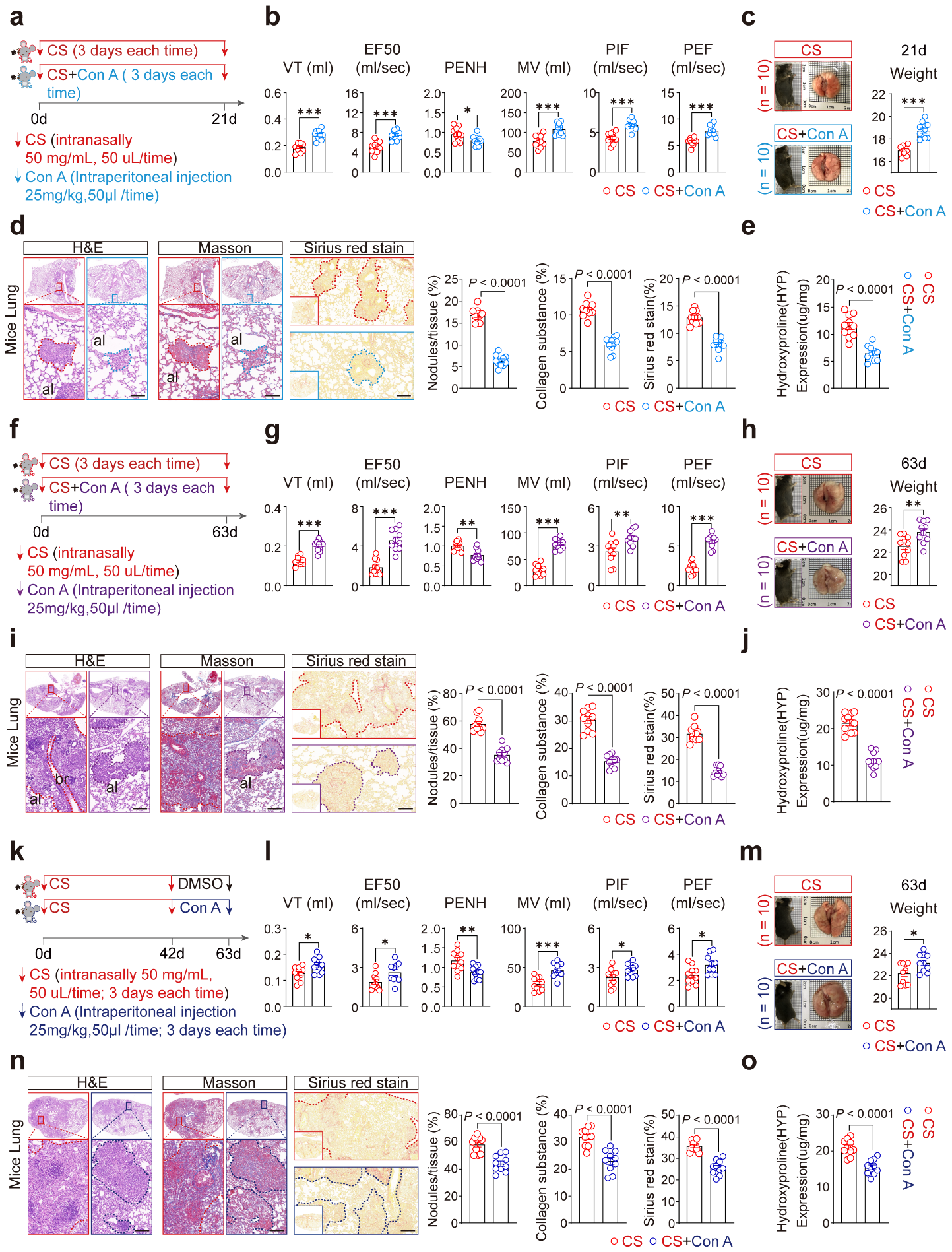


***Figure S7. Conoidin A (Con A) inhibits pulmonary fibrosis in silicosis mice during both the inflammatory phase (day 21) and fibrotic phase (day 63).***

**a** Schematic diagram of the Con A treatment regimen in the inflammatory phase (day 21) silicosis mouse model, including two groups: CS-21 (n=10) and CS-21 + Con A-21 (n=10). **b** Lung function data for mice in the CS-21 and CS-21 + Con A-21 groups. **c** Gross lung appearance and body weight data at day 21 for mice in the CS-21 and CS-21 + Con A-21 groups. **d**. Representative images of lung tissue sections stained with HE, Masson’s trichrome, and Sirius Red from CS-21 and CS-21 + Con A-21 groups, with inset panels showing high-magnification views of selected areas. Dashed lines indicate lung nodules and collagen deposition regions. Statistical analyses of relative lung nodule area and collagen content are shown on the right; each data point represents an individual mouse. **e** Quantification of hydroxyproline (HYP) content in lung tissues from CS-21 and CS-21 + Con A-21 groups; each data point corresponds to a single mouse. **f** Schematic diagram of Con A treatment in the fibrotic phase (day 63) silicosis mouse model, including two groups: CS-63 (n=10) and CS-63 + Con A-63 (n=10). **g** Lung function data for mice in the CS-63 and CS-63 + Con A-63 groups. **h** Gross lung appearance and body weight data at day 63 for mice in the CS-63 and CS-63 + Con A-63 groups. **i** Representative HE, Masson’s, and Sirius Red staining of lung sections from CS-63 and CS-63 + Con A-63 groups with high-magnification insets highlighting selected regions; dashed lines mark lung nodules and collagen deposition. Statistical analysis of relative nodule area and collagen content is presented on the right; each data point represents one mouse. **j** Hydroxyproline (HYP) content measurement in lung tissues from CS-63 and CS-63 + Con A-63 groups; each point represents an individual mouse. **k** Schematic of the Con A treatment model initiated after fibrosis formation (day 42) in silicosis mice, including two groups: CS-42 + DMSO-21 (n=10) and CS-42 + Con A-21 (n=10). **l** Lung function data for CS-42 + DMSO-21 and CS-42 + Con A-21 groups. **m** Gross lung appearance and body weight at day 63 for CS-42 + DMSO-21 and CS-42 + Con A-21 groups. **n** HE, Masson’s, and Sirius Red staining of lung tissues from CS-42 + DMSO-21 and CS-42 + Con A-21 groups with high-magnification insets of selected areas. Dashed lines indicate lung nodules and collagen deposits. Statistical analyses of relative nodule area and collagen content are shown; each data point represents an individual mouse. **o** Hydroxyproline (HYP) content quantification in lung tissues from CS-42 + DMSO-21 and CS-42 + Con A-21 groups; each data point represents one mouse.

Scale bars: 100 µm (**d**, **i**, **n**), 1000 µm (**c**, **h**, **m**). Abbreviations: al, alveoli; br, bronchi. Data are presented as mean ± standard error of the mean (Mean ± SEM). Statistical significance was determined using two-tailed unpaired t-tests (**b**, **c**, **d**, **e**, **g**, **h**, **i**, **j**, **i**, **m**, **n**, **o**). *P ≤ 0.05; **P ≤ 0.01; ***P ≤ 0.001.

***Supplementary Tables S1-S6***

***Supplementary Table S1: Inflammatory and Fibrosis Genes.***

| **Inflammatory Genes** | | | | | | | | | | | | | | |
| --- | --- | --- | --- | --- | --- | --- | --- | --- | --- | --- | --- | --- | --- | --- |
| **BIOCARTA_INFLAM_PATHWAY** | | **GOBP_ACUTE_INFLAMMATORY_RESPONSE** | | | | | | | | | | **GOBP_CYTOKINE_PRODUCTION_INVOLVED_IN_INFLAMMATORY_RESPONSE** | | |
| **> Cytokines and Inflammatory Response** | | **> Inflammation which comprises a rapid, short-lived, relatively uniform response to acute injury or antigenic challenge and is characterized by accumulations of fluid, plasma proteins, and granulocytic leukocytes. An acute inflammatory response occurs within a matter of minutes or hours, and either resolves within a few days or becomes a chronic inflammatory response. [GO_REF:0000022, GOC:add, ISBN:0781735149]** | | | | | | | | | | **> The synthesis or release of a cytokine following a inflammatory stimulus as part of an inflammatory response, resulting in an increase in its intracellular or extracellular levels. [GOC:add, ISBN:0781735149]** | | |
| CD4 | | ACVR1 | | | GATA3 | | PPARG | | | ABCD1 | | MIR197 | | |
| CSF1 | | ADAM8 | | | GSTP1 | | PRCP | | | ABCD2 | | MIR203A | | |
| CSF2 | | ADCYAP1 | | | HAMP | | PTGER3 | | | ADCY7 | | MIR21 | | |
| CSF3 | | ADORA1 | | | HFE | | PTGES | | | ALOX5 | | MIR221 | | |
| CXCL8 | | AHSG | | | HLA-E | | PTGS2 | | | APOD | | MIR222 | | |
| HLA-DRA | | ANO6 | | | HP | | REG3A | | | APPL1 | | MIR26A1 | | |
| HLA-DRB1 | | APCS | | | HPR | | REG3G | | | APPL2 | | MIR338 | | |
| HLA-DRB3 | | APOL2 | | | ICAM1 | | RHBDD3 | | | BAP1 | | MIR378A | | |
| HLA-DRB4 | | ASH1L | | | IL1A | | S100A8 | | | CD6 | | MIR675 | | |
| HLA-DRB5 | | ASS1 | | | IL1B | | SAA1 | | | CHID1 | | MIR93 | | |
| IFNA1 | | B4GALT1 | | | IL20RB | | SAA2 | | | CLEC7A | | MIR98 | | |
| IFNB1 | | BTK | | | IL22 | | SAA4 | | | CUEDC2 | | MYD88 | | |
| IFNG | | C2CD4A | | | IL31RA | | SELENOS | | | F2 | | NLRC3 | | |
| IL10 | | C2CD4B | | | IL4 | | SERPINA1 | | | GBP5 | | NLRP7 | | |
| IL11 | | C3 | | | IL6 | | SERPINA3 | | | GPSM3 | | NOD2 | | |
| IL13 | | CCR7 | | | IL6R | | SERPINC1 | | | H19 | | NOS2 | | |
| IL15 | | CD163 | | | IL6ST | | SERPINF2 | | | HIF1A | | PDCD4 | | |
| IL1A | | CD6 | | | INS | | SIGIRR | | | IL17A | | PER1 | | |
| IL1B | | CEBPB | | | ITIH4 | | TAC1 | | | IL17B | | PLA2G3 | | |
| IL2 | | CNR1 | | | KLKB1 | | TFR2 | | | IL17D | | PLD3 | | |
| IL3 | | CREB3L3 | | | LBP | | TNF | | | IL17F | | PLD4 | | |
| IL4 | | CRP | | | MBL2 | | TNFRSF11A | | | IL17RC | | PPARA | | |
| IL5 | | CTNNBIP1 | | | MIR92A1 | | TNFSF11 | | | IL1R2 | | PYCARD | | |
| IL6 | | DNASE1 | | | MRGPRX1 | | TNFSF4 | | | IL6 | | SIRPA | | |
| IL7 | | DNASE1L3 | | | MYLK3 | | TREM1 | | | KPNA6 | | STAT3 | | |
| PDGFA | | EDNRB | | | NLRP3 | | UGT1A1 | | | LEP | | TICAM1 | | |
| TGFB1 | | EIF2AK1 | | | NPFF | | VCAM1 | | | LILRB4 | | TLR4 | | |
| TGFB2 | | ELANE | | | NPY5R | | VNN1 | | | MACIR | | TLR6 | | |
| TGFB3 | | EPO | | | NUPR1 | | ZP3 | | | MAPK14 | | TNF | | |
| ICAM1 | | EXT1 | | | OGG1 | |  | | | MEFV | | ZC3H12A | | |
| ITGA4 | | F12 | | | OPRM1 | |  | | | MIR125A | |  | | |
| ITGAL | | F2 | | | ORM1 | |  | | | MIR129-1 | |  | | |
| ITGB1 | | F3 | | | ORM2 | |  | | | MIR135A1 | |  | | |
| ITGB2 | | F8 | | | OSM | |  | | | MIR136 | |  | | |
| KNG1 | | FCGR2B | | | OSMR | |  | | | MIR140 | |  | | |
| SELP | | FFAR2 | | | PARK7 | |  | | | MIR146A | |  | | |
| SELPLG | | FFAR3 | | | PIK3CG | |  | | | MIR152 | |  | | |
| TNF | | FN1 | | | PLA2G2D | |  | | | MIR16-1 | |  | | |
| VCAM1 | | FUT7 | | | PLSCR1 | |  | | | MIR17 | |  | | |
| **Fibrosis Genes** | | | | | | | | | | | | | | |
| **HALLMARK_EPITHELIAL_MESENCHYMAL_TRANSITION** | | | | | | | | | **HP_PULMONARY_FIBROSIS** | | | | **WP_LUNG_FIBROSIS** | |
| **> Genes defining epithelial-mesenchymal transition, as in wound healing, fibrosis and metastasis.** | | | | | | | | | **> Pulmonary fibrosis** | | | | **> Lung fibrosis** | |
| ABI3BP | COPA | | GEM | MFAP5 | | SCG2 | | VEGFA | ABCA3 | | NKX2-1 | | ATP11A | MMP2 |
| ACTA2 | CRLF1 | | GJA1 | MGP | | SDC1 | | VEGFC | AP3B1 | | NOP10 | | BMP7 | MMP9 |
| ADAM12 | CTHRC1 | | GLIPR1 | MMP1 | | SDC4 | | VIM | ATP11A | | NR5A1 | | CALCA | MT2A |
| ANPEP | CXCL1 | | GPC1 | MMP14 | | SERPINE1 | | WIPF1 | BACH2 | | NUP107 | | CCL11 | MUC5B |
| APLP1 | CXCL12 | | GPX7 | MMP2 | | SERPINE2 | | WNT5A | BMP15 | | PARN | | CCL2 | NFE2L2 |
| AREG | CXCL6 | | GREM1 | MMP3 | | SERPINH1 | |  | BNC1 | | PLEC | | CCL3 | PARN |
| BASP1 | CXCL8 | | HTRA1 | MSX1 | | SFRP1 | |  | BTNL2 | | POLR3H | | CCL4 | PDGFA |
| BDNF | DAB2 | | ID2 | MXRA5 | | SFRP4 | |  | CASP10 | | PRKCD | | CCL5 | PDGFB |
| BGN | D CN | | IGFBP2 | MYL9 | | SGCB | |  | CAV1 | | PRTN3 | | CCN2 | PLAU |
| BMP1 | DKK1 | | IGFBP3 | MYLK | | SGCD | |  | CCN2 | | PSMC3IP | | CCR2 | PTX3 |
| CADM1 | DPYSL3 | | IGFBP4 | NID2 | | SGCG | |  | CCR6 | | PTPN22 | | CCR3 | RTEL1 |
| CALD1 | DST | | IL15 | NNMT | | SLC6A8 | |  | CFTR | | RAC2 | | CEBPB | SERPINA1 |
| CALU | ECM1 | | IL32 | NOTCH2 | | SLIT2 | |  | CLCA4 | | RASGRP1 | | CMA1 | SFTPA1 |
| CAP2 | ECM2 | | IL6 | NT5E | | SLIT3 | |  | CTLA4 | | RCBTB1 | | CSF2 | SFTPA2 |
| CAPG | EDIL3 | | INHBA | NTM | | SNAI2 | |  | CYBC1 | | RNF168 | | CSF3 | SFTPC |
| CCN1 | EFEMP2 | | ITGA2 | OXTR | | SNTB1 | |  | DCTN4 | | RTEL1 | | CXCL2 | SKIL |
| CCN2 | ELN | | ITGA5 | P3H1 | | SPARC | |  | DKC1 | | SFTPA1 | | CXCL8 | SMAD7 |
| CD44 | EMP3 | | ITGAV | PCOLCE | | SPOCK1 | |  | DPP9 | | SFTPA2 | | CYSLTR2 | SPP1 |
| CD59 | ENO2 | | ITGB1 | PCOLCE2 | | SPP1 | |  | DSP | | SFTPC | | DPP9 | STN1 |
| CDH11 | FAP | | ITGB3 | PDGFRB | | TAGLN | |  | EHHADH | | SLC34A1 | | DSP | TERT |
| CDH2 | FAS | | ITGB5 | PDLIM4 | | TFPI2 | |  | FAM111B | | SP110 | | EDN1 | TGFA |
| CDH6 | FBLN1 | | JUN | PFN2 | | TGFB1 | |  | FAM13A | | SPIDR | | EGF | TGFB1 |
| COL11A1 | FBLN2 | | LAMA1 | PLAUR | | TGFBI | |  | FAS | | STING1 | | ELMOD2 | TIMP1 |
| COL12A1 | FBLN5 | | LAMA2 | PLOD1 | | TGFBR3 | |  | FASLG | | STN1 | | ELN | TNF |
| COL3A1 | FBN1 | | LAMA3 | PLOD2 | | TGM2 | |  | FSHR | | STX1A | | FAM13A |  |
| COL1A1 | FBN2 | | LAMC1 | PLOD3 | | THBS1 | |  | GATM | | TERC | | FGF1 |  |
| COL1A2 | FERMT2 | | LAMC2 | PMEPA1 | | THBS2 | |  | GBA | | TERT | | FGF2 |  |
| COL3A1 | FGF2 | | LGALS1 | PMP22 | | THY1 | |  | HLA-DPA1 | | TGFB1 | | FGF7 |  |
| COL4A1 | FLNA | | LOX | POSTN | | TIMP1 | |  | HLA-DPB1 | | THOC2 | | GREM1 |  |
| COL4A2 | FMOD | | LOXL1 | PPIB | | TIMP3 | |  | HLA-DRB1 | | TINF2 | | HGF |  |
| COL5A1 | FN1 | | LOXL2 | PRRX1 | | TNC | |  | HPS1 | | ZCCHC8 | | HMOX1 |  |
| COL5A2 | FOXC2 | | LRP1 | PRSS2 | | TNFAIP3 | |  | HPS4 | |  | | IGF1 |  |
| COL5A3 | FSTL1 | | LRRC15 | PTHLH | | TNFRSF11B | |  | IL1RN | |  | | IL12B |  |
| COL6A2 | FSTL3 | | LUM | PTX3 | | TNFRSF12A | |  | IRF5 | |  | | IL13 |  |
| COL6A3 | FUCA1 | | MAGEE1 | PVR | | TPM1 | |  | KIAA0319L | |  | | IL1B |  |
| COL7A1 | FZD8 | | MATN2 | QSOX1 | | TPM2 | |  | MRPS22 | |  | | IL4 |  |
| COL8A2 | GADD45A | | MATN3 | RGS4 | | TPM4 | |  | MUC5B | |  | | IL5 |  |
| COLGALT1 | GADD45B | | MCM7 | RHOB | | VCAM1 | |  | NDUFAF6 | |  | | IL6 |  |
| COMP | GAS1 | | MEST | SAT1 | | VCAN | |  | NHP2 | |  | | MECP2 |  |

***Supplementary Table S2: Differential Expression of Inflammatory Genes in Human Lung Tissue.***

| **Human Diff-Inflammatory Genes** | | | | | | | | | | | | | | | | | |
| --- | --- | --- | --- | --- | --- | --- | --- | --- | --- | --- | --- | --- | --- | --- | --- | --- | --- |
| ID | | Normol-1 | | Normol-2 | | | Normol-3 | | Normol-4 | | Normol-5 | | | Normol-6 | | Normol-7 | |
| **PRDX4** | | 3.949 | | 4.96 | | | 6.68 | | 7.112 | | 9.007 | | | 4.524 | | 1.983 | |
| ACVR1 | | 3.24 | | 2.81 | | | 3.22 | | 4.07 | | 4.34 | | | 2.22 | | 1.49 | |
| ADCYAP1 | | 0.03 | | 0.01 | | | 0.01 | | 0.00 | | 0.08 | | | 0.13 | | 0.01 | |
| ALOX5 | | 18.82 | | 30.24 | | | 26.63 | | 18.87 | | 64.02 | | | 32.70 | | 69.93 | |
| B4GALT1 | | 33.40 | | 10.83 | | | 12.22 | | 14.43 | | 11.38 | | | 7.10 | | 5.98 | |
| CD163 | | 4.83 | | 19.96 | | | 3.64 | | 1.68 | | 13.82 | | | 5.36 | | 16.20 | |
| EPO | | 0.11 | | 0.15 | | | 0.26 | | 0.00 | | 0.10 | | | 0.16 | | 0.43 | |
| FCGR2B | | 0.89 | | 0.88 | | | 0.96 | | 0.48 | | 1.78 | | | 1.73 | | 0.58 | |
| FN1 | | 42.48 | | 38.51 | | | 47.30 | | 36.79 | | 54.40 | | | 21.97 | | 12.02 | |
| GBP5 | | 1.55 | | 0.67 | | | 0.57 | | 0.49 | | 1.10 | | | 1.02 | | 0.56 | |
| HAMP | | 0.21 | | 0.27 | | | 0.04 | | 0.34 | | 0.10 | | | 0.38 | | 0.19 | |
| HFE | | 0.77 | | 1.37 | | | 1.11 | | 0.58 | | 1.29 | | | 2.58 | | 1.54 | |
| HIF1A | | 15.95 | | 5.64 | | | 5.17 | | 13.01 | | 10.15 | | | 8.55 | | 5.21 | |
| HLA-E | | 489.13 | | 283.67 | | | 306.13 | | 368.22 | | 237.09 | | | 257.87 | | 135.43 | |
| IFNG | | 0.23 | | 0.04 | | | 0.00 | | 0.00 | | 0.08 | | | 0.44 | | 0.00 | |
| IL10 | | 0.54 | | 0.36 | | | 0.05 | | 0.34 | | 0.22 | | | 0.35 | | 0.58 | |
| IL11 | | 0.69 | | 0.08 | | | 0.18 | | 0.00 | | 0.07 | | | 0.09 | | 0.05 | |
| IL1A | | 2.92 | | 0.43 | | | 0.76 | | 1.21 | | 1.54 | | | 1.29 | | 1.27 | |
| ITGB1 | | 21.14 | | 16.23 | | | 8.47 | | 26.37 | | 45.19 | | | 21.56 | | 22.10 | |
| ITGB2 | | 8.22 | | 11.30 | | | 11.21 | | 8.39 | | 15.93 | | | 14.62 | | 6.45 | |
| ITIH4 | | 0.36 | | 0.36 | | | 0.23 | | 0.30 | | 0.21 | | | 0.51 | | 0.92 | |
| KLKB1 | | 1.14 | | 1.19 | | | 3.34 | | 1.07 | | 0.85 | | | 1.87 | | 1.82 | |
| LEP | | 0.00 | | 0.00 | | | 0.00 | | 0.02 | | 0.00 | | | 0.03 | | 0.03 | |
| LILRB4 | | 1.03 | | 2.23 | | | 0.86 | | 2.41 | | 1.98 | | | 3.70 | | 1.08 | |
| MYLK3 | | 0.14 | | 0.04 | | | 0.72 | | 0.10 | | 0.11 | | | 0.11 | | 0.06 | |
| OSMR | | 11.56 | | 4.67 | | | 3.10 | | 2.88 | | 3.90 | | | 3.75 | | 2.69 | |
| PLA2G3 | | 0.37 | | 1.05 | | | 1.44 | | 0.52 | | 1.31 | | | 2.18 | | 2.96 | |
| PLD3 | | 36.56 | | 66.30 | | | 68.21 | | 47.38 | | 63.13 | | | 83.52 | | 38.74 | |
| PRCP | | 3.33 | | 2.19 | | | 1.78 | | 3.68 | | 3.70 | | | 2.09 | | 1.10 | |
| PTGS2 | | 14.82 | | 1.18 | | | 0.59 | | 1.80 | | 1.24 | | | 0.86 | | 0.46 | |
| SAA1 | | 1.53 | | 53.84 | | | 34.95 | | 1.67 | | 10.43 | | | 4.83 | | 49.63 | |
| SELP | | 2.76 | | 1.22 | | | 2.76 | | 2.49 | | 3.37 | | | 3.69 | | 1.65 | |
| SIGIRR | | 24.79 | | 21.68 | | | 38.82 | | 15.15 | | 13.06 | | | 32.33 | | 16.70 | |
| TNFSF11 | | 0.00 | | 0.00 | | | 0.00 | | 0.02 | | 0.01 | | | 0.02 | | 0.00 | |
| UGT1A1 | | 0.13 | | 0.06 | | | 0.44 | | 0.00 | | 0.01 | | | 0.00 | | 0.01 | |
| VCAM1 | | 1.12 | | 0.09 | | | 0.05 | | 0.22 | | 0.19 | | | 0.52 | | 0.08 | |
| VNN1 | | 0.80 | | 1.64 | | | 2.13 | | 1.78 | | 0.17 | | | 0.91 | | 0.66 | |
| ID | Silicosis-1 | | Silicosis-2 | | Silicosis-3 | Silicosis-4 | | Silicosis-5 | | Silicosis-6 | | Silicosis-7 | Silicosis-8 | | Silicosis-9 | | Silicosis-10 |
| **PRDX4** | 10.17 | | 9.81 | | 5.77 | 5.74 | | 10.63 | | 19.28 | | 11.91 | 22.69 | | 13.26 | | 12.01 |
| ACVR1 | 5.36 | | 5.74 | | 3.93 | 5.46 | | 5.28 | | 5.91 | | 4.66 | 5.60 | | 4.06 | | 3.32 |
| ADCYAP1 | 0.30 | | 1.05 | | 0.05 | 0.01 | | 0.36 | | 0.27 | | 0.43 | 0.27 | | 0.12 | | 2.49 |
| ALOX5 | 10.03 | | 22.01 | | 9.16 | 8.63 | | 11.61 | | 14.00 | | 14.37 | 19.89 | | 8.70 | | 3.66 |
| B4GALT1 | 17.38 | | 22.61 | | 23.64 | 18.47 | | 19.09 | | 26.73 | | 18.18 | 18.26 | | 13.12 | | 53.86 |
| CD163 | 29.20 | | 30.73 | | 11.11 | 16.04 | | 45.86 | | 39.83 | | 17.18 | 28.39 | | 42.79 | | 6.07 |
| EPO | 0.02 | | 0.14 | | 0.03 | 0.08 | | 0.08 | | 0.04 | | 0.07 | 0.07 | | 0.00 | | 0.00 |
| FCGR2B | 6.01 | | 15.12 | | 1.80 | 1.43 | | 6.55 | | 8.26 | | 5.90 | 16.16 | | 4.56 | | 1.64 |
| FN1 | 57.76 | | 63.68 | | 89.37 | 39.17 | | 62.11 | | 54.47 | | 66.61 | 59.76 | | 76.62 | | 32.01 |
| GBP5 | 1.03 | | 2.06 | | 2.24 | 1.66 | | 1.23 | | 0.82 | | 2.71 | 5.83 | | 1.34 | | 0.49 |
| HAMP | 0.99 | | 3.20 | | 0.08 | 0.16 | | 0.61 | | 2.86 | | 0.91 | 3.00 | | 0.22 | | 0.18 |
| HFE | 2.71 | | 4.06 | | 1.49 | 1.38 | | 1.97 | | 2.81 | | 2.06 | 2.41 | | 2.98 | | 1.47 |
| HIF1A | 13.16 | | 15.39 | | 10.84 | 9.65 | | 14.42 | | 39.68 | | 8.37 | 18.29 | | 16.37 | | 48.10 |
| HLA-E | 348.35 | | 393.75 | | 561.50 | 784.87 | | 492.63 | | 257.19 | | 572.04 | 473.53 | | 208.84 | | 327.89 |
| IFNG | 0.36 | | 0.22 | | 0.20 | 0.30 | | 0.17 | | 0.28 | | 0.74 | 2.74 | | 0.80 | | 0.40 |
| IL10 | 0.69 | | 0.79 | | 0.22 | 0.21 | | 0.97 | | 4.35 | | 0.61 | 2.41 | | 1.98 | | 1.30 |
| IL11 | 0.33 | | 0.51 | | 0.38 | 0.42 | | 0.56 | | 0.98 | | 0.30 | 0.08 | | 0.05 | | 1.51 |
| IL1A | 0.14 | | 0.04 | | 0.14 | 0.08 | | 0.21 | | 0.35 | | 0.14 | 0.77 | | 0.09 | | 0.76 |
| ITGB1 | 20.48 | | 19.07 | | 45.86 | 39.32 | | 68.60 | | 63.30 | | 75.69 | 52.10 | | 55.22 | | 69.05 |
| ITGB2 | 40.17 | | 68.00 | | 13.40 | 12.18 | | 28.75 | | 42.73 | | 26.40 | 54.19 | | 16.48 | | 6.18 |
| ITIH4 | 0.11 | | 0.21 | | 0.11 | 0.14 | | 0.09 | | 0.06 | | 0.11 | 0.15 | | 0.19 | | 0.07 |
| KLKB1 | 0.51 | | 0.84 | | 1.14 | 0.95 | | 0.74 | | 0.26 | | 1.49 | 0.58 | | 0.66 | | 0.48 |
| LEP | 0.02 | | 0.00 | | 0.02 | 0.04 | | 0.04 | | 1.27 | | 0.01 | 0.01 | | 1.22 | | 0.04 |
| LILRB4 | 9.35 | | 23.84 | | 1.48 | 1.79 | | 10.02 | | 15.87 | | 5.94 | 13.81 | | 8.55 | | 1.55 |
| MYLK3 | 0.02 | | 0.01 | | 0.02 | 0.02 | | 0.03 | | 0.01 | | 0.03 | 0.05 | | 0.02 | | 0.01 |
| OSMR | 6.13 | | 6.03 | | 13.62 | 9.39 | | 9.92 | | 10.48 | | 4.46 | 3.60 | | 6.12 | | 16.57 |
| PLA2G3 | 0.44 | | 0.10 | | 0.49 | 0.64 | | 0.36 | | 0.47 | | 0.16 | 1.02 | | 0.00 | | 0.29 |
| PLD3 | 206.11 | | 275.41 | | 43.73 | 68.08 | | 133.14 | | 154.10 | | 84.02 | 172.60 | | 59.66 | | 40.42 |
| PRCP | 7.00 | | 8.89 | | 5.75 | 4.99 | | 9.01 | | 11.25 | | 9.97 | 13.35 | | 9.02 | | 5.50 |
| PTGS2 | 2.71 | | 11.02 | | 8.74 | 5.62 | | 2.38 | | 6.79 | | 4.25 | 6.89 | | 0.58 | | 10.42 |
| SAA1 | 23.17 | | 1.26 | | 1.39 | 2.65 | | 13.30 | | 6.17 | | 2.34 | 6.23 | | 3.99 | | 4.62 |
| SELP | 8.88 | | 3.87 | | 6.31 | 2.51 | | 6.89 | | 6.75 | | 12.77 | 2.61 | | 6.31 | | 6.25 |
| SIGIRR | 19.80 | | 16.54 | | 17.57 | 15.83 | | 12.38 | | 9.02 | | 16.56 | 13.78 | | 6.25 | | 8.53 |
| TNFSF11 | 0.05 | | 0.03 | | 0.02 | 0.01 | | 0.02 | | 0.32 | | 0.02 | 0.07 | | 0.05 | | 0.06 |
| UGT1A1 | 0.00 | | 0.01 | | 0.00 | 0.00 | | 0.01 | | 0.00 | | 0.00 | 0.02 | | 0.00 | | 0.00 |
| VCAM1 | 2.34 | | 1.78 | | 0.76 | 0.28 | | 1.32 | | 6.42 | | 2.28 | 4.97 | | 4.00 | | 30.81 |
| VNN1 | 4.58 | | 9.91 | | 0.61 | 1.20 | | 3.38 | | 6.83 | | 2.93 | 5.17 | | 5.35 | | 0.60 |

***Supplementary Table S3: Differential Expression of Inflammatory Genes in Mouse Lung Tissue.***

| **Mouse Diff-Inflammatory Genes** | | | | | | | | | |
| --- | --- | --- | --- | --- | --- | --- | --- | --- | --- |
| ID | con-1 | con-2 | s3-1 | s3-2 | s3-3 | s6-1 | s6-2 | s6-3 | s9-1 |
| **Prdx1** | 51.59 | 16.88 | 68.12 | 76.66 | 72.72 | 157.34 | 129.26 | 139.38 | 563.15 |
| **Prdx3** | 18.52 | 8.42 | 24.99 | 30.88 | 29.76 | 38.41 | 41.36 | 47.82 | 101.51 |
| **Prdx6** | 89.61 | 47.93 | 180.26 | 208.09 | 221.51 | 216.24 | 286.25 | 297.46 | 421.67 |
| Abcd1 | 5.96 | 2.76 | 9.5 | 7.95 | 8.84 | 10.86 | 11.49 | 11.06 | 11.62 |
| Acvr1 | 4.24 | 4.05 | 7.08 | 7.21 | 7.49 | 7.17 | 7.78 | 6.59 | 6.54 |
| Adam8 | 3.16 | 2.78 | 13.08 | 11.79 | 12.8 | 19.34 | 12.83 | 8.17 | 10.9 |
| Adcy7 | 13 | 6.08 | 23.96 | 22.04 | 21.05 | 22.12 | 26.38 | 26.53 | 28.24 |
| Adcyap1 | 1 | 2.29 | 0 | 0 | 0 | 0.02 | 0 | 0.02 | 0 |
| Ahsg | 3.49 | 1.58 | 0.08 | 0.33 | 0.11 | 0.19 | 0.25 | 0.34 | 0.64 |
| Ano6 | 7.41 | 6.91 | 13.12 | 12.26 | 12.62 | 11.81 | 15.76 | 13.46 | 11.55 |
| Apod | 1.4 | 2.98 | 0.71 | 0.78 | 0.84 | 1.36 | 0.57 | 1.79 | 1.92 |
| Appl1 | 7.79 | 4.88 | 11.27 | 13.5 | 11.02 | 10.71 | 11.09 | 12.53 | 8.26 |
| Appl2 | 15.5 | 8.9 | 22.38 | 26.83 | 22.01 | 22.15 | 15.77 | 18.36 | 16.2 |
| Ash1l | 12.54 | 8.98 | 14.28 | 15.23 | 14.14 | 12.59 | 13.11 | 13.23 | 11.24 |
| B4galt1 | 17.18 | 9.84 | 44.85 | 44.34 | 44.13 | 56.92 | 57.84 | 44.84 | 59.16 |
| Bap1 | 5.3 | 3.43 | 10.99 | 10.28 | 11.26 | 11.35 | 12.47 | 10.75 | 16.7 |
| Cebpb | 14.8 | 8.56 | 46.07 | 40.72 | 53.57 | 68.4 | 74.66 | 45.99 | 84.17 |
| Chid1 | 2.02 | 2.13 | 3.44 | 3.07 | 3.8 | 3.48 | 4.24 | 3.74 | 6.04 |
| Creb3l3 | 1.04 | 2.02 | 0.07 | 0.06 | 0.03 | 0.03 | 0 | 0.05 | 0.09 |
| Crp | 1.43 | 1.3 | 0.01 | 0 | 0 | 0 | 0 | 0 | 0 |
| Csf1 | 4.79 | 3.01 | 9.19 | 5.39 | 7.85 | 9.97 | 9.31 | 7.88 | 10.3 |
| Csf3 | 0.94 | 1.54 | 0.15 | 0.12 | 0.03 | 0.12 | 0.18 | 0.07 | 0 |
| Cuedc2 | 6.93 | 4.1 | 7.55 | 8.4 | 9.49 | 11.79 | 10.54 | 12.94 | 22.33 |
| Dnase1l3 | 0.92 | 1.88 | 0.65 | 0.23 | 0.26 | 0.66 | 0.28 | 0.41 | 0.21 |
| Ednrb | 15.03 | 11.71 | 5.93 | 9.41 | 7.52 | 3.6 | 7.92 | 4.14 | 4.03 |
| Eif2ak1 | 3.16 | 2.81 | 4.65 | 4.38 | 5.1 | 5.04 | 5.47 | 5.21 | 4.94 |
| Elane | 1.44 | 1.77 | 0 | 0.04 | 0.05 | 0 | 0 | 0 | 0.16 |
| Epo | 1.55 | 2.71 | 0 | 0 | 0 | 0 | 0 | 0.05 | 0.06 |
| Ext1 | 5.15 | 4.26 | 7.74 | 7.69 | 7.81 | 7.91 | 9.35 | 7.27 | 10.2 |
| Fcgr2b | 3.24 | 2.92 | 7.98 | 5.99 | 5.36 | 10.68 | 11.27 | 6.93 | 9.7 |
| Ffar2 | 1.49 | 1.82 | 0.64 | 0.69 | 0.53 | 1.42 | 1.15 | 0.88 | 1.16 |
| Ffar3 | 0.61 | 1.17 | 0.02 | 0 | 0.03 | 0 | 0.02 | 0 | 0.03 |
| Fn1 | 17.5 | 15.54 | 26.77 | 25.38 | 22.05 | 26.52 | 35.18 | 22.47 | 41.88 |
| Gata3 | 2.48 | 2.77 | 1.64 | 0.93 | 0.89 | 0.85 | 1.07 | 0.81 | 0.42 |
| Hamp | 3.46 | 4.94 | 0.43 | 0.21 | 0.47 | 0.1 | 0.2 | 1.83 | 0.66 |
| Hif1a | 10.23 | 6.38 | 18.16 | 19.88 | 16.57 | 25.55 | 25.67 | 21.82 | 36.77 |
| Icam1 | 32.25 | 11.49 | 92.74 | 70.81 | 85.8 | 140.62 | 135.54 | 109.35 | 75.31 |
| Il10 | 1.88 | 3.43 | 0.33 | 0.16 | 0.22 | 0.32 | 0.19 | 0.18 | 0.49 |
| Il11 | 1.18 | 2.88 | 0.26 | 0.19 | 0.19 | 0.19 | 0.35 | 0.28 | 0.27 |
| Il13 | 1.34 | 2.4 | 0.14 | 0.32 | 0.08 | 0.04 | 0.24 | 0.12 | 0.04 |
| Il17a | 0.89 | 2.6 | 0 | 0.04 | 0 | 0.25 | 0.25 | 0 | 0.32 |
| Il17d | 2.01 | 1.59 | 3.59 | 4.02 | 4.86 | 3.31 | 5.58 | 4.94 | 4.98 |
| Il17f | 1.36 | 2.22 | 0 | 0.04 | 0 | 0.02 | 0.08 | 0.07 | 0.16 |
| Il17rc | 5.11 | 3.2 | 10.14 | 9.9 | 12.96 | 11 | 14.5 | 12.45 | 17.76 |
| Il1b | 2.78 | 2.58 | 15.27 | 5.93 | 12.73 | 13.41 | 15.18 | 11.47 | 65.4 |
| Il2 | 1.08 | 1.9 | 0.19 | 0.08 | 0.13 | 0.11 | 0.15 | 0.25 | 0.38 |
| Il20rb | 1.22 | 2 | 1.03 | 0.86 | 0.86 | 1.08 | 0.83 | 0.64 | 0.64 |
| Il31ra | 1.72 | 2.81 | 0.09 | 0.16 | 0.1 | 0.32 | 0.18 | 0.12 | 0.09 |
| Il5 | 1.05 | 0.75 | 0.14 | 0.11 | 0.12 | 0.08 | 0.05 | 0.09 | 0.03 |
| Il6 | 1.29 | 2.68 | 0.65 | 0.5 | 0.36 | 0.7 | 1.28 | 0.75 | 1.24 |
| Il6st | 24.76 | 15.9 | 51.16 | 58.85 | 47.85 | 57.53 | 60.51 | 50.79 | 60.79 |
| Il7 | 1.91 | 2.73 | 1.66 | 1.57 | 1.37 | 2.06 | 1.7 | 1.63 | 1.24 |
| Itgal | 5.9 | 3.15 | 9.58 | 7.35 | 9.36 | 13.92 | 12.29 | 10.72 | 14.42 |
| Itgb1 | 94.83 | 49.72 | 142.87 | 178.64 | 166.27 | 174.11 | 197.15 | 210.83 | 160.91 |
| Itgb2 | 19.86 | 6.4 | 44.43 | 21.53 | 31.43 | 75.44 | 72.86 | 50.73 | 49.8 |
| Itih4 | 12.57 | 7.92 | 69.56 | 36.47 | 53.64 | 160.99 | 95.5 | 62.6 | 104.01 |
| Kng1 | 1.86 | 1.51 | 0 | 0 | 0.04 | 0.06 | 0.03 | 0.04 | 0.07 |
| Kpna6 | 7.33 | 4.9 | 13.87 | 14.34 | 12.23 | 14.72 | 16.45 | 15.14 | 17.8 |
| Lbp | 4.84 | 4.55 | 13.8 | 15.89 | 12.91 | 21.97 | 19.44 | 13.81 | 24 |
| Lep | 0.65 | 1.21 | 0 | 0.08 | 0.01 | 0 | 0 | 0.03 | 0.02 |
| Mapk14 | 11.92 | 7.54 | 25.96 | 21.7 | 23.13 | 23.9 | 27.19 | 24.44 | 31.78 |
| Mbl2 | 1.98 | 3.29 | 0 | 0 | 0 | 0 | 0 | 0 | 0 |
| Mefv | 1.34 | 2.4 | 0.98 | 0.46 | 1.19 | 1.03 | 0.81 | 0.65 | 2.28 |
| Myd88 | 3.75 | 2.7 | 12.53 | 11.77 | 12.06 | 17.89 | 15.27 | 14.51 | 28 |
| Mylk3 | 2.1 | 3.24 | 0.37 | 0.28 | 0.27 | 0.2 | 0.37 | 0.75 | 0.36 |
| Nlrc3 | 1.74 | 1.95 | 1.23 | 1.17 | 0.94 | 1.06 | 0.95 | 1.12 | 1.56 |
| Nos2 | 1.91 | 2 | 0.61 | 1.45 | 0.93 | 0.78 | 0.69 | 0.64 | 1.32 |
| Npy5r | 0.67 | 1.19 | 0 | 0 | 0 | 0 | 0.03 | 0 | 0 |
| Ogg1 | 2.6 | 2.85 | 3.65 | 4.39 | 5.13 | 4.48 | 4.97 | 5.63 | 7.89 |
| Oprm1 | 1.43 | 2.56 | 0.18 | 0.12 | 0.07 | 0.13 | 0.11 | 0.08 | 0.03 |
| Osmr | 5.41 | 6.4 | 15.37 | 20.72 | 14.44 | 18.19 | 18.72 | 13.36 | 18.38 |
| Pdgfa | 12.02 | 5.66 | 11.86 | 15.84 | 15.95 | 16.39 | 16.22 | 16.67 | 9.66 |
| Per1 | 10.51 | 8.53 | 15.8 | 24.57 | 27.54 | 17.88 | 23.12 | 17.02 | 15.31 |
| Pik3cg | 2.7 | 2.4 | 5.65 | 3.56 | 4.24 | 6.87 | 5.45 | 6 | 9.96 |
| Pla2g2d | 1.22 | 2 | 0.72 | 0.36 | 0.3 | 2.15 | 1.12 | 0.69 | 2.59 |
| Pla2g3 | 1.01 | 1.02 | 0.29 | 0.2 | 0.2 | 0.13 | 0.16 | 0.1 | 0.03 |
| Pld3 | 11.95 | 6.39 | 24.43 | 14.11 | 17.21 | 42.73 | 40.03 | 25.31 | 43.63 |
| Plscr1 | 9.96 | 6.11 | 16.77 | 18.2 | 16.29 | 20.83 | 16.87 | 18.94 | 30.3 |
| Prcp | 5.29 | 3.69 | 13.12 | 12.17 | 11.91 | 14.85 | 15.61 | 16.03 | 28.3 |
| Ptger3 | 1 | 1.45 | 0.79 | 0.46 | 0.59 | 0.79 | 0.76 | 0.52 | 1.27 |
| Ptges | 2.94 | 2.5 | 5.62 | 7.68 | 6.23 | 10.48 | 9.97 | 7.26 | 14.06 |
| Ptgs2 | 5.33 | 3.44 | 8.76 | 10.08 | 10.63 | 7 | 11.02 | 13.91 | 10.58 |
| Pycard | 2.14 | 1.56 | 2.78 | 3.35 | 3.19 | 3.97 | 3.5 | 3.4 | 8.49 |
| Reg3a | 0.8 | 4.74 | 0.05 | 0.05 | 0.06 | 0.1 | 0 | 0.06 | 0 |
| Reg3g | 3.31 | 8.97 | 40.82 | 66.04 | 51.32 | 96.16 | 63.23 | 43.65 | 119.74 |
| S100a8 | 13.1 | 10.43 | 54.79 | 22.99 | 33.86 | 59.3 | 73.3 | 132.14 | 3195.18 |
| Saa1 | 0.53 | 0.7 | 0.04 | 0.07 | 0.08 | 0.03 | 0.07 | 0.02 | 0.46 |
| Saa2 | 0.49 | 0.8 | 0.03 | 0.07 | 0 | 0 | 0.05 | 0.02 | 0.02 |
| Saa4 | 1.58 | 2.42 | 0.17 | 0.08 | 0.23 | 0.3 | 0.22 | 0.18 | 0.72 |
| Selenos | 19.17 | 7.94 | 24.33 | 35.53 | 37.6 | 41.27 | 38.52 | 35.73 | 70.31 |
| Selp | 1.5 | 2.53 | 1 | 1.02 | 0.51 | 1.1 | 1.25 | 0.55 | 1.26 |
| Serpinc1 | 1.31 | 1.98 | 0.23 | 0.24 | 0.29 | 0.16 | 0.15 | 0.13 | 0.12 |
| Serpinf2 | 1.59 | 1.93 | 0.23 | 0.11 | 0.1 | 0.04 | 0.13 | 0.22 | 0.16 |
| Sigirr | 3.55 | 2.28 | 5.86 | 5.12 | 6.51 | 5.56 | 6.23 | 5.82 | 8.2 |
| Sirpa | 17.82 | 6.1 | 34.5 | 22.87 | 27.11 | 51.73 | 47.45 | 32.41 | 42.26 |
| Stat3 | 11.16 | 7.32 | 25.77 | 27.43 | 26.66 | 32.22 | 35.46 | 25.25 | 35.06 |
| Tac1 | 1.11 | 2.6 | 0 | 0 | 0 | 0.02 | 0 | 0 | 0 |
| Tfr2 | 1.35 | 1.8 | 0 | 0.03 | 0.02 | 0.1 | 0.04 | 0.09 | 0.2 |
| Tgfb1 | 4.39 | 2.38 | 11.09 | 7.52 | 9.66 | 12.71 | 13.18 | 8.23 | 10.15 |
| Tgfb3 | 3.41 | 2.61 | 5.35 | 7.14 | 5.2 | 3.83 | 6.1 | 8.44 | 3.56 |
| Ticam1 | 2.31 | 1.45 | 3.45 | 4.04 | 4.23 | 4.23 | 4.18 | 2.95 | 5.18 |
| Tlr4 | 6.25 | 3.75 | 16.04 | 13.85 | 12.48 | 23.58 | 22.15 | 23.13 | 32.19 |
| Tlr6 | 1.55 | 1.63 | 1.28 | 0.92 | 0.86 | 1.39 | 1.66 | 1.42 | 2.28 |
| Tnfrsf11a | 2.53 | 3.12 | 4.38 | 4.02 | 4.14 | 5.73 | 5.03 | 4.21 | 4.4 |
| Tnfsf11 | 0.93 | 1.23 | 0.41 | 0.17 | 0.24 | 0.72 | 0.67 | 0.4 | 0.99 |
| Tnfsf4 | 1.34 | 2.51 | 0.17 | 0.09 | 0.08 | 0.18 | 0.18 | 0.31 | 0.18 |
| Trem1 | 1.04 | 1.5 | 2.9 | 1.87 | 2.11 | 3.78 | 3.87 | 2.7 | 6.57 |
| Ugt1a1 | 1.53 | 2.34 | 0.1 | 0.1 | 0.15 | 0.19 | 0.21 | 0.23 | 0.1 |
| Vcam1 | 4.06 | 3.23 | 8.91 | 7.76 | 8.52 | 8.66 | 8.3 | 11.82 | 10.42 |
| Vnn1 | 5.1 | 2.58 | 25.68 | 16.79 | 19.02 | 41.53 | 38.58 | 28.4 | 62.28 |
| Zc3h12a | 2.79 | 1.41 | 7.73 | 5.95 | 6.84 | 13.12 | 9.06 | 6.32 | 12.73 |
| Zp3 | 2.51 | 3.83 | 0.1 | 0 | 0 | 0.03 | 0 | 0 | 0 |

***Supplementary Table S4: Lists of TFs.***

| **Human TRRUST-TFs** | | | |
| --- | --- | --- | --- |
| **# [TF]** | **[Target]** | **[Type]** | **[Reference]** |
| FOS | IL1A | Unknown | [8688489](http://www.ncbi.nlm.nih.gov/pubmed/?term=8688489) |
| [JUN](http://www.genecards.org/index.php?path=/Search/Symbol/JUN) | IL1A | Unknown | [8688489](http://www.ncbi.nlm.nih.gov/pubmed/?term=8688489) |
| [NFKB1](http://www.genecards.org/index.php?path=/Search/Symbol/NFKB1) | IL1A | Repression | [17612514](http://www.ncbi.nlm.nih.gov/pubmed/?term=17612514) |
| [RELA](http://www.genecards.org/index.php?path=/Search/Symbol/RELA) | IL1A | Repression | [17612514](http://www.ncbi.nlm.nih.gov/pubmed/?term=17612514) |
| **# [TF]** | **[Target]** | **[Type]** | **[Reference]** |
| AHR | IL1B | Unknown | 23349129 |
| CEBPB | IL1B | Activation | 17386941 |
| CEBPB | IL1B | Unknown | 10383163;10801783 |
| E2F1 | IL1B | Unknown | 17707233 |
| HMGA1 | IL1B | Unknown | 15901130 |
| HSF1 | IL1B | Repression | 10328874 |
| IRF8 | IL1B | Activation | 17386941 |
| JUN | IL1B | Unknown | 11306276 |
| JUNB | IL1B | Unknown | 11306276 |
| KLF4 | IL1B | Repression | 22449968 |
| NFIL3 | IL1B | Repression | 8547328 |
| NFKB1 | IL1B | Activation | 12686724;14960579;8413223;8679226;9878621 |
| NFKB1 | IL1B | Unknown | 10080875;10963848;16433741;17707233;18285351;20336759;9058643 |
| NFKBIA | IL1B | Repression | 15228586 |
| REL | IL1B | Activation | 8413223 |
| RELA | IL1B | Activation | 12686724;14960579;8021507;8413223;8679226;9878621 |
| RELA | IL1B | Unknown | 10080875;10963848;16433741;17707233;18285351;20336759;9058643 |
| SIRT1 | IL1B | Unknown | 21245135 |
| SPI1 | IL1B | Activation | 17386941 |
| SPI1 | IL1B | Unknown | 10801783 |
| STAT1 | IL1B | Activation | 17386941 |
| SUGP1 | IL1B | Activation | 9878621 |
| YY1 | IL1B | Unknown | 22467534 |
| **# [TF]** | **[Target]** | **[Type]** | **[Reference]** |
| AHR | IL6 | Activation | 20511231 |
| AHR | IL6 | Unknown | 18483242;23349129 |
| ATF4 | IL6 | Unknown | 16931790 |
| CEBPA | IL6 | Unknown | 11975924 |
| CEBPB | IL6 | Activation | 10903137;8725624 |
| CEBPB | IL6 | Unknown | 11120852 |
| CREB1 | IL6 | Activation | 8725624 |
| DDIT3 | IL6 | Activation | 12706815 |
| EGR1 | IL6 | Repression | 15517885 |
| EGR2 | IL6 | Unknown | 24007274 |
| NFKB1 | IL6 | Activation | 10329846;10903137;11327783;11504280;12058956;12887736;14532843;15756023;16984731;17040605;17196927; |
| NFKB1 | IL6 | Repression | 15517885 |
| NFKB1 | IL6 | Unknown | 10823821;10963848;11120852;11975924;15158360;16112536;17490702;17532054;19469019;19707556;2405250;8752656 |
| OTX2 | IL6 | Activation | 21047732 |
| PPARA | IL6 | Unknown | 15001458 |
| RBPJ | IL6 | Activation | 10329846 |
| RBPJ | IL6 | Unknown | 9516466 |
| REL | IL6 | Repression | 1372388 |
| RELA | IL6 | Activation | 10329846;10903137;11327783;11504280;12058956;12887736;14532843;15756023;16984731;17040605;17196927; |
| RELA | IL6 | Repression | 15517885 |
| RELA | IL6 | Unknown | 10542237;10823821;10963848;11120852;11975924;15158360;16112536;17490702;17532054;19469019;19707556; |
| SP1 | IL6 | Activation | 18502099 |
| STAT1 | IL6 | Repression | 18616672 |
| STAT3 | IL6 | Activation | 10391682;18765795;20930550;22547075;22927445 |
| STAT3 | IL6 | Repression | 18616672 |
| STAT3 | IL6 | Unknown | 22105366;22363173 |
| TBP | IL6 | Repression | 9209275 |
| VDR | IL6 | Repression | 11050002 |
| XBP1 | IL6 | Unknown | 16931790 |
| ZFP36 | IL6 | Repression | 21656745 |
| ZMYND1 | IL6 | Activation | 19379743 |
| ZNF300 | IL6 | Activation | 21777376 |
| **# [TF]** | **[Target]** | **[Type]** | **[Reference]** |
| ATF2 | TNF | Activation | 10688670;10748079;10913190;20068037 |
| CEBPB | TNF | Unknown | 10629048;9566900 |
| CEBPD | TNF | Unknown | 10629048 |
| E2F1 | TNF | Unknown | 17707233 |
| EGR1 | TNF | Activation | 10913190;14767560 |
| ETV4 | TNF | Unknown | 7896795 |
| HDAC11 | TNF | Repression | 21239696 |
| HDAC3 | TNF | Unknown | 15356147 |
| HMGB2 | TNF | Activation | 18218727 |
| HSF1 | TNF | Unknown | 18689673 |
| IRF5 | TNF | Unknown | 20237317 |
| JUN | TNF | Activation | 10318823;10688670;10748079;10913190 |
| JUN | TNF | Unknown | 7896795;9566900 |
| LRRFIP1 | TNF | Repression | 16199883 |
| NFAT5 | TNF | Unknown | 11485737 |
| NFKB1 | TNF | Activation | 10079106;10748079;11749970;12058956;12686724;15013838;16707469;16916598;17407192;18057724;20066113;7876168 |
| NFKB1 | TNF | Unknown | 10551799;10837498;10963848;11297551;14960579;15812247;16040075;16433741;17442180;17707233;18285351; |
| NR4A1 | TNF | Repression | 19213954 |
| RELA | TNF | Activation | 10079106;10748079;11749970;12058956;12686724;15013838;16707469;16916598;17407192;18057724;20066113;7876168 |
| RELA | TNF | Unknown | 10551799;10837498;10963848;11297551;14960579;15812247;16040075;16433741;17442180;17707233;18285351; |
| SIRT1 | TNF | Unknown | 21245135 |
| SP1 | TNF | Activation | 10688670;10913190 |
| SP1 | TNF | Repression | 12759366 |
| SPI1 | TNF | Unknown | 10748079 |
| **# [TF]** | **[Target]** | **[Type]** | **[Reference]** |
| ASH1L | TGFB1 | Unknown | 22488473 |
| FOSB | TGFB1 | Unknown | 10843986 |
| FOSL2 | TGFB1 | Unknown | 10843986 |
| JUND | TGFB1 | Unknown | 10843986 |
| NFKB1 | TGFB1 | Unknown | 16365456 |
| SMAD3 | TGFB1 | Repression | 19214138 |
| SMAD4 | TGFB1 | Unknown | 21968601 |
| SMAD7 | TGFB1 | Activation | 19214138 |
| SP1 | TGFB1 | Activation | 9580699 |
| SP1 | TGFB1 | Unknown | 22415074;2808430 |
| STAT3 | TGFB1 | Activation | 16474852 |
| USF1 | TGFB1 | Activation | 14757763 |
| USF2 | TGFB1 | Activation | 14757763 |
| USF2 | TGFB1 | Unknown | 15184388 |
| **Mouse TRRUST-TFs** | | | |
| **# [TF]** | **[Target]** | **[Type]** | **[Reference]** |
| Nfkb1 | Il1a | Activation | 11454276 |
| **# [TF]** | **[Target]** | **[Type]** | **[Reference]** |
| Cebpb | Il1b | Activation | 14670622;16399630;16867259 |
| Egr1 | Il1b | Activation | 12468449 |
| Il17a | Il1b | Activation | 16798734 |
| Irf4 | Il1b | Activation | 10453013 |
| Irf8 | Il1b | Activation | 10453013 |
| Jun | Il1b | Unknown | 17283046 |
| Nfkb1 | Il1b | Activation | 10437651;11454276;14670622;16399630;16867259;24132147 |
| Nfkb1 | Il1b | Unknown | 19915568 |
| Rel | Il1b | Activation | 10437651 |
| Spi1 | Il1b | Activation | 22454293 |
| **# [TF]** | **[Target]** | **[Type]** | **[Reference]** |
| Ahr | Il6 | Repression | 19703987;21930594 |
| Atf3 | Il6 | Repression | 16688168 |
| Atf4 | Il6 | Activation | 23990363 |
| Cebpb | Il6 | Unknown | 1730090 |
| Cebpg | Il6 | Unknown | 12177065 |
| Crebbp | Il6 | Unknown | 10542243;20351184 |
| Ctr9 | Il6 | Unknown | 17911113 |
| Elk1 | Il6 | Activation | 15649404 |
| Ep300 | Il6 | Unknown | 10542243;20351184 |
| Fos | Il6 | Unknown | 20351184 |
| Hdac1 | Il6 | Unknown | 15536134 |
| Jun | Il6 | Activation | 15649404 |
| Jun | Il6 | Unknown | 18490655;20351184 |
| Nfkb1 | Il6 | Activation | 11454276;15241416;18166486;18996370;24132147 |
| Nfkb1 | Il6 | Repression | 19703987 |
| Nfkb1 | Il6 | Unknown | 12672795;18490655 |
| Ppara | Il6 | Unknown | 15001458 |
| Rela | Il6 | Activation | 18996370;22661093 |
| Rela | Il6 | Unknown | 11564778;16166627;20351184 |
| Sirt1 | Il6 | Repression | 23262029 |
| Sp1 | Il6 | Unknown | 15688401 |
| Sp3 | Il6 | Unknown | 15688401 |
| Stat1 | Il6 | Repression | 19703987 |
| Stat3 | Il6 | Activation | 11049111 |
| **# [TF]** | **[Target]** | **[Type]** | **[Reference]** |
| Crebbp | Tnf | Activation | 10760264 |
| Crebbp | Tnf | Unknown | 12509451 |
| Egr1 | Tnf | Activation | 17567466;18651635 |
| Egr1 | Tnf | Unknown | 11739517;11856733;11997234;12509451;15940638 |
| Id1 | Tnf | Activation | 17012234 |
| Ikbkb | Tnf | Activation | 14691250 |
| Il17a | Tnf | Activation | 16798734 |
| Irf1 | Tnf | Activation | 25957166 |
| Irf1 | Tnf | Unknown | 18802049 |
| Irf8 | Tnf | Unknown | 18802049 |
| Jun | Tnf | Unknown | 10754326;11028563;11739517;12509451 |
| Nfatc2 | Tnf | Unknown | 8668213 |
| Nfe2 | Tnf | Unknown | 9826775 |
| Nfkb1 | Tnf | Activation | 10437651;10754326;11179036;11243852 |
| Nfkb1 | Tnf | Repression | 15465827 |
| Nfkb1 | Tnf | Unknown | 10497896;11312646;11739517;11856733; |
| Nr5a2 | Tnf | Activation | 15684064 |
| Rel | Tnf | Activation | 10437651 |
| Rel | Tnf | Repression | 17012234 |
| Rela | Tnf | Activation | 10754326;17570221;22661093;8622636 |
| Rela | Tnf | Repression | 20097764 |
| Rela | Tnf | Unknown | 11739517;15465827;19285436;26004152 |
| Sp1 | Tnf | Unknown | 12509451;20221720 |
| Spi1 | Tnf | Activation | 19646961;22454293 |
| Stat3 | Tnf | Unknown | 11028563 |
| Stat6 | Tnf | Unknown | 11028563 |
| Twist1 | Tnf | Repression | 16831897 |
| **# [TF]** | **[Target]** | **[Type]** | **[Reference]** |
| Ash1l | Tgfb1 | Unknown | 22488473 |
| Ctf1 | Tgfb1 | Unknown | 8543151 |
| Elf3 | Tgfb1 | Unknown | 10644990 |
| Fos | Tgfb1 | Activation | 16637060 |
| Fosl2 | Tgfb1 | Activation | 20427335 |
| Jun | Tgfb1 | Activation | 17270292 |
| Nfe2l2 | Tgfb1 | Repression | 20103708 |
| Nfkb1 | Tgfb1 | Activation | 19657147 |
| Ppard | Tgfb1 | Activation | 18007025 |
| Smad4 | Tgfb1 | Repression | 15312519 |
| Smad7 | Tgfb1 | Activation | 18762808 |
| Stat3 | Tgfb1 | Activation | 16474852 |

***Supplementary Table S5: KEGG of Inflammatory Genes.***

| **Human KEGG-Inflammatory Genes** | | | | | | | | |
| --- | --- | --- | --- | --- | --- | --- | --- | --- |
| **ID** | **Description** | **GeneRatio** | **BgRatio** | **pvalue** | **p.adjust** | **qvalue** | **geneID** | **Count** |
| hsa04512 | ECM-receptor interaction | 14/58 | 88/8093 | 7.62E-16 | 1.01E-13 | 7.70E-14 | 1277/1278/1282/1293/1311/2335/3673/3685/3688/3693/284217/3908/6696/7058 | 14 |
| hsa04510 | Focal adhesion | 15/58 | 201/8093 | 5.82E-12 | 3.87E-10 | 2.94E-10 | 1277/1278/1282/1293/1311/2335/3479/3673/3685/3688/3693/284217/3908/6696/7058 | 15 |
| hsa04151 | PI3K-Akt signaling pathway | 17/58 | 354/8093 | 2.06E-10 | 9.11E-09 | 6.92E-09 | 1277/1278/1282/1293/1311/356/2252/2335/3479/3673/3685/3688/3693/284217/3908/6696/7058 | 17 |
| hsa05165 | Human papillomavirus infection | 16/58 | 331/8093 | 7.16E-10 | 2.38E-08 | 1.81E-08 | 1277/1278/1282/1293/1311/356/2335/3673/3685/3688/3693/284217/3908/4853/6696/7058 | 16 |
| hsa04974 | Protein digestion and absorption | 10/58 | 103/8093 | 2.25E-09 | 5.98E-08 | 4.55E-08 | 1301/1277/1278/1281/1282/1289/1290/1293/1294/5645 | 10 |
| hsa05410 | Hypertrophic cardiomyopathy | 9/58 | 90/8093 | 1.18E-08 | 2.62E-07 | 1.99E-07 | 1906/3479/3673/3685/3688/3693/284217/3908/6445 | 9 |
| hsa05205 | Proteoglycans in cancer | 12/58 | 205/8093 | 1.62E-08 | 3.08E-07 | 2.34E-07 | 1277/1278/1634/356/2335/3479/3673/3685/3688/3693/4060/4313 | 12 |
| hsa04933 | AGE-RAGE signaling pathway in diabetic complications | 9/58 | 100/8093 | 3.01E-08 | 5.00E-07 | 3.80E-07 | 1277/1278/1281/1282/1906/2335/4313/5054/7412 | 9 |
| hsa05412 | Arrhythmogenic right ventricular cardiomyopathy | 8/58 | 77/8093 | 6.06E-08 | 8.95E-07 | 6.80E-07 | 2697/3673/3685/3688/3693/284217/3908/6445 | 8 |
| hsa05414 | Dilated cardiomyopathy | 8/58 | 96/8093 | 3.44E-07 | 4.57E-06 | 3.47E-06 | 3479/3673/3685/3688/3693/284217/3908/6445 | 8 |
| hsa05222 | Small cell lung cancer | 7/58 | 92/8093 | 3.66E-06 | 4.42E-05 | 3.36E-05 | 1282/2335/3673/3685/3688/284217/3908 | 7 |
| hsa05146 | Amoebiasis | 7/58 | 102/8093 | 7.29E-06 | 8.08E-05 | 6.14E-05 | 1277/1278/1281/1282/2335/284217/3908 | 7 |
| hsa04611 | Platelet activation | 6/58 | 124/8093 | 2.42E-04 | 2.47E-03 | 1.88E-03 | 1277/1278/1281/3673/3688/10125 | 6 |
| hsa04926 | Relaxin signaling pathway | 6/58 | 129/8093 | 3.00E-04 | 2.85E-03 | 2.16E-03 | 1277/1278/1281/1282/1906/4313 | 6 |
| hsa04061 | Viral protein interaction with cytokine and cytokine receptor | 5/58 | 100/8093 | 7.11E-04 | 5.98E-03 | 4.54E-03 | 6356/6348/6351/6352/6387 | 5 |
| hsa04145 | Phagosome | 6/58 | 152/8093 | 7.19E-04 | 5.98E-03 | 4.54E-03 | 1311/3673/3685/3688/3693/7058 | 6 |
| hsa04810 | Regulation of actin cytoskeleton | 7/58 | 218/8093 | 8.74E-04 | 0.006838623 | 0.005195946 | 6387/2252/2335/3673/3685/3688/3693 | 7 |
| hsa04670 | Leukocyte transendothelial migration | 5/58 | 114/8093 | 1.28E-03 | 0.009488115 | 0.007209015 | 6387/3688/4313/7070/7412 | 5 |
| hsa04514 | Cell adhesion molecules | 5/58 | 149/8093 | 4.14E-03 | 0.028715973 | 0.021818231 | 1493/3685/3688/7412/1462 | 5 |
| hsa05323 | Rheumatoid arthritis | 4/58 | 93/8093 | 4.32E-03 | 0.028715973 | 0.021818231 | 6348/6352/1493/6387 | 4 |
| hsa04060 | Cytokine-cytokine receptor interaction | 7/58 | 295/8093 | 4.86E-03 | 0.030799893 | 0.023401581 | 6356/6348/6351/6352/6387/356/4982 | 7 |
| hsa05163 | Human cytomegalovirus infection | 6/58 | 225/8093 | 5.23E-03 | 0.031186258 | 0.023695138 | 6348/6351/6352/6387/356/3685 | 6 |
| hsa05144 | Malaria | 3/58 | 50/8093 | 0.005393112 | 0.031186258 | 0.023695138 | 1311/7058/7412 | 3 |
| hsa05142 | Chagas disease | 4/58 | 102/8093 | 0.005992974 | 0.033211066 | 0.025233576 | 6348/6352/356/5054 | 4 |
| hsa04620 | Toll-like receptor signaling pathway | 4/58 | 104/8093 | 0.006416384 | 0.034135162 | 0.025935699 | 6348/6351/6352/6696 | 4 |
| hsa00511 | Other glycan degradation | 2/58 | 18/8093 | 0.007174813 | 0.036701926 | 0.027885912 | 2517/2629 | 2 |
| hsa04066 | HIF-1 signaling pathway | 4/58 | 109/8093 | 0.007560943 | 0.037244647 | 0.028298267 | 1906/3479/5054/7076 | 4 |
| hsa04668 | TNF signaling pathway | 4/58 | 112/8093 | 0.008308516 | 0.039465451 | 0.029985621 | 6352/1906/4323/7412 | 4 |
| **Mouse KEGG-Inflammatory Genes** | | | | | | | | |
| **ID** | **Description** | **GeneRatio** | **BgRatio** | **pvalue** | **p.adjust** | **qvalue** | **geneID** | **Count** |
| hsa04512 | ECM-receptor interaction | 23/131 | 88/8143 | 2.80E-22 | 5.55E-20 | 3.98E-20 | 960/1277/1278/1282/1284/1292/1293/1311/2335/3678/3685/3688/3690/3693/3909/3915/3918/6382/6385/6696/7057/7058/3371 | 23 |
| hsa04510 | Focal adhesion | 29/131 | 201/8143 | 3.60E-20 | 3.57E-18 | 2.56E-18 | 1277/1278/1282/1284/1292/1293/1311/1950/2316/2335/3479/3678/3685/3688/3690/3693/3725/3909/3915/3918/10398/5154/5155/5159/6696/7057/7058/3371/7424 | 29 |
| hsa04151 | PI3K-Akt signaling pathway | 32/131 | 354/8143 | 4.63E-16 | 3.05E-14 | 2.19E-14 | 374/1277/1278/1282/1284/1292/1293/1311/1440/1950/2247/2252/2335/3479/3569/3678/3685/3688/3690/3693/3909/3915/3918/5154/5155/5159/6696/7039/7057/7058/3371/7424 | 32 |
| hsa05205 | Proteoglycans in cancer | 21/131 | 205/8143 | 8.74E-12 | 4.33E-10 | 3.10E-10 | 960/1277/1278/2247/2316/2335/8325/3479/3678/3685/3688/3690/3693/4313/5328/5329/6382/6385/7040/7057/7078 | 21 |
| hsa04933 | AGE-RAGE signaling pathway | 15/131 | 100/8143 | 4.43E-11 | 1.75E-09 | 1.26E-09 | 1277/1278/1281/1282/1284/1906/2335/3553/3569/3725/4313/5580/7040/7412/7424 | 15 |
| hsa05165 | Human papillomavirus infection | 25/131 | 331/8143 | 6.48E-11 | 1.94E-09 | 1.39E-09 | 1277/1278/1282/1284/1292/1293/1311/1950/2335/8325/3678/3685/3688/3690/3693/3909/3915/3918/4853/5159/6696/7015/7057/7058/3371 | 25 |
| hsa04974 | Protein digestion and absorption | 15/131 | 103/8143 | 6.86E-11 | 1.94E-09 | 1.39E-09 | 1301/1277/1278/1281/1282/1284/1289/1290/50509/1292/1293/1294/1296/2006/5645 | 15 |
| hsa05410 | Hypertrophic cardiomyopathy | 13/131 | 90/8143 | 1.52E-09 | 3.77E-08 | 2.70E-08 | 1906/3479/3569/3678/3685/3688/3690/3693/6443/6445/7040/7169/7171 | 13 |
| hsa05144 | Malaria | 10/131 | 50/8143 | 4.89E-09 | 1.08E-07 | 7.72E-08 | 1311/1440/3553/3569/4035/6382/7040/7057/7058/7412 | 10 |
| hsa05146 | Amoebiasis | 13/131 | 102/8143 | 7.36E-09 | 1.46E-07 | 1.05E-07 | 1277/1278/1281/1282/1284/2920/2335/3553/3569/3909/3915/3918/7040 | 13 |
| hsa04657 | IL-17 signaling pathway | 11/131 | 94/8143 | 2.72E-07 | 4.90E-06 | 3.52E-06 | 1051/1440/2920/3596/3553/3567/3569/3725/4314/727897/7128 | 11 |
| hsa05414 | Dilated cardiomyopathy | 11/131 | 96/8143 | 3.38E-07 | 5.58E-06 | 4.01E-06 | 3479/3678/3685/3688/3690/3693/6443/6445/7040/7169/7171 | 11 |
| hsa04668 | TNF signaling pathway | 11/131 | 112/8143 | 1.62E-06 | 2.47E-05 | 1.77E-05 | 1051/2920/1906/3553/3569/3725/4323/4314/7128/7412/7424 | 11 |
| hsa04810 | Regulation of actin cytoskeleton | 15/131 | 218/8143 | 1.96E-06 | 2.78E-05 | 1.99E-05 | 6387/1950/2247/2252/2335/3678/3685/3688/3690/3693/10398/5154/5155/5159/5217 | 15 |
| hsa05418 | Fluid shear stress and atherosclerosis | 12/131 | 139/8143 | 2.13E-06 | 2.81E-05 | 2.02E-05 | 1906/3162/3553/3685/3690/3725/4313/5154/5155/6382/6385/7412 | 12 |
| hsa05412 | Arrhythmogenicrightventricular cardiomyopathy | 9/131 | 77/8143 | 3.51E-06 | 4.34E-05 | 3.12E-05 | 1000/2697/3678/3685/3688/3690/3693/6443/6445 | 9 |
| hsa05222 | Small cell lung cancer | 9/131 | 92/8143 | 1.54E-05 | 0.00018 | 0.000129 | 1282/1284/2335/4616/3685/3688/3909/3915/3918 | 9 |
| hsa04010 | MAPK signaling pathway | 16/131 | 294/8143 | 1.80E-05 | 0.00019 | 0.000137 | 374/1950/2247/2252/2316/4616/3479/3553/3725/5154/5155/5159/10125/7039/7040/7424 | 16 |
| hsa05218 | Melanoma | 8/131 | 72/8143 | 1.83E-05 | 0.00019 | 0.000137 | 1950/2247/2252/4616/3479/5154/5155/5159 | 8 |
| hsa01521 | EGFR tyrosine kinase inhibitor resistance | 8/131 | 79/8143 | 3.62E-05 | 0.000359 | 0.000257 | 1950/2247/3479/3569/5154/5155/5159/7039 | 8 |
| hsa04926 | Relaxin signaling pathway | 10/131 | 129/8143 | 4.00E-05 | 0.000377 | 0.000271 | 1277/1278/1281/1282/1284/1906/3725/4313/7040/7424 | 10 |
| hsa04060 | Cytokine-cytokine receptor interaction | 15/131 | 295/8143 | 7.32E-05 | 0.000659 | 0.000473 | 9210/655/729230/1232/1440/6387/2920/3596/3553/3557/3567/3569/7040/4982/51330 | 15 |
| hsa04015 | Rap1 signaling pathway | 12/131 | 210/8143 | 0.000135 | 0.001164 | 0.000836 | 1950/2247/2252/3479/3688/3690/5154/5155/5159/5217/7057/7424 | 12 |
| hsa05215 | Prostate cancer | 8/131 | 97/8143 | 0.000158 | 0.0013 | 0.000933 | 1950/3479/4314/5154/5155/5159/5328/7039 | 8 |
| hsa04640 | Hematopoietic cell lineage | 8/131 | 99/8143 | 0.000182 | 0.001439 | 0.001033 | 290/960/1440/3553/3567/3569/3678/3690 | 8 |
| hsa05214 | Glioma | 7/131 | 75/8143 | 0.00019 | 0.001446 | 0.001038 | 1950/4616/3479/5154/5155/5159/7039 | 7 |
| hsa05321 | Inflammatory bowel disease | 6/131 | 65/8143 | 0.000587 | 0.004303 | 0.003088 | 3596/3553/3567/3569/3725/7040 | 6 |
| hsa05323 | Rheumatoid arthritis | 7/131 | 93/8143 | 0.000714 | 0.005046 | 0.003622 | 6387/2920/3553/3569/3725/4314/7040 | 7 |

***Supplementary Table S6: PRDX4 gene expression and prognostic data in BALF from 176 IPF patients.***

| **accession** | **title** | **source** | **diagnosis** | **age** | **sex (0=female, 1=male)** | **survival status, 0 = censored, 1 = death** | **gap** | **time to death (days)** | **PRDX4 gene expression** |
| --- | --- | --- | --- | --- | --- | --- | --- | --- | --- |
| GSM1820739 | BAL_IPF_Freiburg_1 | BAL cells | IPF | 65 | 1 | 0 | 3 | 2926 | 7.604669 |
| GSM1820740 | BAL_IPF_Freiburg_2 | BAL cells | IPF | 76 | 1 | 1 | 5 | 305 | 7.53582 |
| GSM1820741 | BAL_IPF_Freiburg_3 | BAL cells | IPF | 68 | 1 | 1 | 6 | 557 | 7.772195 |
| GSM1820742 | BAL_IPF_Freiburg_4 | BAL cells | IPF | 56 | 1 | 1 | 5 | 151 | 7.522856 |
| GSM1820743 | BAL_IPF_Freiburg_5 | BAL cells | IPF | 70 | 1 | 1 | 3 | 1404 | 7.603686 |
| GSM1820744 | BAL_IPF_Freiburg_6 | BAL cells | IPF | 77 | 1 | 1 | 5 | 718 | 7.655991 |
| GSM1820745 | BAL_IPF_Freiburg_7 | BAL cells | IPF | 54 | 1 | 1 | 3 | 167 | 7.370375 |
| GSM1820746 | BAL_IPF_Freiburg_8 | BAL cells | IPF | 69 | 1 | 1 | 4 | 1054 | 7.557413 |
| GSM1820747 | BAL_IPF_Freiburg_9 | BAL cells | IPF | 49 | 1 | 1 | 2 | 574 | 7.569262 |
| GSM1820748 | BAL_IPF_Freiburg_10 | BAL cells | IPF | 79 | 0 | 1 | 2 | 198 | 7.457667 |
| GSM1820749 | BAL_IPF_Freiburg_11 | BAL cells | IPF | 78 | 0 | 1 | 5 | 1081 | 7.734712 |
| GSM1820750 | BAL_IPF_Freiburg_12 | BAL cells | IPF | 58 | 1 | 1 | 1 | 982 | 7.680876 |
| GSM1820751 | BAL_IPF_Freiburg_13 | BAL cells | IPF | 74 | 0 | 1 | 4 | 1272 | 7.528812 |
| GSM1820752 | BAL_IPF_Freiburg_14 | BAL cells | IPF | 76 | 1 | 0 | 5 | 2152 | 7.405096 |
| GSM1820753 | BAL_IPF_Freiburg_15 | BAL cells | IPF | 75 | 1 | 1 | 5 | 811 | 7.440925 |
| GSM1820754 | BAL_IPF_Freiburg_16 | BAL cells | IPF | 74 | 1 | 0 | 4 | 2498 | 7.452227 |
| GSM1820755 | BAL_IPF_Freiburg_17 | BAL cells | IPF | 76 | 1 | 1 | 8 | 97 | 7.58361 |
| GSM1820756 | BAL_IPF_Freiburg_18 | BAL cells | IPF | 72 | 1 | 1 | 5 | 82 | 7.406019 |
| GSM1820757 | BAL_IPF_Freiburg_19 | BAL cells | IPF | 76 | 1 | 1 | 7 | 824 | 7.591182 |
| GSM1820758 | BAL_IPF_Freiburg_20 | BAL cells | IPF | 72 | 0 | 1 | 4 | 620 | 7.415174 |
| GSM1820759 | BAL_IPF_Freiburg_21 | BAL cells | IPF | 56 | 1 | 0 | 2 | 1965 | 7.792031 |
| GSM1820760 | BAL_IPF_Freiburg_22 | BAL cells | IPF | 63 | 1 | 0 | 5 | 2237 | 7.460738 |
| GSM1820761 | BAL_IPF_Freiburg_23 | BAL cells | IPF | 50 | 1 | 1 | 4 | 373 | 7.440925 |
| GSM1820762 | BAL_IPF_Freiburg_24 | BAL cells | IPF | 67 | 1 | 1 | 7 | 933 | 7.786235 |
| GSM1820763 | BAL_IPF_Freiburg_25 | BAL cells | IPF | 77 | 0 | 0 | 2 | 1993 | 7.569262 |
| GSM1820764 | BAL_IPF_Freiburg_26 | BAL cells | IPF | 65 | 1 | 1 | 6 | 1176 | 7.661064 |
| GSM1820765 | BAL_IPF_Freiburg_27 | BAL cells | IPF | 78 | 1 | 1 | 5 | 221 | 7.793481 |
| GSM1820766 | BAL_IPF_Freiburg_28 | BAL cells | IPF | 63 | 1 | 1 | 5 | 975 | 7.650231 |
| GSM1820767 | BAL_IPF_Freiburg_29 | BAL cells | IPF | 53 | 1 | 1 | 2 | 1475 | 7.76406 |
| GSM1820768 | BAL_IPF_Freiburg_30 | BAL cells | IPF | 73 | 1 | 1 | 3 | 1769 | 7.650231 |
| GSM1820769 | BAL_IPF_Freiburg_31 | BAL cells | IPF | 74 | 1 | 1 | 6 | 277 | 7.438279 |
| GSM1820770 | BAL_IPF_Freiburg_32 | BAL cells | IPF | 75 | 1 | 1 | 4 | 1140 | 7.841065 |
| GSM1820771 | BAL_IPF_Freiburg_33 | BAL cells | IPF | 72 | 1 | 0 | 4 | 1813 | 7.489059 |
| GSM1820772 | BAL_IPF_Freiburg_34 | BAL cells | IPF | 70 | 1 | 1 | 4 | 432 | 7.818379 |
| GSM1820773 | BAL_IPF_Freiburg_35 | BAL cells | IPF | 77 | 1 | 1 | 8 | 905 | 7.45318 |
| GSM1820774 | BAL_IPF_Freiburg_36 | BAL cells | IPF | 54 | 1 | 1 | 2 | 893 | 7.563497 |
| GSM1820775 | BAL_IPF_Freiburg_37 | BAL cells | IPF | 68 | 1 | 0 | 3 | 1580 | 7.623622 |
| GSM1820776 | BAL_IPF_Freiburg_38 | BAL cells | IPF | 74 | 1 | 0 | 5 | 1461 | 7.658813 |
| GSM1820777 | BAL_IPF_Freiburg_39 | BAL cells | IPF | 60 | 1 | 1 | 3 | 1055 | 7.592305 |
| GSM1820778 | BAL_IPF_Freiburg_40 | BAL cells | IPF | 61 | 0 | 1 | 5 | 45 | 7.592305 |
| GSM1820779 | BAL_IPF_Freiburg_41 | BAL cells | IPF | 76 | 1 | 1 | 5 | 547 | 6.68127 |
| GSM1820780 | BAL_IPF_Freiburg_42 | BAL cells | IPF | 68 | 1 | 0 | 3 | 971 | 7.712513 |
| GSM1820781 | BAL_IPF_Freiburg_43 | BAL cells | IPF | 67 | 1 | 1 | 6 | 599 | 7.687603 |
| GSM1820782 | BAL_IPF_Freiburg_44 | BAL cells | IPF | 77 | 0 | 1 | 4 | 535 | 7.432305 |
| GSM1820783 | BAL_IPF_Freiburg_45 | BAL cells | IPF | 66 | 1 | 1 | 7 | 59 | 7.783429 |
| GSM1820784 | BAL_IPF_Freiburg_46 | BAL cells | IPF | 59 | 1 | 1 | 5 | 210 | 7.570295 |
| GSM1820785 | BAL_IPF_Freiburg_47 | BAL cells | IPF | 54 | 1 | 0 | 4 | 698 | 7.640677 |
| GSM1820786 | BAL_IPF_Freiburg_48 | BAL cells | IPF | 63 | 1 | 1 | 5 | 226 | 7.671432 |
| GSM1820787 | BAL_IPF_Freiburg_49 | BAL cells | IPF | 67 | 1 | 0 | 4 | 670 | 7.684313 |
| GSM1820788 | BAL_IPF_Freiburg_50 | BAL cells | IPF | 71 | 0 | 1 | 4 | 98 | 7.513424 |
| GSM1820789 | BAL_IPF_Freiburg_51 | BAL cells | IPF | 84 | 1 | 0 | 4 | 636 | 7.525598 |
| GSM1820790 | BAL_IPF_Freiburg_52 | BAL cells | IPF | 69 | 1 | 0 | 3 | 613 | 7.666816 |
| GSM1820791 | BAL_IPF_Freiburg_53 | BAL cells | IPF | 71 | 1 | 0 | 3 | 594 | 7.841065 |
| GSM1820792 | BAL_IPF_Freiburg_54 | BAL cells | IPF | 48 | 1 | 1 | 5 | 306 | 7.745305 |
| GSM1820793 | BAL_IPF_Freiburg_55 | BAL cells | IPF | 67 | 1 | 1 | 7 | 80 | 7.348471 |
| GSM1820794 | BAL_IPF_Freiburg_56 | BAL cells | IPF | 71 | 1 | 1 | 5 | 565 | 7.767017 |
| GSM1820795 | BAL_IPF_Freiburg_57 | BAL cells | IPF | 78 | 0 | 1 | 2 | 150 | 7.717771 |
| GSM1820796 | BAL_IPF_Freiburg_58 | BAL cells | IPF | 69 | 1 | 0 | 8 | 523 | 7.727432 |
| GSM1820797 | BAL_IPF_Freiburg_59 | BAL cells | IPF | 65 | 1 | 0 | 7 | 839 | 7.657373 |
| GSM1820798 | BAL_IPF_Freiburg_60 | BAL cells | IPF | 44 | 1 | 1 | 6 | 66 | 7.752862 |
| GSM1820799 | BAL_IPF_Freiburg_61 | BAL cells | IPF | 68 | 1 | 1 | 5 | 489 | 7.559869 |
| GSM1820800 | BAL_IPF_Freiburg_62 | BAL cells | IPF | 53 | 1 | 1 | 4 | 21 | 7.688878 |
| GSM1820801 | BAL_IPF_Siena_1 | BAL cells | IPF | 51 | 1 | 1 | 2 | 89 | 7.670843 |
| GSM1820802 | BAL_IPF_Siena_2 | BAL cells | IPF | 60 | 1 | 0 | 1 | 551 | 7.801639 |
| GSM1820803 | BAL_IPF_Siena_3 | BAL cells | IPF | 49 | 1 | 1 | 2 | 128 | 7.590009 |
| GSM1820804 | BAL_IPF_Siena_4 | BAL cells | IPF | 83 | 1 | 0 | 8 | 468 | 7.715024 |
| GSM1820805 | BAL_IPF_Siena_5 | BAL cells | IPF | 54 | 0 | 1 | 1 | 840 | 7.742008 |
| GSM1820806 | BAL_IPF_Siena_6 | BAL cells | IPF | 82 | 1 | 1 | 7 | 197 | 7.832431 |
| GSM1820807 | BAL_IPF_Siena_7 | BAL cells | IPF | 82 | 0 | 1 | 7 | 472 | 7.773987 |
| GSM1820808 | BAL_IPF_Siena_8 | BAL cells | IPF | 68 | 0 | 0 | 6 | 872 | 7.471689 |
| GSM1820809 | BAL_IPF_Siena_9 | BAL cells | IPF | 81 | 0 | 1 | 3 | 787 | 7.840083 |
| GSM1820810 | BAL_IPF_Siena_10 | BAL cells | IPF | 75 | 1 | 0 | 4 | 1054 | 7.697676 |
| GSM1820811 | BAL_IPF_Siena_11 | BAL cells | IPF | 74 | 1 | 0 | 4 | 1052 | 7.707141 |
| GSM1820812 | BAL_IPF_Siena_12 | BAL cells | IPF | 62 | 1 | 1 | 7 | 378 | 7.704807 |
| GSM1820813 | BAL_IPF_Siena_13 | BAL cells | IPF | 76 | 1 | 1 | 5 | 688 | 7.866945 |
| GSM1820814 | BAL_IPF_Siena_14 | BAL cells | IPF | 66 | 1 | 1 | 6 | 493 | 7.614641 |
| GSM1820815 | BAL_IPF_Siena_15 | BAL cells | IPF | 86 | 1 | 1 | 6 | 958 | 7.853094 |
| GSM1820816 | BAL_IPF_Siena_16 | BAL cells | IPF | 70 | 1 | 0 | 4 | 863 | 7.688484 |
| GSM1820817 | BAL_IPF_Siena_17 | BAL cells | IPF | 62 | 1 | 1 | 5 | 586 | 7.853645 |
| GSM1820818 | BAL_IPF_Siena_18 | BAL cells | IPF | 81 | 0 | 0 | 3 | 1105 | 7.672526 |
| GSM1820819 | BAL_IPF_Siena_19 | BAL cells | IPF | 70 | 1 | 1 | 4 | 1171 | 7.812099 |
| GSM1820820 | BAL_IPF_Siena_20 | BAL cells | IPF | 78 | 1 | 1 | 7 | 110 | 7.808062 |
| GSM1820821 | BAL_IPF_Siena_21 | BAL cells | IPF | 64 | 1 | 1 | 5 | 472 | 7.700436 |
| GSM1820822 | BAL_IPF_Siena_22 | BAL cells | IPF | 79 | 1 | 1 | 6 | 139 | 7.858225 |
| GSM1820823 | BAL_IPF_Siena_23 | BAL cells | IPF | 66 | 0 | 0 | 4 | 841 | 7.645597 |
| GSM1820824 | BAL_IPF_Siena_24 | BAL cells | IPF | 62 | 1 | 1 | 5 | 294 | 7.771609 |
| GSM1820825 | BAL_IPF_Siena_25 | BAL cells | IPF | 61 | 1 | 1 | 7 | 388 | 7.742021 |
| GSM1820826 | BAL_IPF_Siena_26 | BAL cells | IPF | 73 | 1 | 1 | 8 | 318 | 7.639473 |
| GSM1820827 | BAL_IPF_Siena_27 | BAL cells | IPF | 81 | 1 | 0 | 4 | 1104 | 7.612103 |
| GSM1820828 | BAL_IPF_Siena_28 | BAL cells | IPF | 68 | 0 | 0 | 2 | 1097 | 7.727002 |
| GSM1820829 | BAL_IPF_Siena_29 | BAL cells | IPF | 72 | 1 | 1 | 8 | 42 | 7.729413 |
| GSM1820830 | BAL_IPF_Siena_30 | BAL cells | IPF | 84 | 1 | 1 | 4 | 276 | 7.714852 |
| GSM1820831 | BAL_IPF_Siena_31 | BAL cells | IPF | 62 | 1 | 1 | 7 | 79 | 7.720212 |
| GSM1820832 | BAL_IPF_Siena_32 | BAL cells | IPF | 72 | 1 | 0 | 5 | 1027 | 7.646028 |
| GSM1820833 | BAL_IPF_Siena_33 | BAL cells | IPF | 55 | 1 | 1 | 4 | 557 | 7.742366 |
| GSM1820834 | BAL_IPF_Siena_34 | BAL cells | IPF | 40 | 1 | 0 | 2 | 660 | 7.701095 |
| GSM1820835 | BAL_IPF_Siena_35 | BAL cells | IPF | 72 | 1 | 1 | 7 | 144 | 7.754805 |
| GSM1820836 | BAL_IPF_Siena_36 | BAL cells | IPF | 73 | 1 | 1 | 4 | 271 | 7.753046 |
| GSM1820837 | BAL_IPF_Siena_37 | BAL cells | IPF | 62 | 1 | 0 | 4 | 619 | 7.523708 |
| GSM1820838 | BAL_IPF_Siena_38 | BAL cells | IPF | 79 | 0 | 0 | 3 | 859 | 7.566756 |
| GSM1820839 | BAL_IPF_Siena_39 | BAL cells | IPF | 88 | 0 | 0 | 3 | 583 | 7.73304 |
| GSM1820840 | BAL_IPF_Siena_40 | BAL cells | IPF | 62 | 0 | 1 | 6 | 731 | 7.483655 |
| GSM1820841 | BAL_IPF_Siena_41 | BAL cells | IPF | 41 | 1 | 0 | 3 | 549 | 7.626126 |
| GSM1820842 | BAL_IPF_Siena_42 | BAL cells | IPF | 59 | 1 | 0 | 5 | 487 | 7.754883 |
| GSM1820843 | BAL_IPF_Siena_43 | BAL cells | IPF | 75 | 1 | 1 | 4 | 540 | 7.685289 |
| GSM1820844 | BAL_IPF_Siena_44 | BAL cells | IPF | 79 | 1 | 1 | 3 | 282 | 7.883571 |
| GSM1820845 | BAL_IPF_Siena_45 | BAL cells | IPF | 60 | 1 | 0 | 3 | 459 | 7.804024 |
| GSM1820846 | BAL_IPF_Siena_46 | BAL cells | IPF | 71 | 1 | 1 | 3 | 426 | 7.614182 |
| GSM1820847 | BAL_IPF_Siena_47 | BAL cells | IPF | 76 | 1 | 1 | 5 | 401 | 7.46143 |
| GSM1820848 | BAL_IPF_Siena_48 | BAL cells | IPF | 71 | 1 | 0 | 4 | 383 | 7.433118 |
| GSM1820849 | BAL_IPF_Siena_49 | BAL cells | IPF | 62 | 1 | 1 | 7 | 369 | 7.713767 |
| GSM1820850 | BAL_IPF_Siena_50 | BAL cells | IPF | 55 | 1 | 1 | 4 | 244 | 7.770446 |
| GSM1820851 | BAL_IPF_Leuven_1 | BAL cells | IPF | 74 | 0 | 1 | 3 | 1443 | 14.22534 |
| GSM1820852 | BAL_IPF_Leuven_2 | BAL cells | IPF | 64 | 1 | 0 | 2 | 587 | 14.4344 |
| GSM1820853 | BAL_IPF_Leuven_3 | BAL cells | IPF | 64 | 1 | 1 | 5 | 214 | 14.42985 |
| GSM1820854 | BAL_IPF_Leuven_4 | BAL cells | IPF | 58 | 1 | 0 | 4 | 362 | 13.87081 |
| GSM1820855 | BAL_IPF_Leuven_5 | BAL cells | IPF | 67 | 1 | 1 | 6 | 468 | 14.58098 |
| GSM1820856 | BAL_IPF_Leuven_6 | BAL cells | IPF | 63 | 1 | 1 | 4 | 676 | 14.53943 |
| GSM1820857 | BAL_IPF_Leuven_7 | BAL cells | IPF | 60 | 1 | 0 | 3 | 1422 | 14.57159 |
| GSM1820858 | BAL_IPF_Leuven_8 | BAL cells | IPF | 64 | 1 | 1 | 4 | 1472 | 13.47606 |
| GSM1820859 | BAL_IPF_Leuven_9 | BAL cells | IPF | 71 | 1 | 0 | 4 | 433 | 14.25022 |
| GSM1820860 | BAL_IPF_Leuven_10 | BAL cells | IPF | 76 | 0 | 0 | 3 | 1271 | 13.92509 |
| GSM1820861 | BAL_IPF_Leuven_11 | BAL cells | IPF | 66 | 1 | 1 | 4 | 1281 | 14.05337 |
| GSM1820862 | BAL_IPF_Leuven_12 | BAL cells | IPF | 65 | 1 | 0 | 3 | 699 | 14.6118 |
| GSM1820863 | BAL_IPF_Leuven_13 | BAL cells | IPF | 68 | 1 | 1 | 6 | 351 | 13.91493 |
| GSM1820864 | BAL_IPF_Leuven_14 | BAL cells | IPF | 77 | 1 | 0 | 4 | 328 | 14.2007 |
| GSM1820865 | BAL_IPF_Leuven_15 | BAL cells | IPF | 53 | 1 | 0 | 2 | 529 | 13.73397 |
| GSM1820866 | BAL_IPF_Leuven_16 | BAL cells | IPF | 64 | 1 | 0 | 4 | 846 | 14.24518 |
| GSM1820867 | BAL_IPF_Leuven_17 | BAL cells | IPF | 75 | 1 | 0 | 4 | 699 | 14.58414 |
| GSM1820868 | BAL_IPF_Leuven_18 | BAL cells | IPF | 62 | 0 | 0 | 2 | 419 | 14.86202 |
| GSM1820869 | BAL_IPF_Leuven_19 | BAL cells | IPF | 52 | 1 | 0 | 2 | 1253 | 14.23956 |
| GSM1820870 | BAL_IPF_Leuven_20 | BAL cells | IPF | 72 | 1 | 0 | 3 | 697 | 14.25749 |
| GSM1820871 | BAL_IPF_Leuven_21 | BAL cells | IPF | 72 | 0 | 0 | 2 | 1240 | 14.24518 |
| GSM1820872 | BAL_IPF_Leuven_22 | BAL cells | IPF | 71 | 1 | 1 | 7 | 1394 | 14.46482 |
| GSM1820873 | BAL_IPF_Leuven_23 | BAL cells | IPF | 51 | 1 | 0 | 4 | 1439 | 14.22038 |
| GSM1820874 | BAL_IPF_Leuven_24 | BAL cells | IPF | 60 | 1 | 1 | 4 | 637 | 14.17126 |
| GSM1820875 | BAL_IPF_Leuven_25 | BAL cells | IPF | 66 | 1 | 0 | 4 | 918 | 14.00017 |
| GSM1820876 | BAL_IPF_Leuven_26 | BAL cells | IPF | 74 | 1 | 0 | 6 | 1817 | 14.76126 |
| GSM1820877 | BAL_IPF_Leuven_27 | BAL cells | IPF | 64 | 1 | 1 | 4 | 299 | 14.92256 |
| GSM1820878 | BAL_IPF_Leuven_28 | BAL cells | IPF | 69 | 1 | 0 | 4 | 377 | 15.00914 |
| GSM1820879 | BAL_IPF_Leuven_29 | BAL cells | IPF | 80 | 0 | 0 | 3 | 546 | 13.73607 |
| GSM1820880 | BAL_IPF_Leuven_30 | BAL cells | IPF | 74 | 1 | 0 | 4 | 758 | 14.35158 |
| GSM1820881 | BAL_IPF_Leuven_31 | BAL cells | IPF | 73 | 1 | 1 | 4 | 541 | 14.36853 |
| GSM1820882 | BAL_IPF_Leuven_32 | BAL cells | IPF | 75 | 1 | 1 | 4 | 93 | 14.32499 |
| GSM1820883 | BAL_IPF_Leuven_33 | BAL cells | IPF | 69 | 0 | 0 | 3 | 902 | 14.23693 |
| GSM1820884 | BAL_IPF_Leuven_34 | BAL cells | IPF | 78 | 1 | 1 | 5 | 395 | 14.08899 |
| GSM1820885 | BAL_IPF_Leuven_35 | BAL cells | IPF | 87 | 1 | 0 | 6 | 665 | 15.00914 |
| GSM1820886 | BAL_IPF_Leuven_36 | BAL cells | IPF | 61 | 0 | 0 | 3 | 1246 | 14.56051 |
| GSM1820887 | BAL_IPF_Leuven_37 | BAL cells | IPF | 79 | 0 | 1 | 4 | 220 | 14.65466 |
| GSM1820888 | BAL_IPF_Leuven_38 | BAL cells | IPF | 71 | 1 | 1 | 4 | 214 | 14.24735 |
| GSM1820889 | BAL_IPF_Leuven_39 | BAL cells | IPF | 64 | 1 | 0 | 2 | 348 | 14.40816 |
| GSM1820890 | BAL_IPF_Leuven_40 | BAL cells | IPF | 52 | 1 | 1 | 3 | 200 | 14.51877 |
| GSM1820891 | BAL_IPF_Leuven_41 | BAL cells | IPF | 60 | 1 | 1 | 4 | 253 | 13.96907 |
| GSM1820892 | BAL_IPF_Leuven_42 | BAL cells | IPF | 62 | 1 | 0 | 2 | 825 | 14.25749 |
| GSM1820893 | BAL_IPF_Leuven_43 | BAL cells | IPF | 77 | 0 | 0 | 3 | 389 | 14.40071 |
| GSM1820894 | BAL_IPF_Leuven_44 | BAL cells | IPF | 73 | 1 | 0 | 3 | 370 | 14.45511 |
| GSM1820895 | BAL_IPF_Leuven_45 | BAL cells | IPF | 65 | 0 | 0 | 1 | 559 | 13.85356 |
| GSM1820896 | BAL_IPF_Leuven_46 | BAL cells | IPF | 76 | 1 | 0 | 4 | 574 | 14.14323 |
| GSM1820897 | BAL_IPF_Leuven_47 | BAL cells | IPF | 51 | 1 | 0 | 2 | 1405 | 13.81789 |
| GSM1820898 | BAL_IPF_Leuven_48 | BAL cells | IPF | 78 | 1 | 1 | 5 | 297 | 14.21178 |
| GSM1820899 | BAL_IPF_Leuven_49 | BAL cells | IPF | 53 | 0 | 0 | 0 | 243 | 14.3037 |
| GSM1820900 | BAL_IPF_Leuven_50 | BAL cells | IPF | 80 | 1 | 0 | 5 | 238 | 13.55422 |
| GSM1820901 | BAL_IPF_Leuven_51 | BAL cells | IPF | 69 | 1 | 0 | 5 | 237 | 14.77169 |
| GSM1820902 | BAL_IPF_Leuven_52 | BAL cells | IPF | 77 | 1 | 1 | 6 | 306 | 14.69075 |
| GSM1820903 | BAL_IPF_Leuven_53 | BAL cells | IPF | 69 | 1 | 1 | 6 | 1038 | 14.4484 |
| GSM1820904 | BAL_IPF_Leuven_54 | BAL cells | IPF | 69 | 0 | 0 | 2 | 895 | 14.11993 |
| GSM1820905 | BAL_IPF_Leuven_55 | BAL cells | IPF | 63 | 1 | 0 | 3 | 1793 | 14.46825 |
| GSM1820906 | BAL_IPF_Leuven_56 | BAL cells | IPF | 65 | 1 | 0 | 3 | 384 | 13.75214 |
| GSM1820907 | BAL_IPF_Leuven_57 | BAL cells | IPF | 68 | 1 | 0 | 5 | 420 | 15.59688 |
| GSM1820908 | BAL_IPF_Leuven_58 | BAL cells | IPF | 53 | 1 | 0 | 2 | 1491 | 14.74312 |
| GSM1820909 | BAL_IPF_Leuven_59 | BAL cells | IPF | 82 | 1 | 1 | 5 | 236 | 16.20714 |
| GSM1820910 | BAL_IPF_Leuven_60 | BAL cells | IPF | 79 | 1 | 0 | 4 | 1098 | 15.50187 |
| GSM1820911 | BAL_IPF_Leuven_61 | BAL cells | IPF | 67 | 0 | 1 | 5 | 340 | 15.03785 |
| GSM1820912 | BAL_IPF_Leuven_62 | BAL cells | IPF | 79 | 1 | 1 | 7 | 728 | 15.22291 |
| GSM1820913 | BAL_IPF_Leuven_63 | BAL cells | IPF | 80 | 1 | 1 | 5 | 133 | 15.6123 |
| GSM1820914 | BAL_IPF_Leuven_64 | BAL cells | IPF | 68 | 1 | 0 | 5 | 939 | 15.01019 |
