# Supplementary figures and images for: Peroxiredoxin 4 as a switch regulating PTEN/AKT axis in alveolar macrophages activation
Source: Signal Transduct Target Ther. 2025 Oct 24;10:352. doi: 10.1038/s41392-025-02454-x (PMC12550102; doi:10.1038/s41392-025-02454-x)

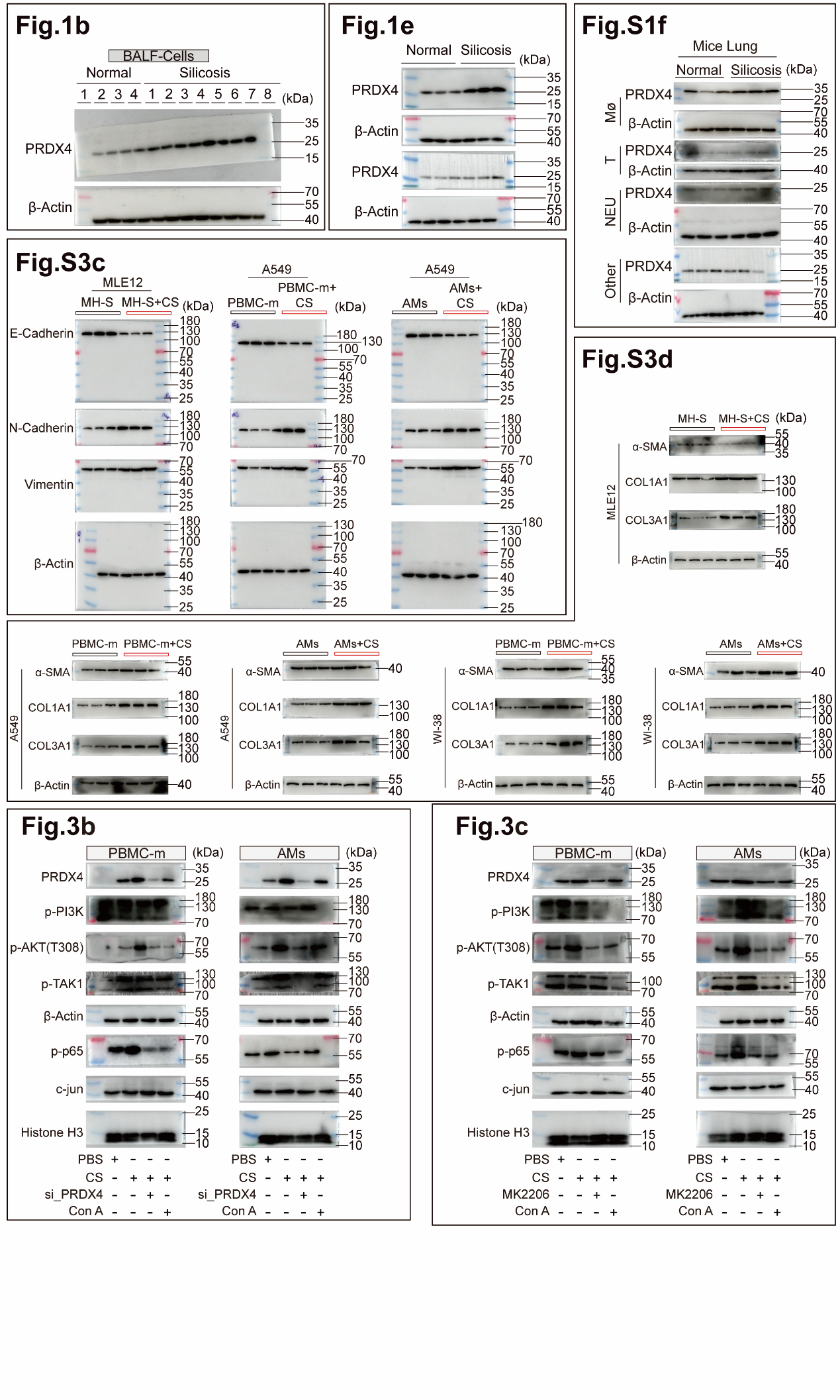


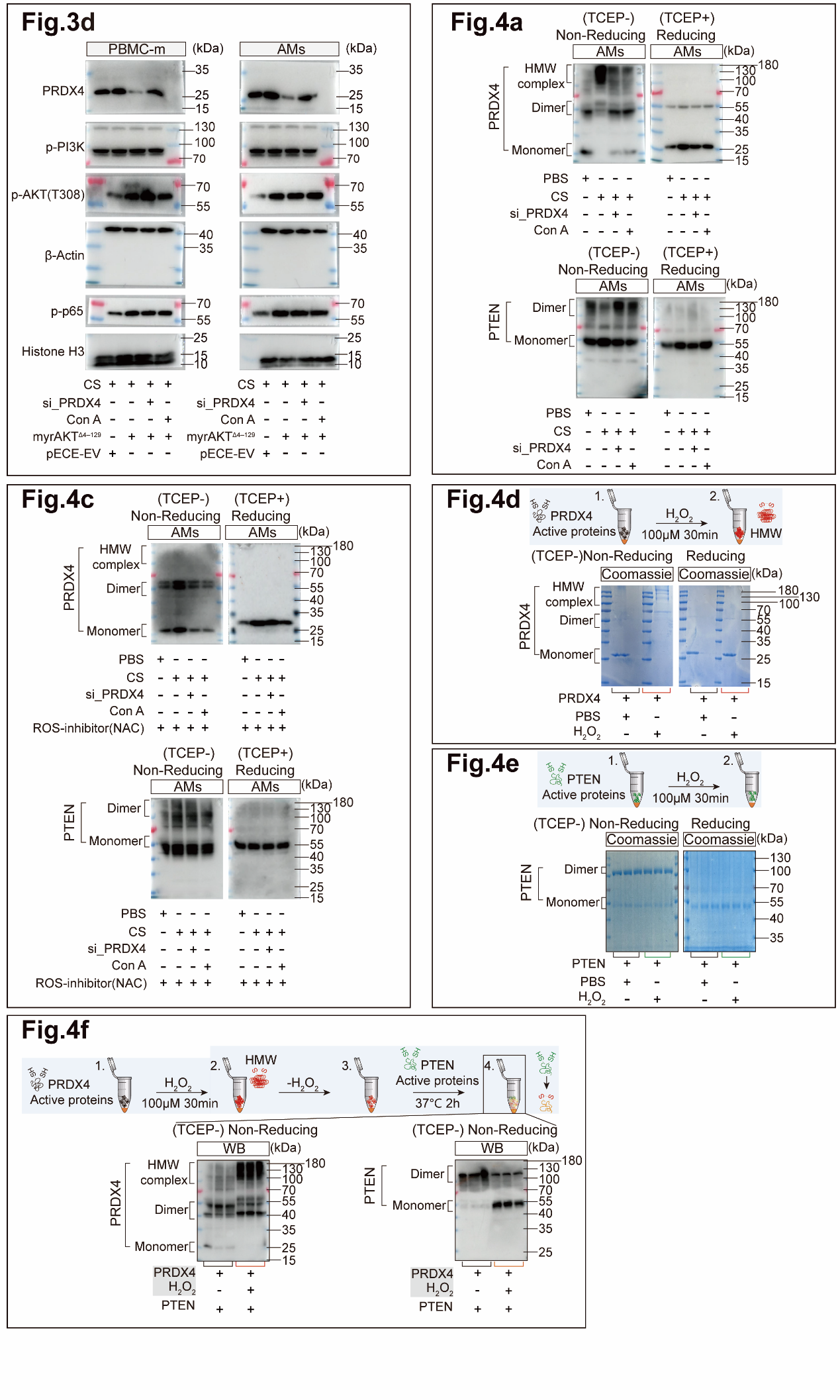


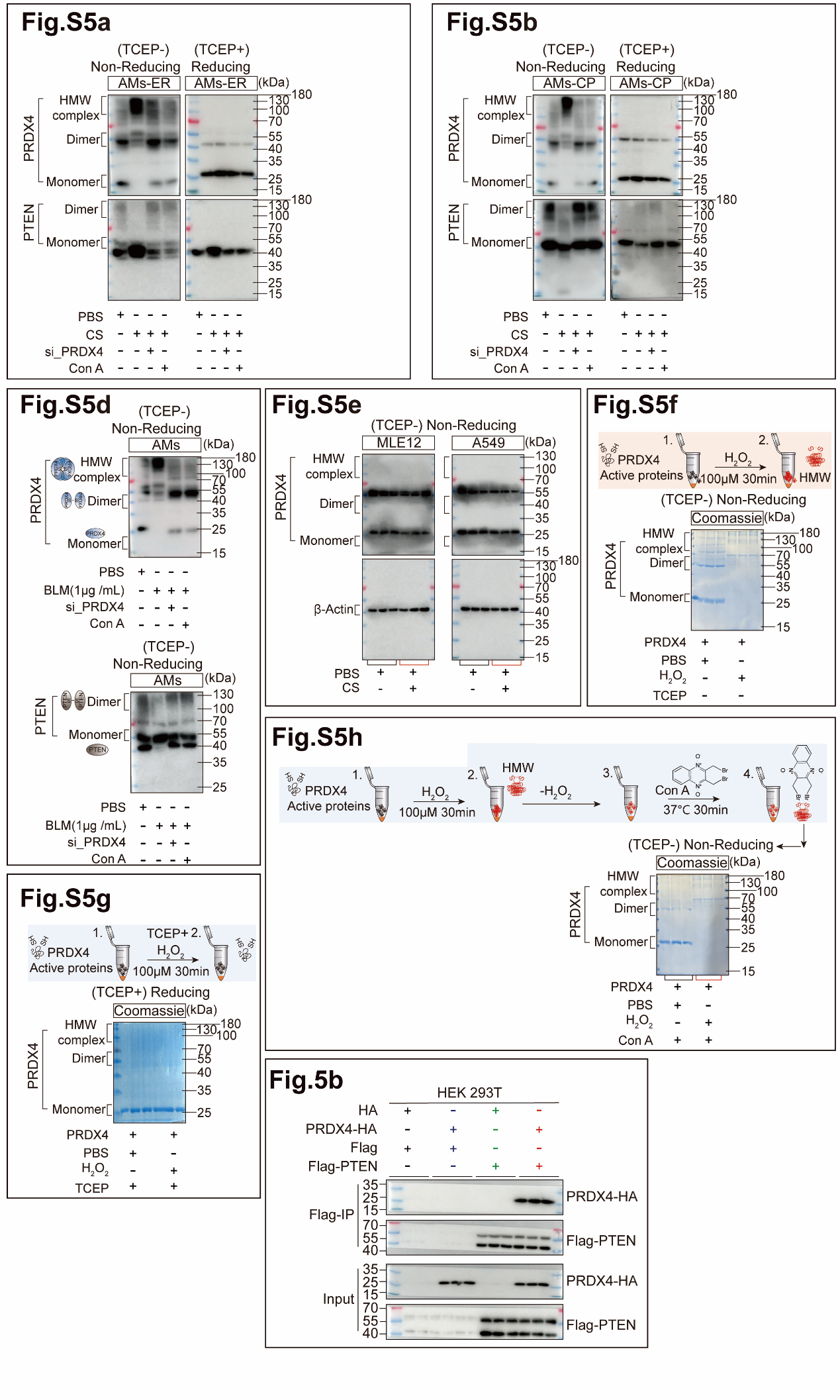


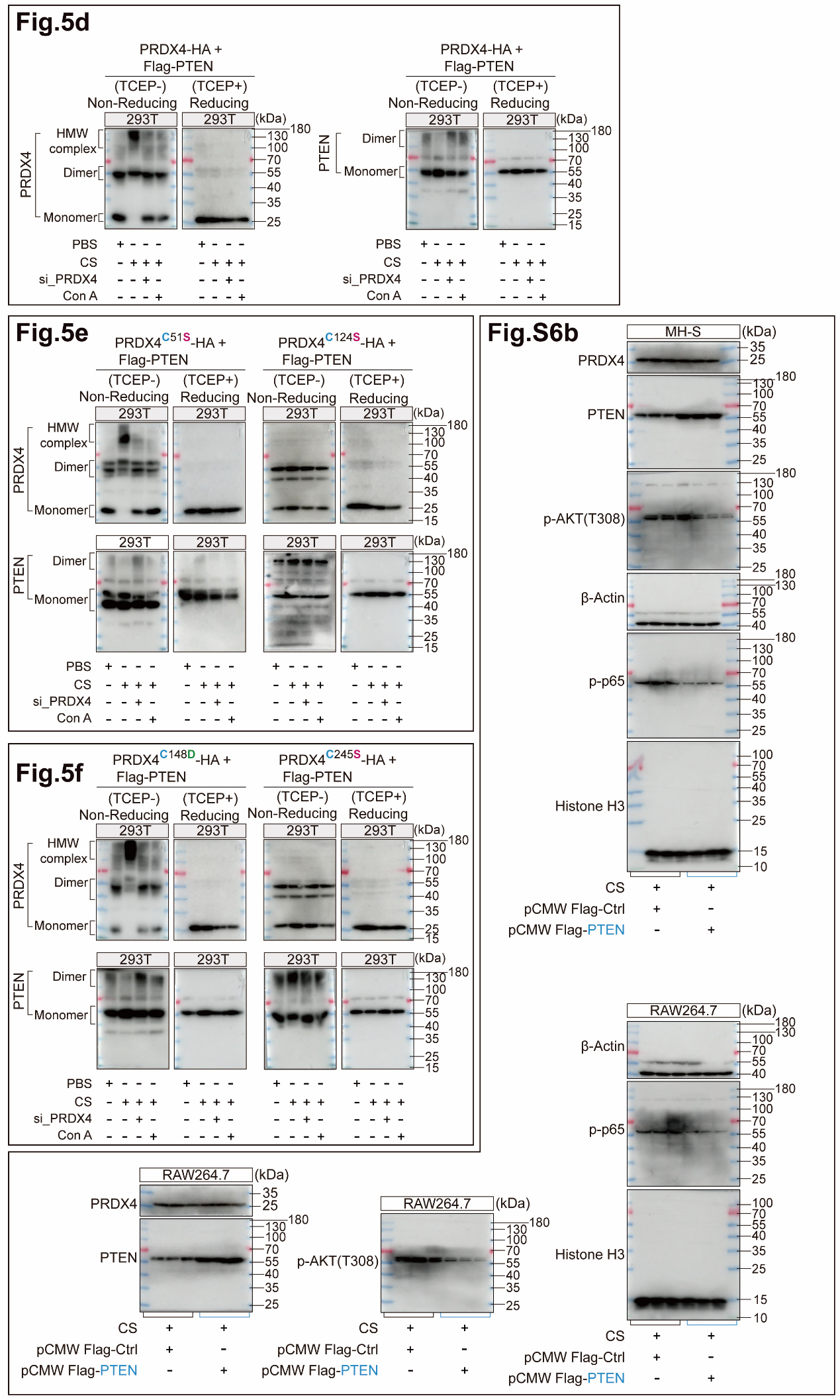


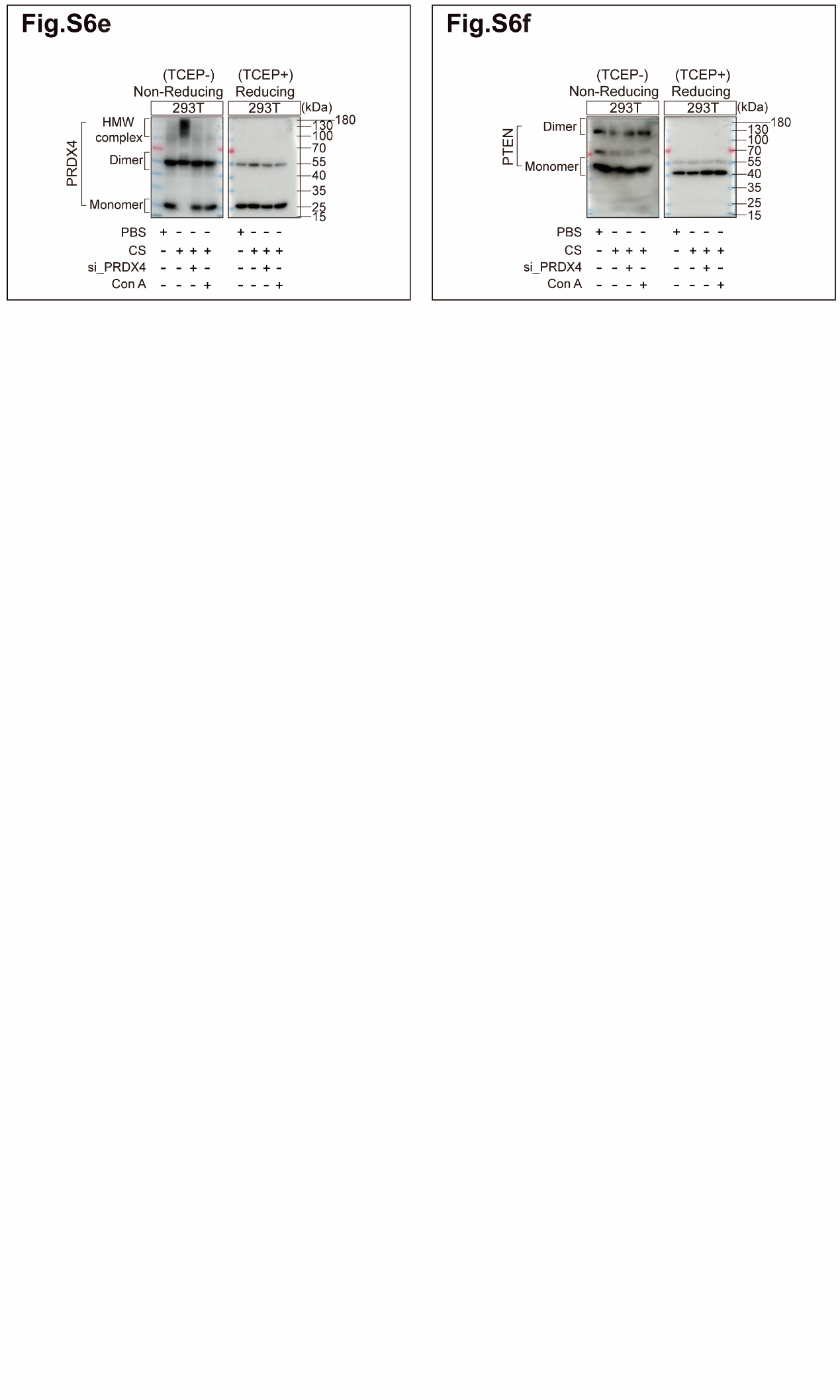

Supplement: Supplementary file 2 — Supplementary information [file 41392_2025_2454_MOESM2_ESM.docx]
